# Supplementary material for: DNA methylation atlas of the mouse brain at single-cell resolution
Source: Nature. 2021 Oct 6;598(7879):120–8. doi: 10.1038/s41586-020-03182-8 (PMC8494641; doi:10.1038/s41586-020-03182-8)
Supplement: Supplementary file 1 — This file contains 5 Supplementary Notes, which contains additional details to the text and data presented in the main text. [file 41586_2020_3182_MOESM1_ESM.pdf]

nature portfolio

https://doi.org/10.1038/s41586-020-03182-8

Supplementary information

DNA methylation atlas of the mouse brain at single-cell resolution

In the format provided by the authors and unedited

Table of Contents

Supplementary Note 1 - Neuronal Subtype Vignettes 1

Methylome similarity between Indusium Griseum (IG) and Hippocampal region CA2 neuron subtypes 1

Methylation signatures of striatal medium spiny neurons located in patch compartments 2

Supplementary Note 2 - Estimate subtype CG-DMR false discovery rate 2

Supplementary Note 3 - Total Impact score summarizes variation of each gene or motif 3

Supplementary Note 4 - Spatial axis of DG granule cells 4

Supplementary Note 5 - Artificial Neuron Network trained for predicting non-neuronal cells. 4

Supplementary Notes References 5

Supplementary Note 1 - Neuronal Subtype Vignettes

Methylome similarity between Indusium Griseum (IG) and Hippocampal region CA2 neuron subtypes

Nearly all hippocampal excitatory neurons formed their own clusters separately from cells in other brain regions (Extended Data Fig. 1g), except the IG-CA2 neurons (745 cells, three subtypes) (Extended Data Fig. 4a). Several markers of the hippocampal region CA2[1] (e.g., *Pcp4*[2], *Cacng5*[3], *Ntf3*[4]) were marked by low gene body mCH (Extended Data Fig. 4c) in IG-CA2 subtypes. However, one subtype "IG-CA2 Xpr1" (subtype 1 in Extended Data Fig. 2a, 152 cells) was located in ACA (72 cells) and Lateral Septal Complex (LSX, 71 cells), which are anatomically distinct from the hippocampus (Extended Data Fig. 1). In-situ hybridization (ISH) data from the Allen Brain Atlas (ABA) of *Ntf3* (Extended Data Fig. 4e) indicates that those cells potentially come from the IG region[5], which is a thin layer of gray matter lying dorsal to the corpus callosum at the base of the anterior half of the cingulate cortex, included in the ACA and LSX dissection regions (Extended Data Fig. 4b). With the power of single-cell epigenomic profiling, we are able to classify cells from this region without additional dissection. Moreover, the similarity of their DNA methylomes indicates the possible functional and/or developmental

relationship between the CA2 and IG excitatory neurons. Finally, the hypo-mCG regions surrounding the marker genes like *Ntf3* (Extended Data Fig. 4d) identify candidate cell-type-specific enhancers which may further our understanding of how specific gene expression programs are regulated in these structures.

Methylation signatures of striatal medium spiny neurons located in patch compartments

A major GABAergic inhibitory cell type in the striatum, the *Drd1*+ medium spiny neuron (MSN-D1) from the caudoputamen (CP, dorsal), and nucleus accumbens (ACB, ventral), is further separated into four subtypes (Extended Data Fig. 4f, k, n). Two subtypes "MSN-D1 Plxnc1" (subtype 3 in Extended Data Fig. 4f) and "MSN-D1 Ntn1" (subtype 4 in Extended Data Fig. 4f), mainly (79%) from the ACB, are further separated by location along the anterior-posterior axis (Extended Data Fig. 4g, l, based on dissection), indicating spatial diversity may exist in addition to the canonical dorsal-ventral gradient of the striatum[6,7]. "MSN-D1 Khdrbs3" and "MSN-D1 Hrh1" subtypes are mostly (94%) from CP dissections, one of them marked by gene *Khdrbs3* and its potential regulatory elements (Extended Data Fig. 4h-j) corresponds to the neurochemically defined patch[8] compartments in CP. Here the methylome profiling data provides evidence of previously unseen spatial epigenetic diversity in the striatum D1 neurons, which is also observed in the other major type MSN-D2 (*Drd2*$^{+}$, Extended Data Fig. 4m, o) of the striatum.

Supplementary Note 2 - Estimate subtype CG-DMR false discovery rate

To estimate the FDR for DMRs, we randomly partitioned the cells into the same number of groups as the number of clusters. We used methylpy DMRfind[9] (Methods) and the same filter as above to identify the DMRs between these random groups (shuffle-DMRs). 105,310 shuffle-DMRs were identified, compared to 3,947,795 subtype-DMRs, so the average FDR is about 2.7%. For each DMR, the effect size was calculated by subtracting the minimum mCG fraction across samples from the samples' robust mean. We then divided the DMRs into different groups based on the number of DMSs and the effect size of the DMR and computed the FDR within each group (Extended Data Fig. 6a, b). Most (93%) of the shuffle-DMRs only have a single DMS (no other DMS within ± 250 bp), while this proportion decreases to 35% for subtype-DMRs. When the effect size is greater than 0.3, the FDR for DMS = 1 bins range from

0.071 to 0.093. The FDR for remaining bins having DMS > 1 is close to or well below 0.01 (Extended Data Fig. 6c).

To test whether the 1,645,355 (35%) single-DMS subtype-DMRs (sDMRs) are biologically meaningful, we used the shuffle-DMR as background regions and performed motif enrichment analysis on sDMRs and multi-DMS subtype-DMRs (mDMRs) respectively. We found 108 / 174 motifs enriched in mDMRs are also enriched in sDMRs, and the odds ratio of all mDMR-enriched motifs are highly correlated (Extended Data Fig. 6d, Pearson's r=0.86, P = 1e-53). These results indicate that although single-DMS DMRs are noisier than multi-DMS DMRs, they are likely biologically relevant. Removing single-DMS DMRs would improve the overall FDR from 3.0% to 0.3%, with the cost of reducing the power to identify true positives. We provided the number of DMRs or each subtype using different filtering criteria in Supplementary Table 12.

Supplementary Note 3 - Total Impact score summarizes variation of each gene or motif

Beyond identifying specific cell subtype characteristics, we hypothesized that ranking of genes or motifs by methylation variation may provide a route toward understanding their relative importance in cell type diversification and/or function. Thus, for each gene or motif, we calculated a total impact (TI) score to summarize their variation among the subtypes, using all the branch-specific impact scores (Extended Data Fig. 7e bottom right, Methods). Genes (Excitatory: Extended Data Fig. 7f, g; Inhibitory: Extended Data Fig. 7i, j) or motifs (Excitatory: Extended Data Fig. 7h; Inhibitory: Extended Data Fig. 7k) with higher TI scores impact more branches of the phylogeny, thus are predicted to have a higher importance in distinguishing multiple subtypes.

Intriguingly, by comparing the TI scores of genes and motifs calculated from the inhibitory and excitatory phylogenies, we found more TF genes and motifs having large TI scores in both cell classes than specific to either one (Extended Data Fig. 7l). For instance, *Bcl11b* distinguishes "OLF-Exc" and Isocortex IT neurons in the excitatory lineage and distinguishes "CGE-Lamp5" and "CGE-Vip" in the inhibitory lineage. Similarly, *Satb1* separates IT-L4 from IT-L2/3, and MGE from CGE in excitatory and inhibitory cells, respectively. These findings indicate broad repurposing of TFs for cell-type specification among distinct developmental lineages.

In contrast, we also find TF genes and motifs only having large TI scores in one cell class (Extended Data Fig. 7l, left panel). For example, the *Tshz1* gene body mCH shows a striking difference of diversity between excitatory and inhibitory cells (Extended Data Fig. 7m), suggesting that it may function in shaping inhibitory subtypes, but not excitatory subtypes. Similarly, bHLH DNA binding motifs show much higher TI scores for excitatory subtypes compared with inhibitory (Extended Data Fig. 6l, right panel). While genes in this TF family such as *Neurod1/2* have long been known to participate in excitatory neuron development, they have not been reported to regulate GABAergic neuron differentiation[10].

Supplementary Note 4 - Spatial axis of DG granule cells

Neurogenesis is differentially distributed across the dorsal-ventral axis of the DG, with newborn neurons enriched in the dorsal pole of the DG[11,12]. Bulk tissue samples dissected from the dorsal DG had ~2-fold lower global mCH compared to the ventral DG, suggesting that the gradient in global mCH we observed could be related to their dorsal-ventral location[13]. Consistent with this, dorsal-hypo-DMRs from the bulk tissue samples[13] overlapped more with +DMRs (15% overlap, $P < 0.001$, two-sided permutation test) than -DMRs (4%, $P < 0.001$) (Extended Data Fig. 10e). Similarly, ventral-hypo-DMRs overlapped more with -DMRs (13%, $P < 0.001$) than +DMRs (1.7%, $P = 0.004$). These moderate overlaps suggest that the axis of the global mCH gradient can be partially explained by the traditionally defined dorsal-ventral axis. Further understanding of the relationship between these two axes will require spatially resolved transcriptome/epigenome data from cells with defined birthdates[14].

Supplementary Note 5 - Artificial Neuron Network trained for predicting non-neuronal cells.

Using the same network structure and features, we achieved 42% accuracy to predict the cell body location of non-neuronal cells. The accuracy of the model was highly cell types dependent, with stronger predictability in astrocytes (ASC, 62%) and oligodendrocytes (ODC, 52%) that are comparable to cortical inhibitory neurons (Extended Data Fig. 11d). These findings are supported by evidence that astrocytes are derived from regionally patterned radial glia[15], while the signature of oligodendrocytes depends on the local environment[16]. It is worth noting that

unsupervised clustering did not separate non-neuronal cells from different regions into different clusters, which further emphasizes the utility of supervised analyses to study the regional specificity of these cell types.

Supplementary Notes References

1. Dudek, S. M., Alexander, G. M. & Farris, S. Rediscovering area CA2: unique properties and functions. *Nat. Rev. Neurosci.* **17**, 89–102 (2016).
2. San Antonio, A., Liban, K., Ikrar, T., Tsyganovskiy, E. & Xu, X. Distinct physiological and developmental properties of hippocampal CA2 subfield revealed by using anti-Purkinje cell protein 4 (PCP4) immunostaining. *J. Comp. Neurol.* **522**, 1333–1354 (2014).
3. Fukaya, M., Yamazaki, M., Sakimura, K. & Watanabe, M. Spatial diversity in gene expression for VDCCgamma subunit family in developing and adult mouse brains. *Neurosci. Res.* **53**, 376–383 (2005).
4. Phillips, H. S., Hains, J. M., Laramee, G. R., Rosenthal, A. & Winslow, J. W. Widespread expression of BDNF but not NT3 by target areas of basal forebrain cholinergic neurons. *Science* **250**, 290–294 (1990).
5. Adamek, G. D., Shipley, M. T. & Sanders, M. S. The indusium griseum in the mouse: architecture, Timm's histochemistry and some afferent connections. *Brain Res. Bull.* **12**, 657–668 (1984).
6. Heimer, L. & Wilson, R. D. The subcortical projections of the allocortex: similarities in the neural connections of the hippocampus, the piriform cortex and the neocortex. Santini M, editor. *Perspectives in neurobiology* (1975).
7. Voorn, P., Vanderschuren, L. J. M. J., Groenewegen, H. J., Robbins, T. W. & Pennartz, C. M. A. Putting a spin on the dorsal-ventral divide of the striatum. *Trends Neurosci.* **27**, 468–474 (2004).
8. Smith, J. B. *et al.* Genetic-Based Dissection Unveils the Inputs and Outputs of Striatal Patch and Matrix Compartments. *Neuron* **91**, 1069–1084 (2016).
9. Schultz, M. D. *et al.* Human body epigenome maps reveal noncanonical DNA methylation variation. *Nature* **523**, 212–216 (2015).
10. Ross, S. E., Greenberg, M. E. & Stiles, C. D. Basic helix-loop-helix factors in cortical

development. *Neuron* **39**, 13–25 (2003).

11. Snyder, J. S., Radik, R., Wojtowicz, J. M. & Cameron, H. A. Anatomical gradients of adult neurogenesis and activity: young neurons in the ventral dentate gyrus are activated by water maze training. *Hippocampus* **19**, 360–370 (2009).
12. Bekiari, C., Grivas, I., Tsingotjidou, A. & Papadopoulos, G. C. Adult neurogenesis and gliogenesis in the dorsal and ventral canine hippocampus. *J. Comp. Neurol.* **528**, 1216–1230 (2020).
13. Zhang, T.-Y. *et al.* Environmental enrichment increases transcriptional and epigenetic differentiation between mouse dorsal and ventral dentate gyrus. *Nat. Commun.* **9**, 298 (2018).
14. Habib, N. *et al.* Div-Seq: Single-nucleus RNA-Seq reveals dynamics of rare adult newborn neurons. *Science* **353**, 925–928 (2016).
15. Bayraktar, O. A., Fuentealba, L. C., Alvarez-Buylla, A. & Rowitch, D. H. Astrocyte development and heterogeneity. *Cold Spring Harb. Perspect. Biol.* **7**, a020362 (2014).
16. Floriddia, E. M., Zhang, S. & van Bruggen, D. Distinct oligodendrocyte populations have different spatial distributions and injury-specific responses. *bioRxiv* (2019).
