# Supplementary material for: DNA methylation atlas of the mouse brain at single-cell resolution
Source: Nature. 2021 Oct 6;598(7879):120–8. doi: 10.1038/s41586-020-03182-8 (PMC8494641; doi:10.1038/s41586-020-03182-8)
Supplement: Supplementary file 2 — Reporting Summary [file 41586_2020_3182_MOESM2_ESM.pdf]

nature research

Corresponding author(s): Joseph R. Ecker

Last updated by author(s): Nov 7, 2020

Reporting Summary

Nature Research wishes to improve the reproducibility of the work that we publish. This form provides structure for consistency and transparency in reporting. For further information on Nature Research policies, see our Editorial Policies and the Editorial Policy Checklist.

Statistics

For all statistical analyses, confirm that the following items are present in the figure legend, table legend, main text, or Methods section.

n/a	Confirmed	
☐	☒	The exact sample size (*n*) for each experimental group/condition, given as a discrete number and unit of measurement
☐	☒	A statement on whether measurements were taken from distinct samples or whether the same sample was measured repeatedly
☐	☒	The statistical test(s) used AND whether they are one- or two-sided<br>*Only common tests should be described solely by name; describe more complex techniques in the Methods section.*
☐	☒	A description of all covariates tested
☐	☒	A description of any assumptions or corrections, such as tests of normality and adjustment for multiple comparisons
☐	☒	A full description of the statistical parameters including central tendency (e.g. means) or other basic estimates (e.g. regression coefficient) AND variation (e.g. standard deviation) or associated estimates of uncertainty (e.g. confidence intervals)
☐	☒	For null hypothesis testing, the test statistic (e.g. *F*, *t*, *r*) with confidence intervals, effect sizes, degrees of freedom and *P* value noted<br>*Give P values as exact values whenever suitable.*
☒	☐	For Bayesian analysis, information on the choice of priors and Markov chain Monte Carlo settings
☒	☐	For hierarchical and complex designs, identification of the appropriate level for tests and full reporting of outcomes
☐	☒	Estimates of effect sizes (e.g. Cohen's *d*, Pearson's *r*), indicating how they were calculated

Our web collection on statistics for biologists contains articles on many of the points above.

Software and code

Policy information about availability of computer code

Data collection	BD Influx Software v1.2.0.142 (flow cytometry), Freedom EVOware v2.7 (library preparation), Illumina MiSeq control software v3.1.0.13 and NovaSeq 6000 control software v1.6.0/RTA v3.4.4 (sequencing), Olympus cellSens Dimension 1.8 (image acquisition)
Data analysis	bedtools 2.27, methylpy 1.4.2, scanpy 1.4.3, juicer tools 1.14.08, REPTILE (https://github.com/yupenghe/REPTILE.git), scHiCluster (https://github.com/zhoujt1994/scHiCluster.git)<br>the mapping pipeline for snmC-seq2 data: https://cemba-data.readthedocs.io/en/latest/, including the following packages: bismark 0.20, bowtie2 2.3, cutadapt 1.18, picard 2.18, samtools 1.9, htslib 1.9;<br>The ALLCools package for post-mapping analysis and snmC-seq2 related data structure: https://github.com/lhqing/ALLCools;<br>The jupyter notebooks for specific analysis: https://github.com/lhqing/mouse_brain_2020.

For manuscripts utilizing custom algorithms or software that are central to the research but not yet described in published literature, software must be made available to editors and reviewers. We strongly encourage code deposition in a community repository (e.g. GitHub). See the Nature Research guidelines for submitting code & software for further information.

Data

Policy information about availability of data

All manuscripts must include a data availability statement. This statement should provide the following information, where applicable:

- Accession codes, unique identifiers, or web links for publicly available datasets
- A list of figures that have associated raw data
- A description of any restrictions on data availability

Single-cell raw and processed data included in this study were deposited to NCBI GEO/SRA with accession number GSE132489 (each experiment has a separate accession number recorded in GSE132489, see Supplementary Table 12), and to the NeMO archive: https://assets.nemoarchive.org/dat-vmivr5x. Single-cell

methylation data can be visualized at the Brain Cell Methylation Viewer: http://neomorph.salk.edu/omb/home. Cluster merged methylome profiles can be visualized at http://neomorph.salk.edu/mouse_brain.php. Other datasets used in the paper include single-nuclei ATAC-Seq data from http://catlas.org, mouse embryo forebrain development data from ENCODE portal (https://www.encodeproject.org/), the developing hippocampal single-cell RNA-seq data from GSE104323, DNA methylation and chromatin accessibility profiles for mESC from GSM723018, and JASPAR 2020 CORE vertebrates database from http://meme-suite.org/db/motifs.

Field-specific reporting

Please select the one below that is the best fit for your research. If you are not sure, read the appropriate sections before making your selection.

☒ Life sciences ☐ Behavioural & social sciences ☐ Ecological, evolutionary & environmental sciences

For a reference copy of the document with all sections, see nature.com/documents/nr-reporting-summary-flat.pdf

Life sciences study design

All studies must disclose on these points even when the disclosure is negative.

Sample size	At least 3,072 nuclei (eight 384-well plates) from each dissected region (1,536 nuclei from each replicate). The sample size allowed us to obtain high coverage methylomes for each subtype, and perform confident downstream analyses.
Data exclusions	We filtered the cells based on these main mapping metrics: 1) mCCC level < 0.03, 2) overall mCG level > 0.5, 3) overall mCH level < 0.2, 4) total final reads > 500,000, 5) bismark mapping rate > 0.5. Other metrics such as genome coverage, PCR duplicates rate, index ratio were also generated and evaluated during filtering. However, after removing outliers with the main metrics 1-5, few additional outliers can be found. Note the mCCC level is used as the estimation of the upper bound of bisulfite non-conversion rate. The criterion include pre-established ones in Luo. et al 2018, and new ones to exclude additional outliers as justified in the manuscript.
Replication	Each dissected region has at least two replicates, each replicate was pooled from 6-30 animals separately for nuclei preparation and downstream analyses. Data are highly consistent between replicates (Extended Data Fig. 2d-g).
Randomization	Randomization is not applicable, since the cells collected are random by nature.
Blinding	Blinding is not applicable, since all data are collected from mice.

Reporting for specific materials, systems and methods

We require information from authors about some types of materials, experimental systems and methods used in many studies. Here, indicate whether each material, system or method listed is relevant to your study. If you are not sure if a list item applies to your research, read the appropriate section before selecting a response.

Materials & experimental systems

n/a	Involved in the study	
☐	☒	Antibodies
☒	☐	Eukaryotic cell lines
☒	☐	Palaeontology and archaeology
☐	☒	Animals and other organisms
☒	☐	Human research participants
☒	☐	Clinical data
☒	☐	Dual use research of concern

Methods

n/a	Involved in the study	
☒	☐	ChIP-seq
☐	☒	Flow cytometry
☒	☐	MRI-based neuroimaging

Antibodies

Antibodies used	AlexaFluor488-conjugated anti-NeuN antibody (MAB377X, Millipore)
Validation	All antibodies have been previously published for use in immunohistochemistry and flow cytometry experiments. See vendor's page here: https://www.emdmillipore.com/US/en/product/Anti-NeuN-Antibody-clone-A60-Alexa-Fluor488-conjugated,MM_NF-MAB377X

Animals and other organisms

Policy information about studies involving animals; ARRIVE guidelines recommended for reporting animal research

Laboratory animals	Adult (P56) C57BL/6J male mice. Housing condition: Temperature: 21-23 C, relative humidity: 61-63%
Wild animals	the study did not involve wild animals

Field-collected samples	the study did not involve samples collected from the field
Ethics oversight	All experimental procedures using live animals were approved by the Salk Institute Animal Care and Use Committee under protocol number 18-00006.

Note that full information on the approval of the study protocol must also be provided in the manuscript.

Flow Cytometry

Plots

Confirm that:

☒ The axis labels state the marker and fluorochrome used (e.g. CD4-FITC).

☒ The axis scales are clearly visible. Include numbers along axes only for bottom left plot of group (a 'group' is an analysis of identical markers).

☒ All plots are contour plots with outliers or pseudocolor plots.

☒ A numerical value for number of cells or percentage (with statistics) is provided.

Methodology

Sample preparation	Isolated nuclei were labeled by incubation with 1:1000 dilution of AlexaFluor488-conjugated anti-NeuN antibody (MAB377X, Millipore) and a 1:1000 dilution of Hoechst 33342 at 4°C for 1 hour with continuous shaking. Fluorescence-Activated Nuclei Sorting (FANS) of single nuclei was performed using a BD Influx sorter with an 85µm nozzle at 22.5 PSI sheath pressure. Single nuclei were sorted into each well of a 384-well plate preloaded with 2 µl of Proteinase K digestion buffer (1µl M-Digestion Buffer, 0.1µl 20 µg/µl Proteinase K and 0.9µl H2O). The alignment of the receiving 384-well plate was performed by sorting sheath flow into wells of an empty plate and making adjustments based on the liquid drop position. Single-cell (1 drop single) mode was selected to ensure the stringency of sorting. For each 384-well plate, columns 1-22 were sorted with NeuN+ (488 +) gate, and column 23-24 with NeuN- (488-) gate, reaching an 11:1 ratio of NeuN+ to NeuN- nuclei.
Instrument	BD Influx
Software	BD Influx Sortware v1.2.0.142
Cell population abundance	We sort NeuN+ (488+) gate and NeuN- (488-) gate with an 11:1 ratio into each 384-well plate.
Gating strategy	Intact nuclei were first discriminated from debris by virtue of their bright DNA labeling (Hoechst Height signal) followed by light scattering profiles (Forward Scatter (FSC) Height vs Side Scatter (SSC) Height). Events with high Pulse Width measurements for FSC and SSC were then excluded as aggregates. Next, NeuN-AlexaFluor 488 positive or negative nuclei were selected, reaching an 11:1 ratio of NeuN+ to NeuN- nuclei.

☒ Tick this box to confirm that a figure exemplifying the gating strategy is provided in the Supplementary Information.
