# Supplementary material for: DNA methylation atlas of the mouse brain at single-cell resolution
Source: Nature. 2021 Oct 6;598(7879):120–8. doi: 10.1038/s41586-020-03182-8 (PMC8494641; doi:10.1038/s41586-020-03182-8)

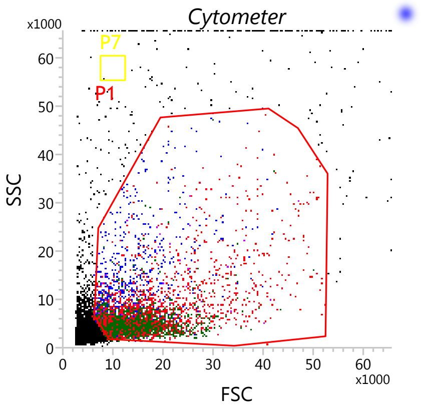

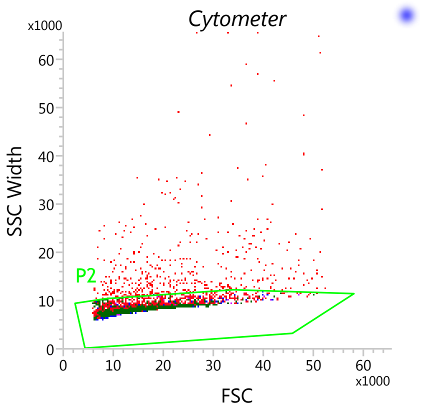

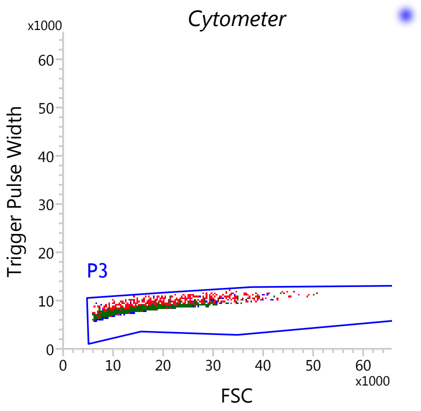

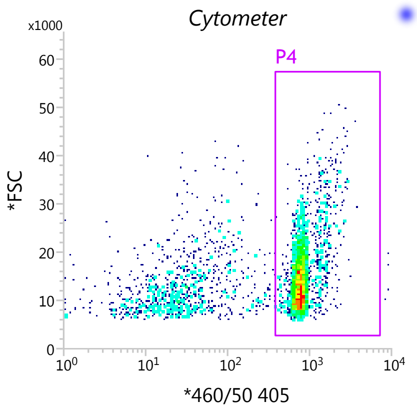

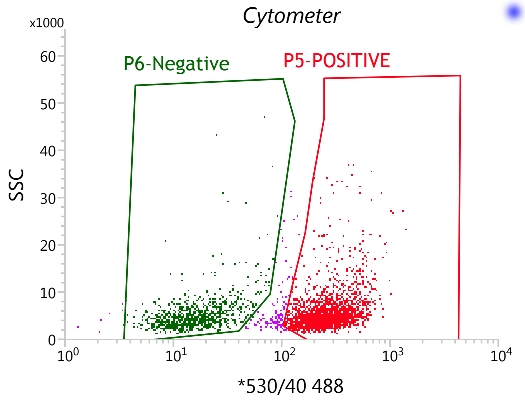

Populations: *Cytometer*

Populations	Events	% Total	% Parent
All Events	8,238	100.00%	####
P1	4,596	55.79%	55.79%
P2	4,128	50.11%	89.82%
P3	4,128	50.11%	100.00%
P4	3,244	39.38%	78.59%
P5-POSITIVE	2,240	27.19%	69.05%
P6-Negative	873	10.60%	26.91%
P7	1	0.01%	0.01%
NOT(P7)	8,237	99.99%	99.99%

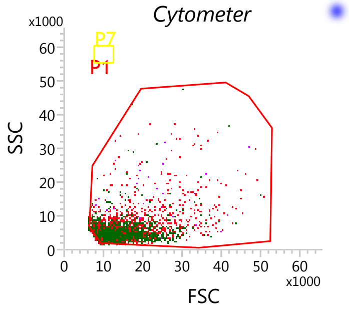

Supplement: Supplementary file 4 — This Tar/Gzip compressed file contains FANS images and gating strategies of the nuclei preparation experiments in this study. [file 41586_2020_3182_MOESM4_ESM.tgz › FANS_images/6B/CEMBA181023-6B.pdf]
