# Supplementary figures and images for: DNA methylation atlas of the mouse brain at single-cell resolution
Source: Nature. 2021 Oct 6;598(7879):120–8. doi: 10.1038/s41586-020-03182-8 (PMC8494641; doi:10.1038/s41586-020-03182-8)

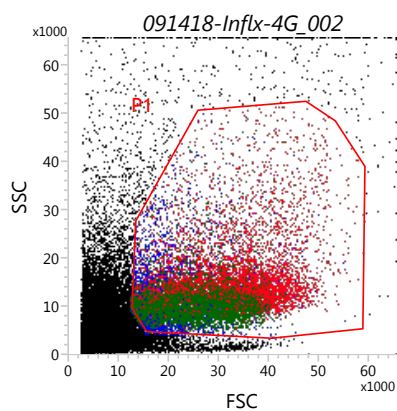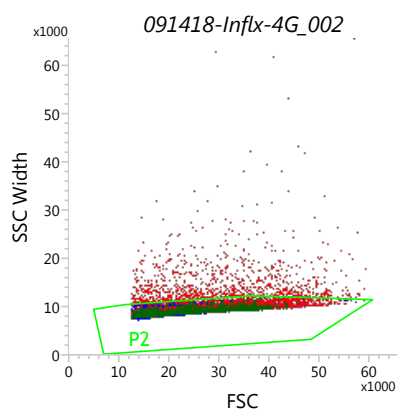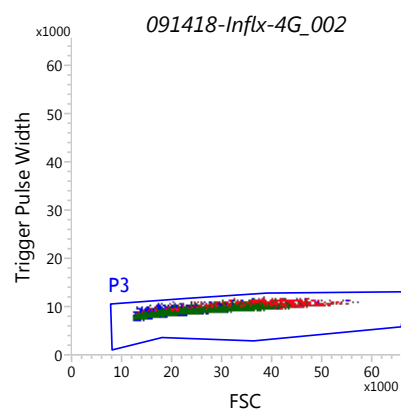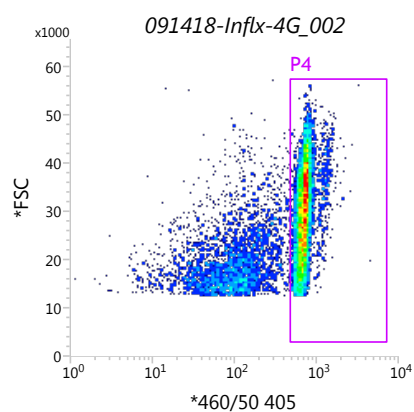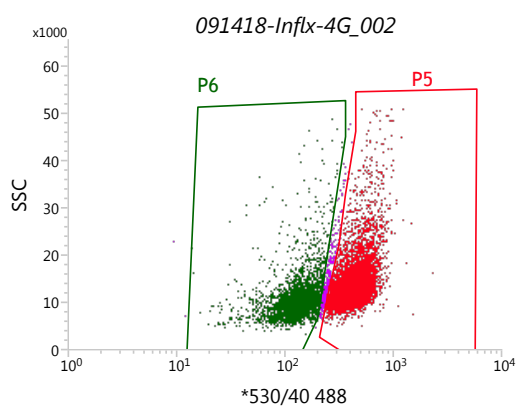

Populations: 091418-Inflx-4G\_002

| Populations | Events | % Total | % Parent |
|-------------|--------|---------|----------|
| All Events  | 56,887 | 100.00% | ####     |
| P1          | 14,222 | 25.00%  | 25.00%   |
| P2          | 12,986 | 22.83%  | 91.31%   |
| P3          | 12,986 | 22.83%  | 100.00%  |
| P4          | 10,193 | 17.92%  | 78.49%   |
| P5          | 6,517  | 11.46%  | 63.94%   |
| P6          | 3,301  | 5.80%   | 32.38%   |

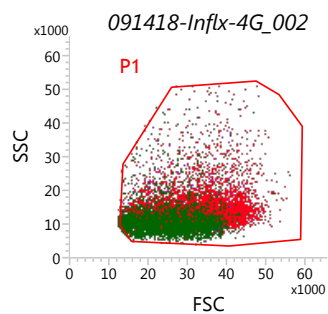

Supplement: Supplementary file 4 — This Tar/Gzip compressed file contains FANS images and gating strategies of the nuclei preparation experiments in this study. [file 41586_2020_3182_MOESM4_ESM.tgz › FANS_images/4G/CEMBA180917-4G.pdf]

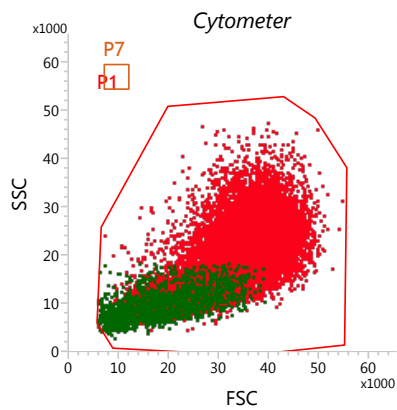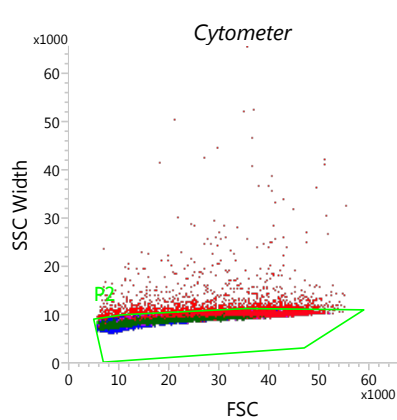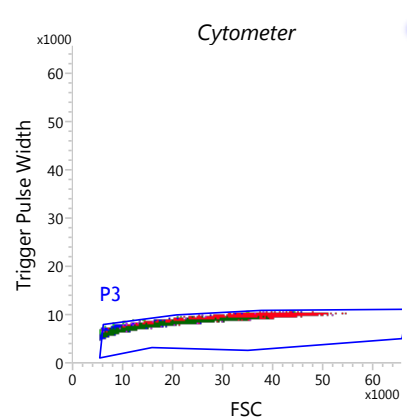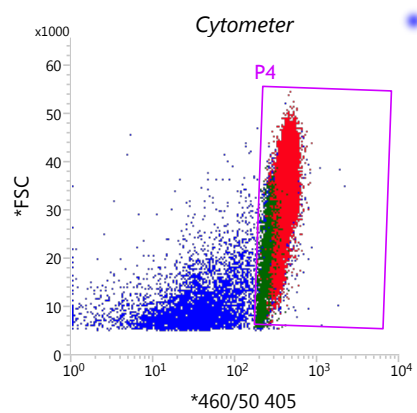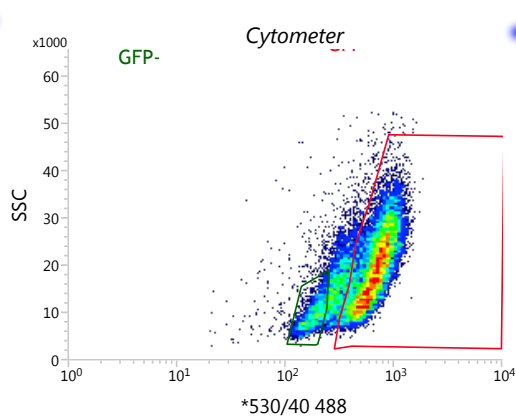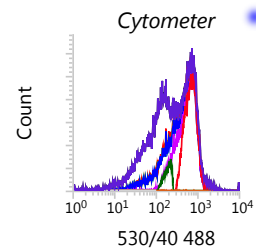

Populations: *Cytometer*

| Populations | Events | % Total | % Parent |
|-------------|--------|---------|----------|
| All Events  | 37,864 | 100.00% | ####     |
| P1          | 23,682 | 62.54%  | 62.54%   |
| P2          | 21,667 | 57.22%  | 91.49%   |
| P3          | 21,640 | 57.15%  | 99.88%   |
| P4          | 16,120 | 42.57%  | 74.49%   |
| GFP         | 11,567 | 30.55%  | 71.76%   |
| GFP-        | 1,618  | 4.27%   | 10.04%   |
| P7          | 16     | 0.04%   | 0.04%    |
| NOT(P7)     | 37,848 | 99.96%  | 99.96%   |

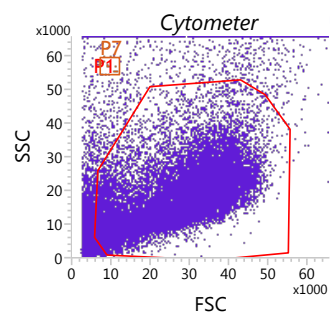

Supplement: Supplementary file 4 — This Tar/Gzip compressed file contains FANS images and gating strategies of the nuclei preparation experiments in this study. [file 41586_2020_3182_MOESM4_ESM.tgz › FANS_images/4G/CEMBA181204-4G.pdf]

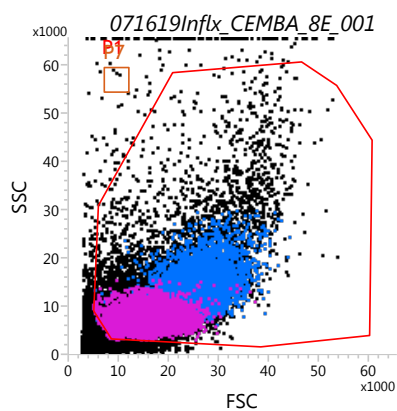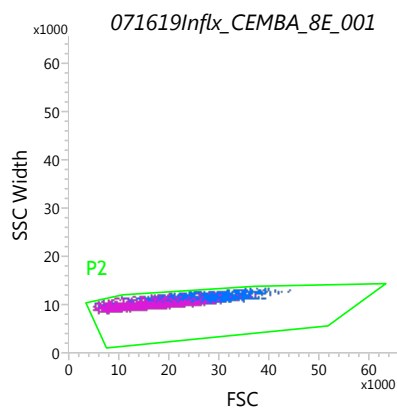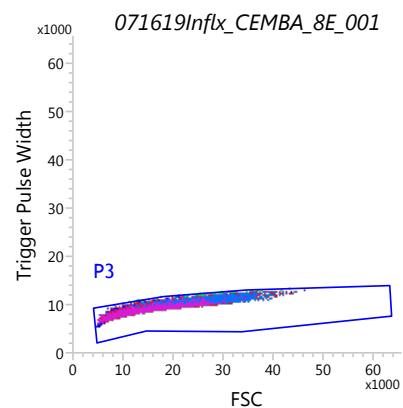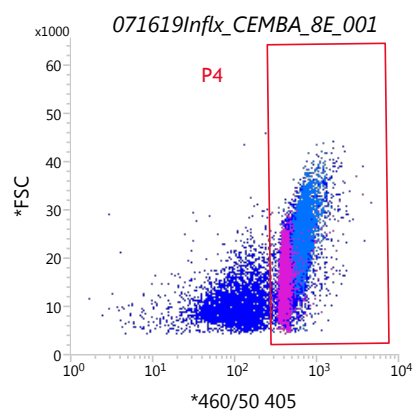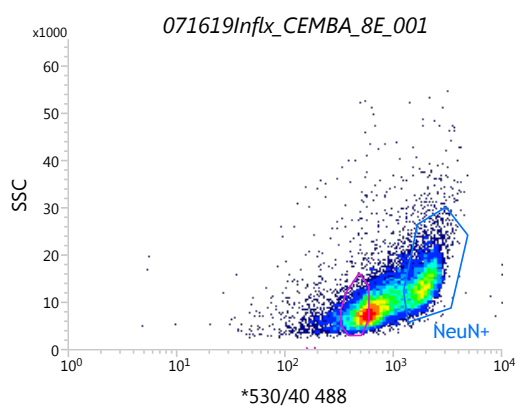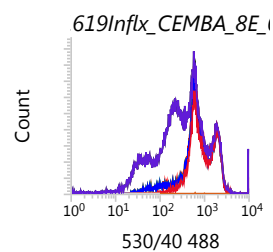

Populations: 071619Inflx\_CEMBA\_8E\_001

| Populations | Events | % Total | % Parent |
|-------------|--------|---------|----------|
| All Events  | 50,000 | 100.00% | ####     |
| P1          | 24,975 | 49.95%  | 49.95%   |
| P2          | 22,486 | 44.97%  | 90.03%   |
| P3          | 22,404 | 44.81%  | 99.64%   |
| P4          | 18,301 | 36.60%  | 81.69%   |
| NeuN+       | 5,776  | 11.55%  | 31.56%   |
| Neg         | 4,581  | 9.16%   | 25.03%   |
| P7          | 3      | 0.01%   | 0.01%    |
| NOT(P7)     | 49,997 | 99.99%  | 99.99%   |

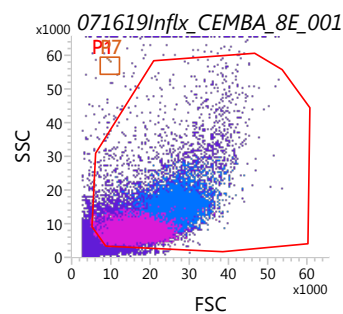

Supplement: Supplementary file 4 — This Tar/Gzip compressed file contains FANS images and gating strategies of the nuclei preparation experiments in this study. [file 41586_2020_3182_MOESM4_ESM.tgz › FANS_images/8E/CEMBA190716-8E.pdf]

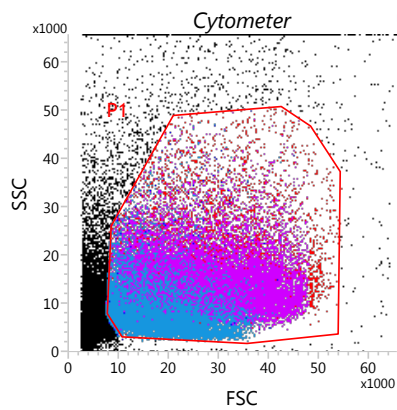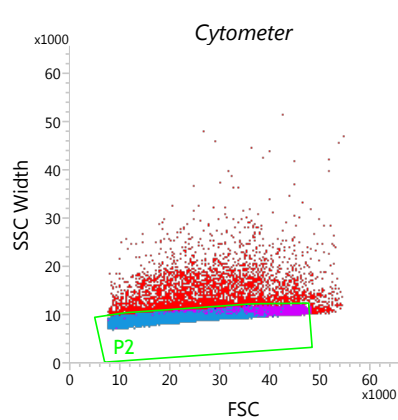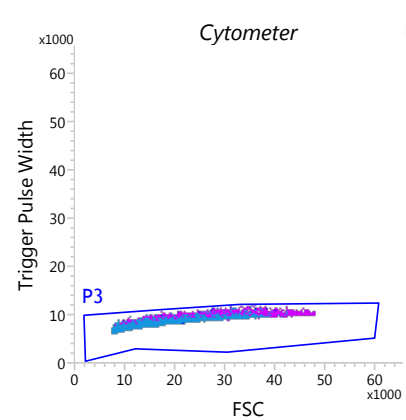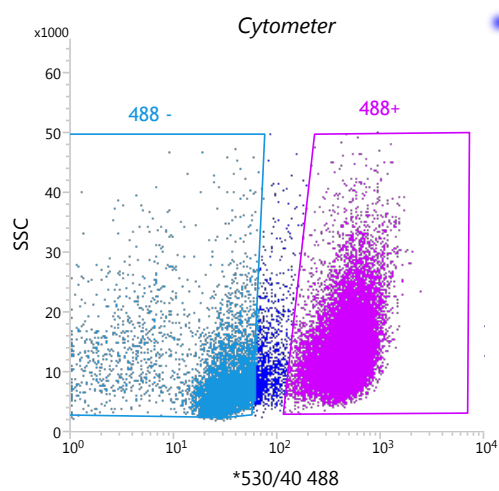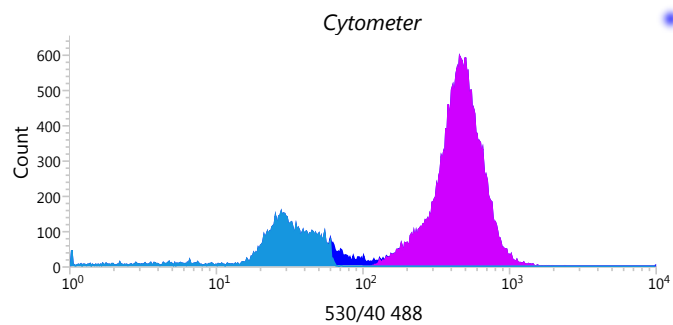

Populations: *Cytometer*

| Populations | Events | % Total | % Parent |
|-------------|--------|---------|----------|
| All Events  | 50,000 | 100.00% | ####     |
| P1          | 38,006 | 76.01%  | 76.01%   |
| P2          | 33,412 | 66.82%  | 87.91%   |
| P3          | 33,412 | 66.82%  | 100.00%  |
| 488+        | 24,796 | 49.59%  | 74.21%   |
| 488 -       | 7,527  | 15.05%  | 22.53%   |

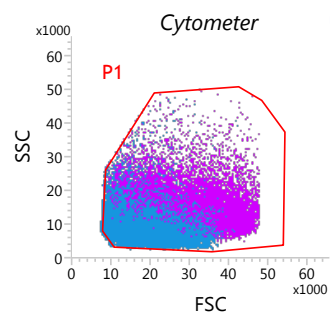

Supplement: Supplementary file 4 — This Tar/Gzip compressed file contains FANS images and gating strategies of the nuclei preparation experiments in this study. [file 41586_2020_3182_MOESM4_ESM.tgz › FANS_images/8B/CEMBA180426-8B.pdf]

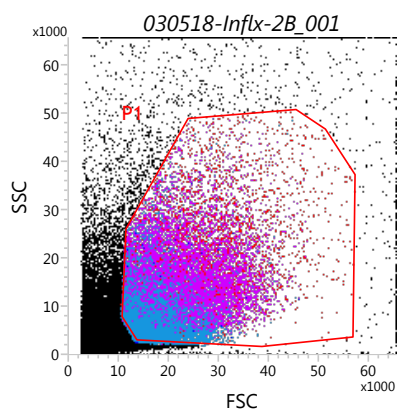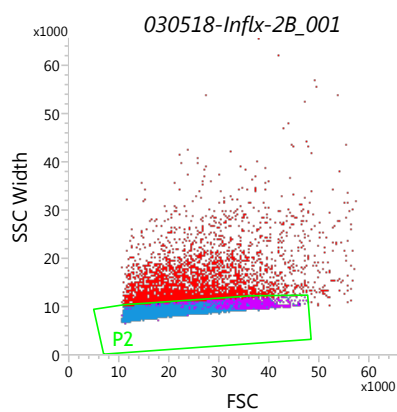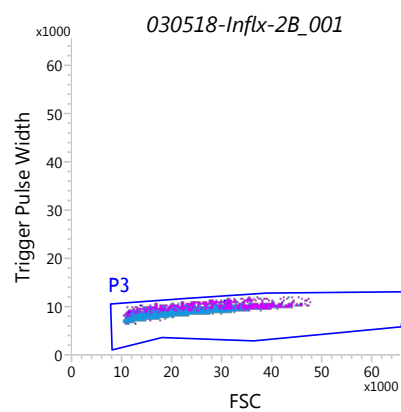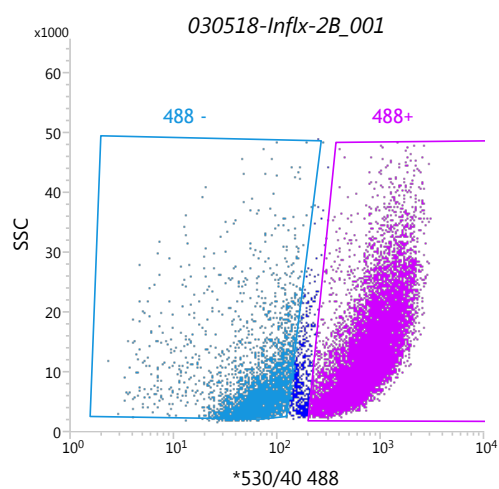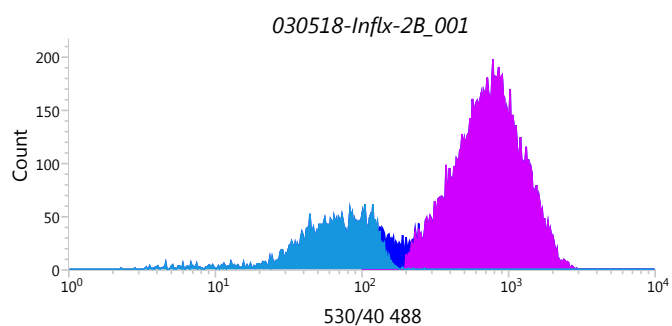

Populations: 030518-Inflx-2B\_001

| Populations | Events | % Total | % Parent |
|-------------|--------|---------|----------|
| All Events  | 39,347 | 100.00% | ####     |
| P1          | 20,996 | 53.36%  | 53.36%   |
| P2          | 16,716 | 42.48%  | 79.62%   |
| P3          | 16,716 | 42.48%  | 100.00%  |
| 488+        | 12,495 | 31.76%  | 74.75%   |
| 488 -       | 3,714  | 9.44%   | 22.22%   |

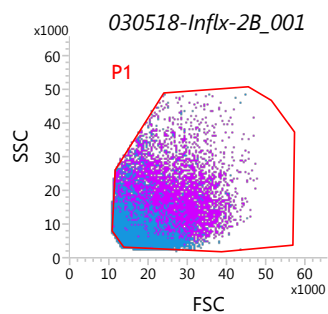

Supplement: Supplementary file 4 — This Tar/Gzip compressed file contains FANS images and gating strategies of the nuclei preparation experiments in this study. [file 41586_2020_3182_MOESM4_ESM.tgz › FANS_images/2B/CEMBA180305-2B.pdf]

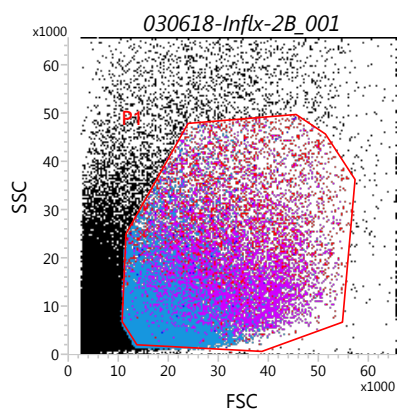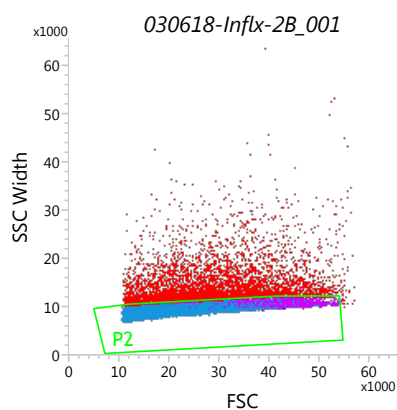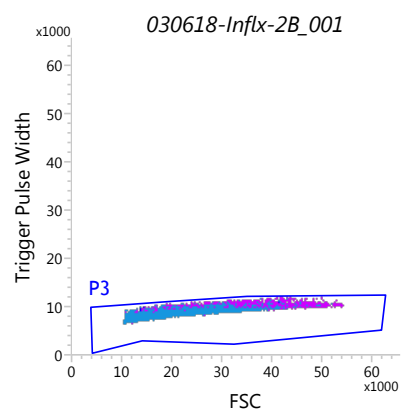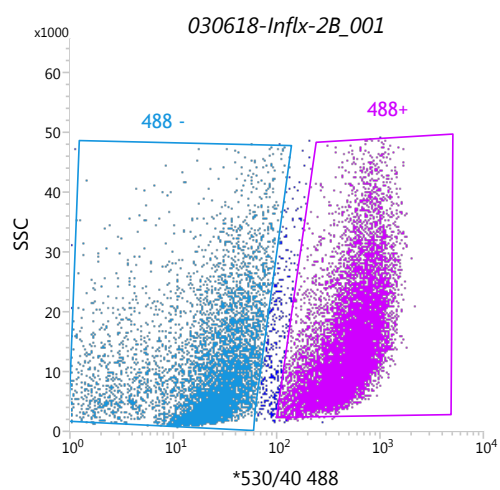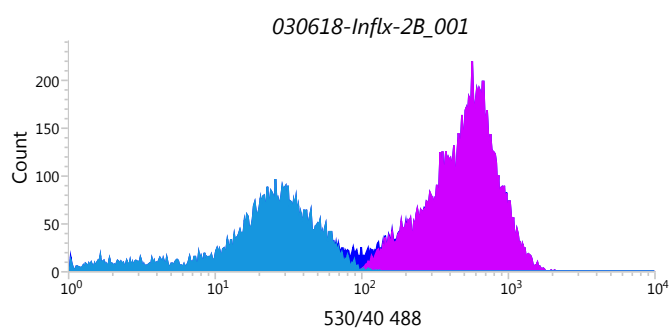

Populations: 030618-Inflx-2B\_001

| Populations | Events | % Total | % Parent |
|-------------|--------|---------|----------|
| All Events  | 64,703 | 100.00% | ####     |
| P1          | 24,801 | 38.33%  | 38.33%   |
| P2          | 18,931 | 29.26%  | 76.33%   |
| P3          | 18,931 | 29.26%  | 100.00%  |
| 488+        | 11,693 | 18.07%  | 61.77%   |
| 488 -       | 6,869  | 10.62%  | 36.28%   |

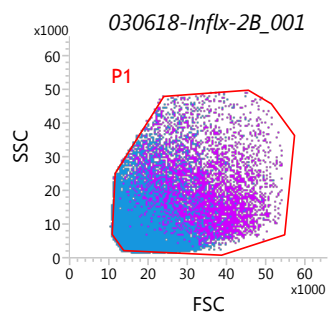

Supplement: Supplementary file 4 — This Tar/Gzip compressed file contains FANS images and gating strategies of the nuclei preparation experiments in this study. [file 41586_2020_3182_MOESM4_ESM.tgz › FANS_images/2B/CEMBA180306-2B.pdf]

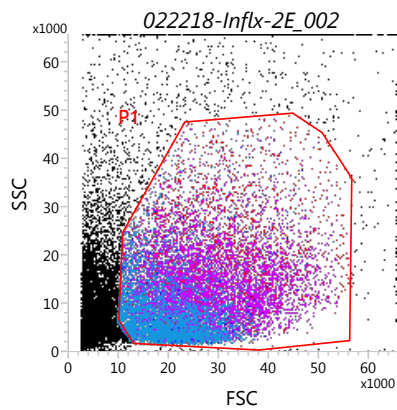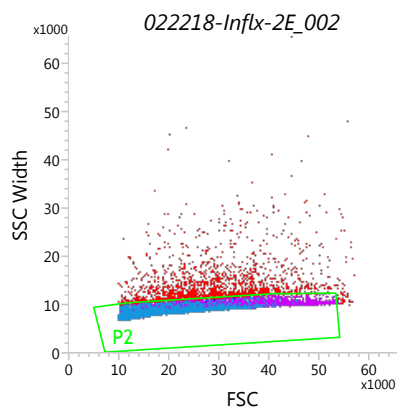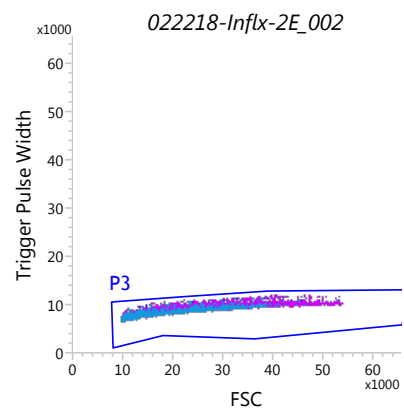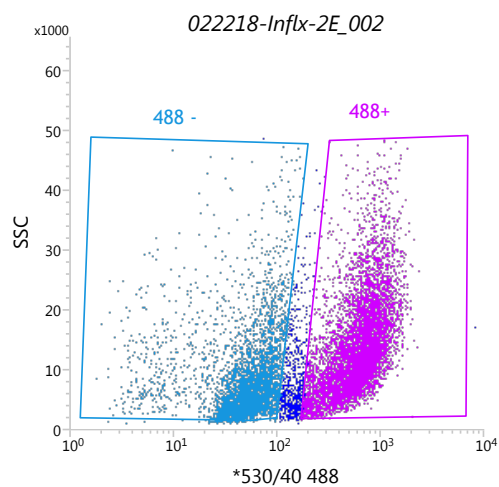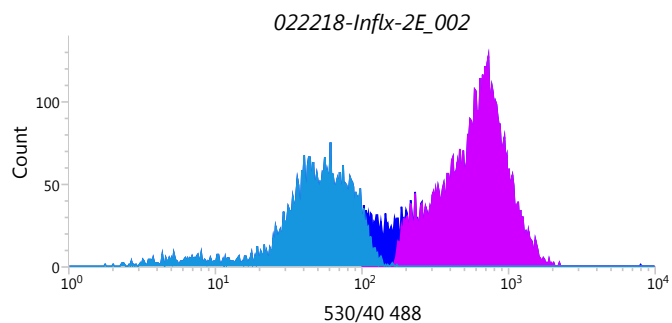

Populations: 022218-Inflx-2E\_002

| Populations | Events | % Total | % Parent |
|-------------|--------|---------|----------|
| All Events  | 23,878 | 100.00% | ####     |
| P1          | 13,217 | 55.35%  | 55.35%   |
| P2          | 11,338 | 47.48%  | 85.78%   |
| P3          | 11,338 | 47.48%  | 100.00%  |
| 488+        | 6,554  | 27.45%  | 57.81%   |
| 488 -       | 4,187  | 17.53%  | 36.93%   |

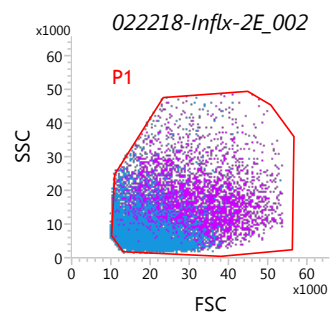

Supplement: Supplementary file 4 — This Tar/Gzip compressed file contains FANS images and gating strategies of the nuclei preparation experiments in this study. [file 41586_2020_3182_MOESM4_ESM.tgz › FANS_images/2E/CEMBA180222-2E.pdf]

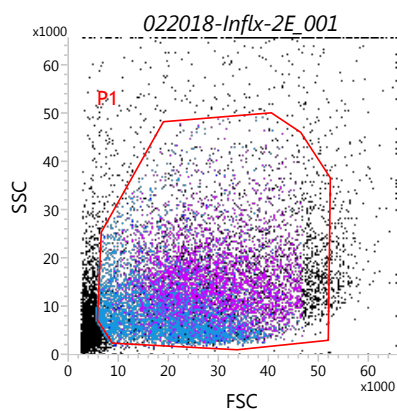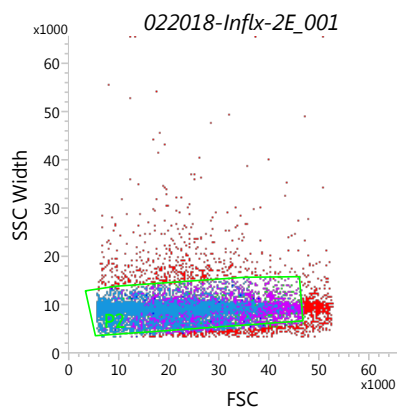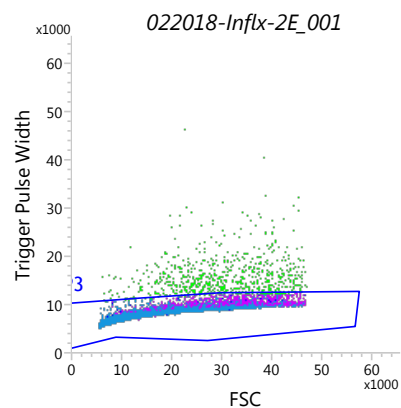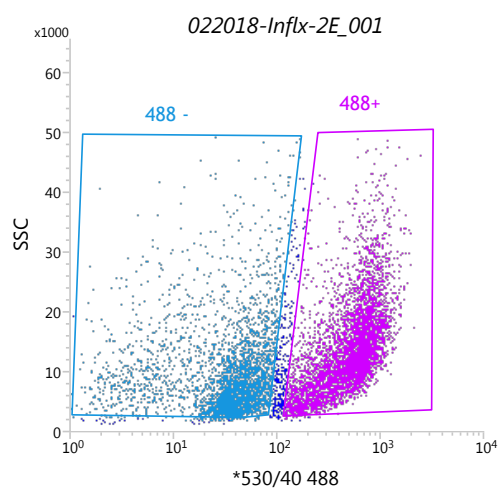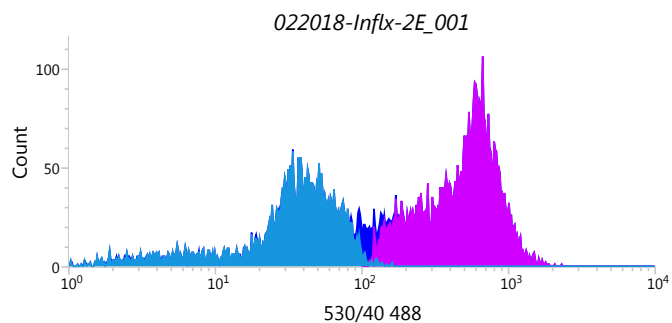

Populations: 022018-Inflx-2E\_001

| Populations | Events | % Total | % Parent |
|-------------|--------|---------|----------|
| All Events  | 14,356 | 100.00% | ####     |
| P1          | 10,862 | 75.66%  | 75.66%   |
| P2          | 9,507  | 66.22%  | 87.53%   |
| P3          | 8,796  | 61.27%  | 92.52%   |
| 488+        | 4,952  | 34.49%  | 56.30%   |
| 488 -       | 3,526  | 24.56%  | 40.09%   |

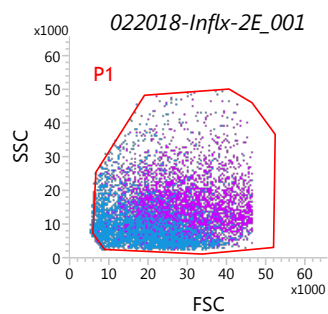

Supplement: Supplementary file 4 — This Tar/Gzip compressed file contains FANS images and gating strategies of the nuclei preparation experiments in this study. [file 41586_2020_3182_MOESM4_ESM.tgz › FANS_images/2E/CEMBA180220-2E.pdf]

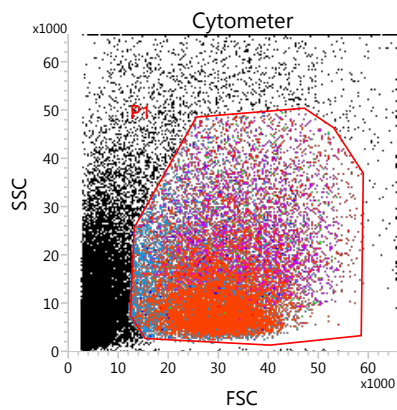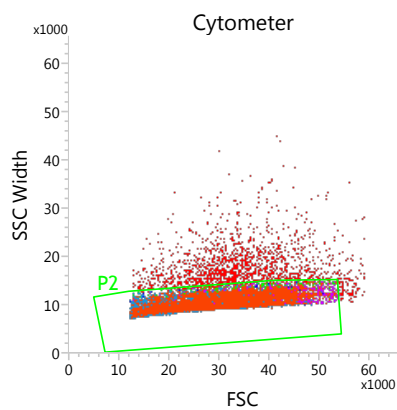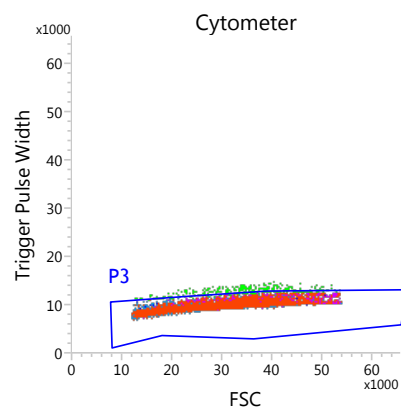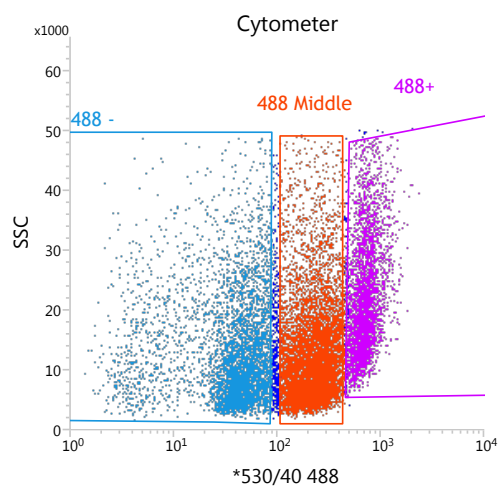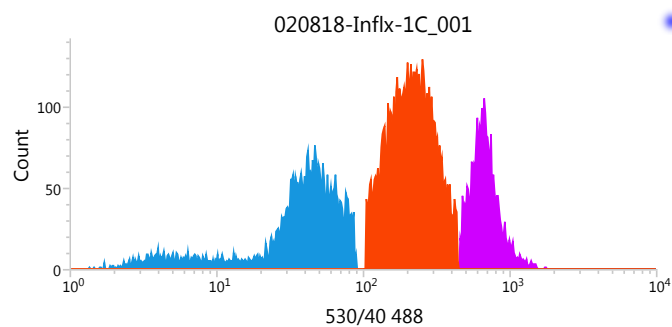

Populations: Cytometer

| Populations | Events | % Total | % Parent |
|-------------|--------|---------|----------|
| All Events  | 41,988 | 100.00% | ####     |
| P1          | 18,601 | 44.30%  | 44.30%   |
| P2          | 17,053 | 40.61%  | 91.68%   |
| P3          | 16,699 | 39.77%  | 97.92%   |
| 488+        | 3,453  | 8.22%   | 20.68%   |
| 488 -       | 4,817  | 11.47%  | 28.85%   |
| 488 Middle  | 7,803  | 18.58%  | 46.73%   |

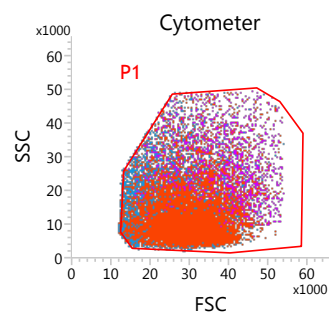

Supplement: Supplementary file 4 — This Tar/Gzip compressed file contains FANS images and gating strategies of the nuclei preparation experiments in this study. [file 41586_2020_3182_MOESM4_ESM.tgz › FANS_images/1C/CEMBA180208-1C.pdf]

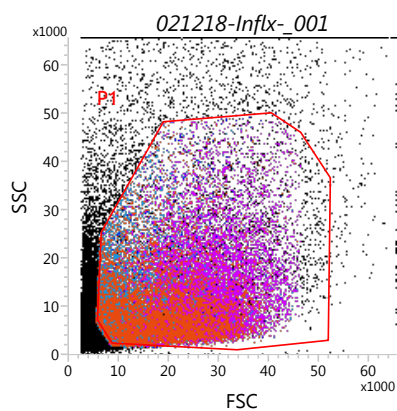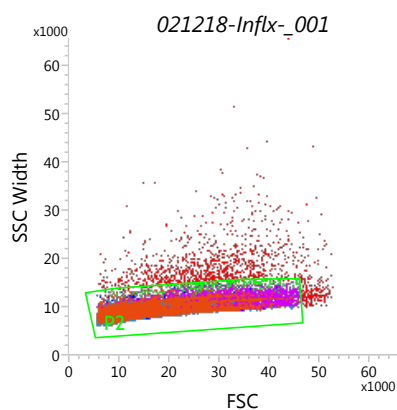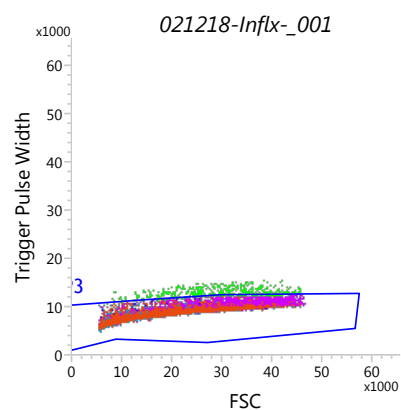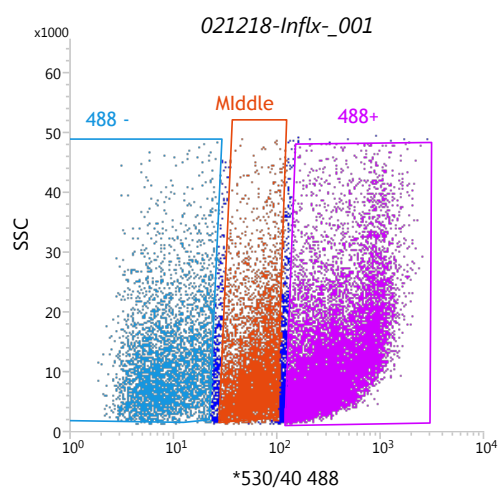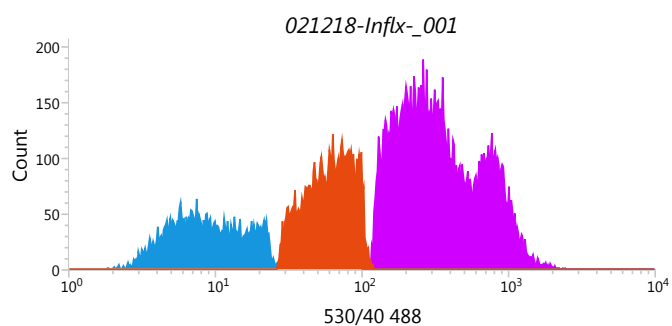

Populations: 021218-Inflx-\_001

| Populations | Events | % Total | % Parent |
|-------------|--------|---------|----------|
| All Events  | 41,321 | 100.00% | ####     |
| P1          | 27,509 | 66.57%  | 66.57%   |
| P2          | 26,225 | 63.47%  | 95.33%   |
| P3          | 25,668 | 62.12%  | 97.88%   |
| 488+        | 14,165 | 34.28%  | 55.19%   |
| 488 -       | 4,226  | 10.23%  | 16.46%   |
| Middle      | 5,913  | 14.31%  | 23.04%   |

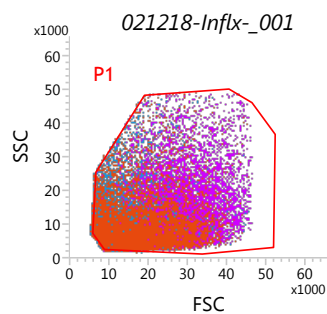

Supplement: Supplementary file 4 — This Tar/Gzip compressed file contains FANS images and gating strategies of the nuclei preparation experiments in this study. [file 41586_2020_3182_MOESM4_ESM.tgz › FANS_images/1C/CEMBA180212-1C.pdf]

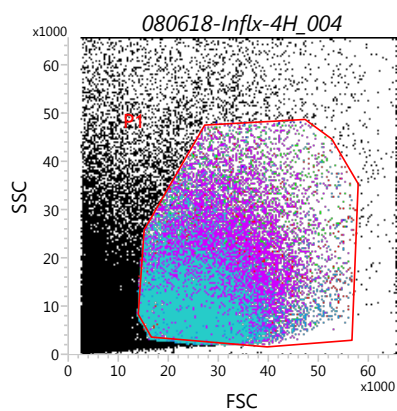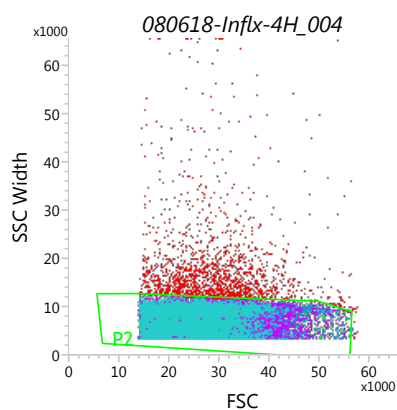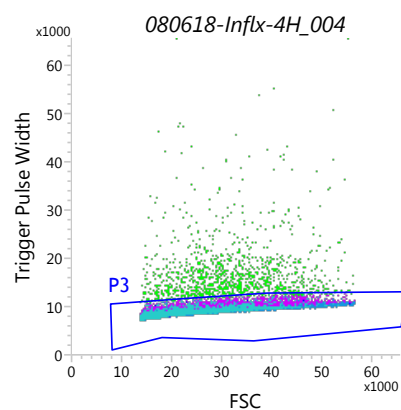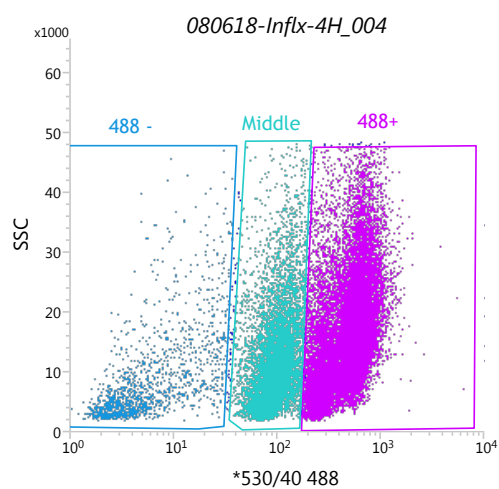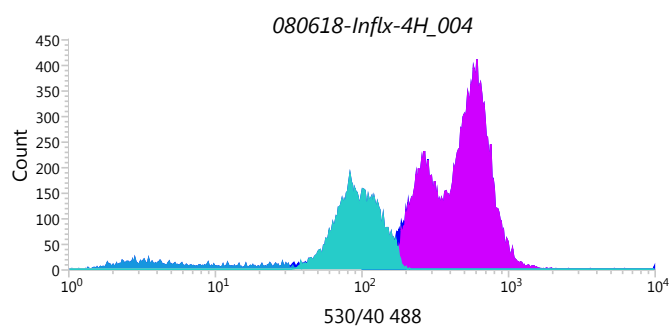

Populations: 080618-Inflx-4H\_004

| Populations | Events | % Total | % Parent |
|-------------|--------|---------|----------|
| All Events  | 73,900 | 100.00% | ####     |
| P1          | 31,163 | 42.17%  | 42.17%   |
| P2          | 29,281 | 39.62%  | 93.96%   |
| P3          | 28,067 | 37.98%  | 95.85%   |
| 488+        | 18,809 | 25.45%  | 67.01%   |
| 488 -       | 1,401  | 1.90%   | 4.99%    |
| Middle      | 7,731  | 10.46%  | 27.54%   |

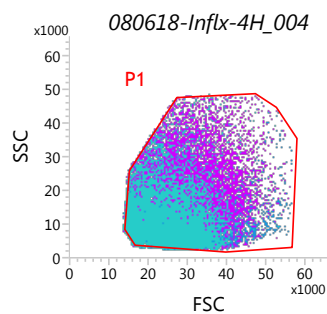

Supplement: Supplementary file 4 — This Tar/Gzip compressed file contains FANS images and gating strategies of the nuclei preparation experiments in this study. [file 41586_2020_3182_MOESM4_ESM.tgz › FANS_images/4H/CEMBA180806-4H.pdf]

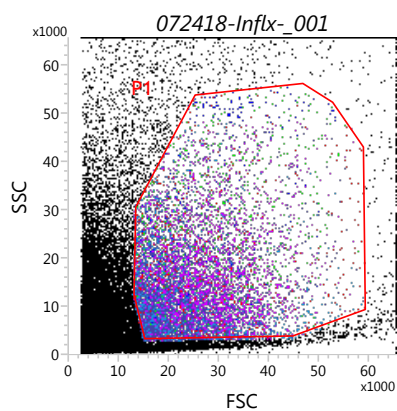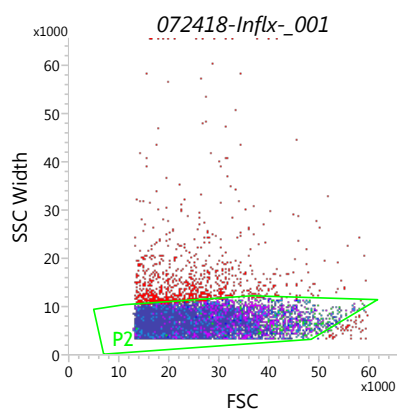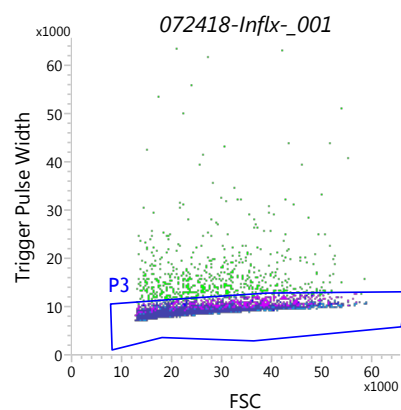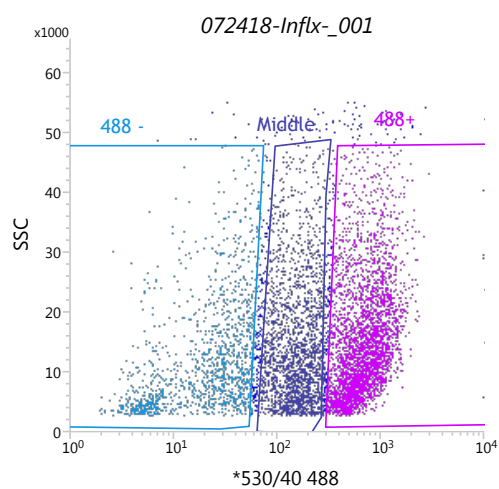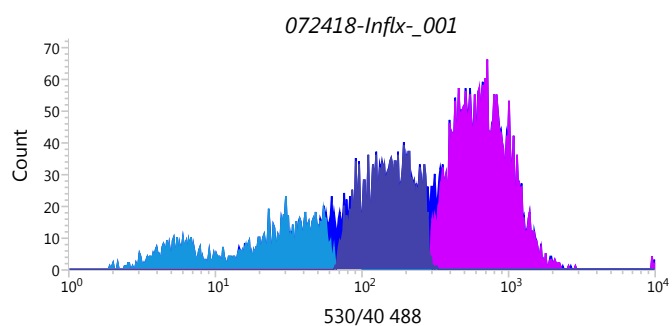

Populations: 072418-Inflx-\_001

| Populations | Events | % Total | % Parent |
|-------------|--------|---------|----------|
| All Events  | 38,499 | 100.00% | ####     |
| P1          | 8,853  | 23.00%  | 23.00%   |
| P2          | 7,786  | 20.22%  | 87.95%   |
| P3          | 7,111  | 18.47%  | 91.33%   |
| 488+        | 3,394  | 8.82%   | 47.73%   |
| 488 -       | 1,334  | 3.47%   | 18.76%   |
| Middle      | 2,027  | 5.27%   | 28.51%   |

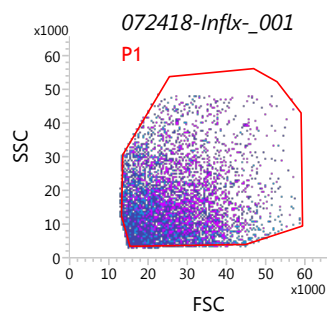

Supplement: Supplementary file 4 — This Tar/Gzip compressed file contains FANS images and gating strategies of the nuclei preparation experiments in this study. [file 41586_2020_3182_MOESM4_ESM.tgz › FANS_images/4H/CEMBA180724-4H.pdf]

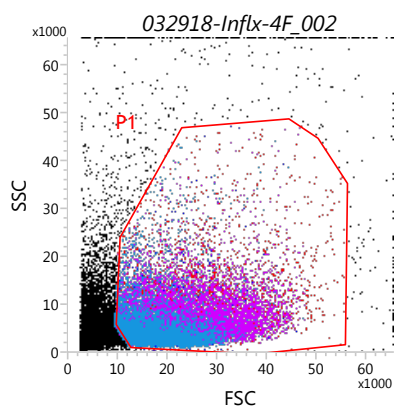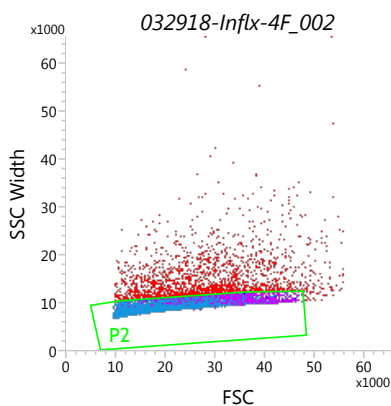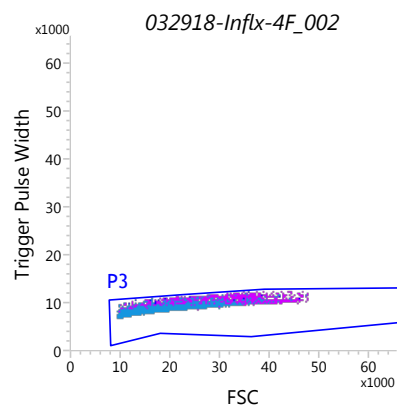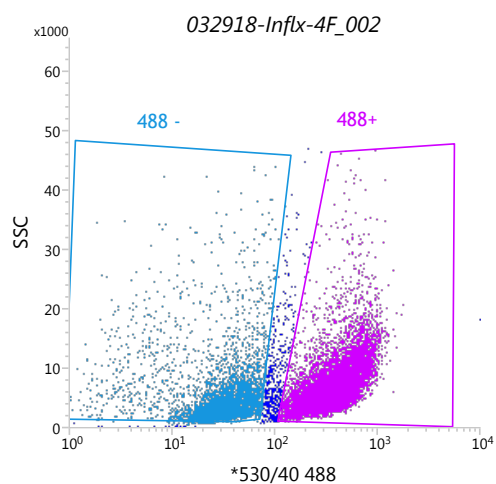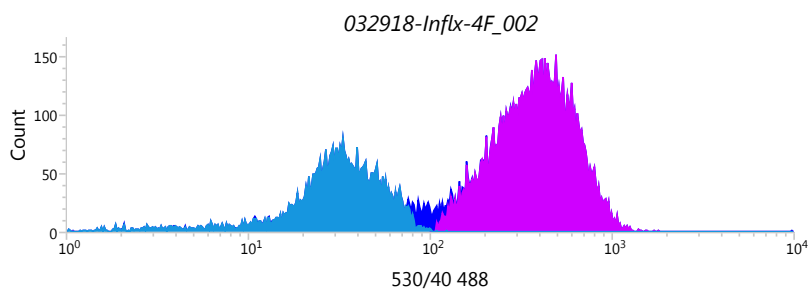

Populations: 032918-Inflx-4F\_002

| Populations | Events | % Total | % Parent |
|-------------|--------|---------|----------|
| All Events  | 26,104 | 100.00% | ####     |
| P1          | 16,345 | 62.61%  | 62.61%   |
| P2          | 14,477 | 55.46%  | 88.57%   |
| P3          | 14,477 | 55.46%  | 100.00%  |
| 488+        | 9,444  | 36.18%  | 65.23%   |
| 488-        | 4,576  | 17.53%  | 31.61%   |

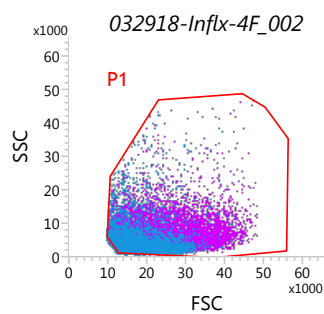

Supplement: Supplementary file 4 — This Tar/Gzip compressed file contains FANS images and gating strategies of the nuclei preparation experiments in this study. [file 41586_2020_3182_MOESM4_ESM.tgz › FANS_images/4F/CEMBA180329-4F.pdf]

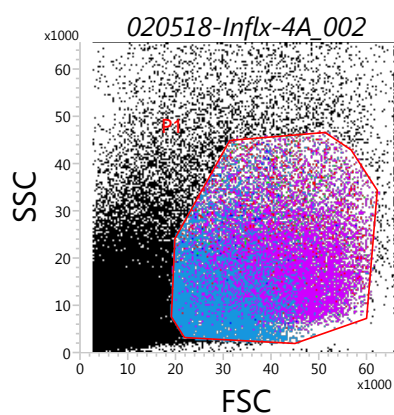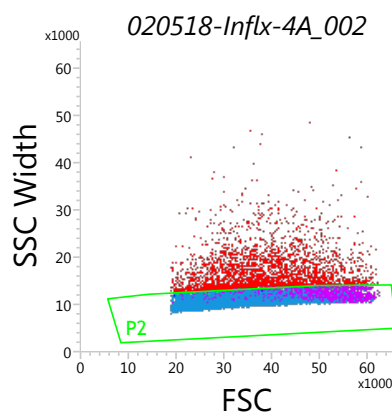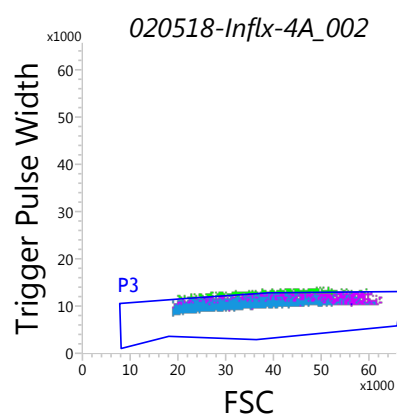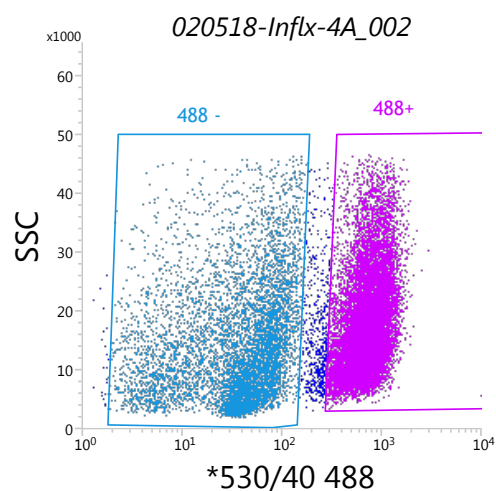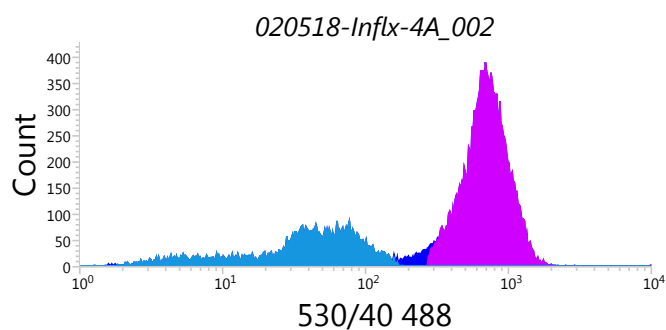

Populations: 020518-Inflx-4A\_002

| Populations | Events  | % Total | % Parent |
|-------------|---------|---------|----------|
| All Events  | 100,000 | 100.00% | ####     |
| P1          | 27,829  | 27.83%  | 27.83%   |
| P2          | 24,911  | 24.91%  | 89.51%   |
| P3          | 24,480  | 24.48%  | 98.27%   |
| 488+        | 16,645  | 16.64%  | 67.99%   |
| 488 -       | 7,191   | 7.19%   | 29.38%   |

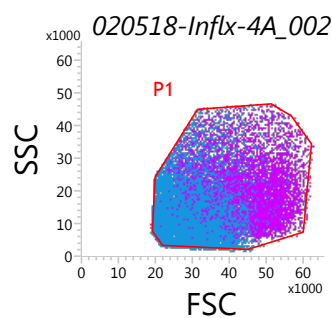

Supplement: Supplementary file 4 — This Tar/Gzip compressed file contains FANS images and gating strategies of the nuclei preparation experiments in this study. [file 41586_2020_3182_MOESM4_ESM.tgz › FANS_images/4A/CEMBA180205-4A.pdf]

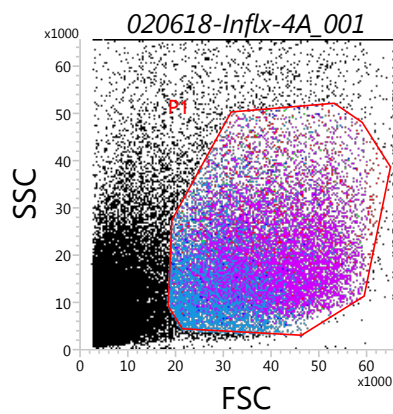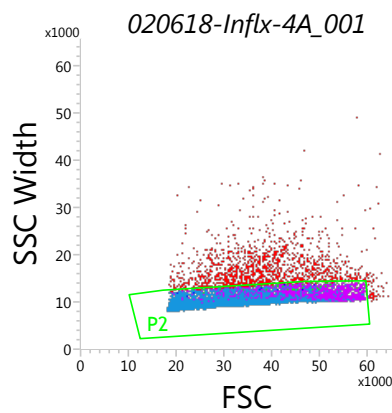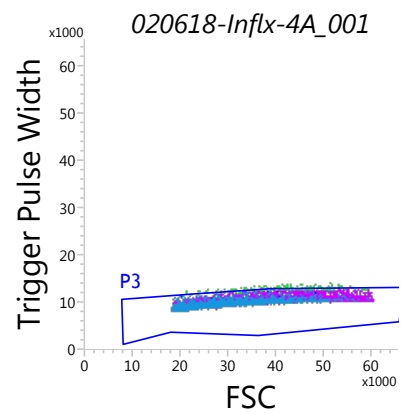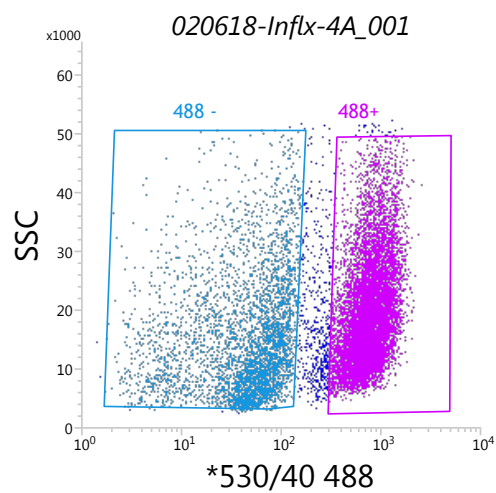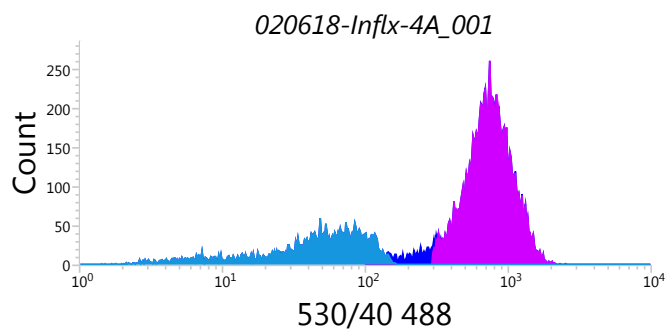

Populations: 020618-Inflx-4A\_001

| Populations | Events | % Total | % Parent |
|-------------|--------|---------|----------|
| All Events  | 56,462 | 100.00% | ####     |
| P1          | 16,585 | 29.37%  | 29.37%   |
| P2          | 15,082 | 26.71%  | 90.94%   |
| P3          | 14,873 | 26.34%  | 98.61%   |
| 488+        | 10,293 | 18.23%  | 69.21%   |
| 488 -       | 4,030  | 7.14%   | 27.10%   |

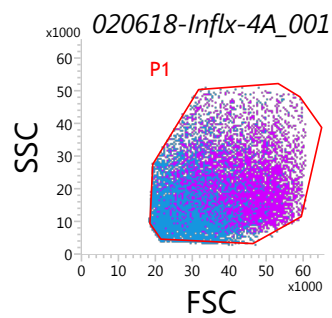

Supplement: Supplementary file 4 — This Tar/Gzip compressed file contains FANS images and gating strategies of the nuclei preparation experiments in this study. [file 41586_2020_3182_MOESM4_ESM.tgz › FANS_images/4A/CEMBA180206-4A.pdf]

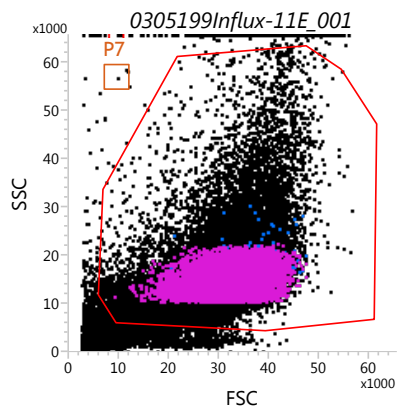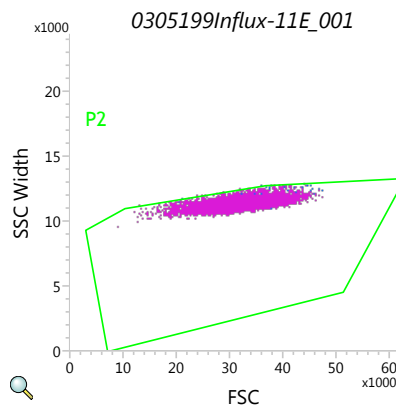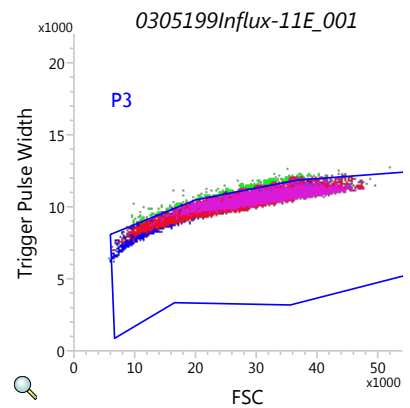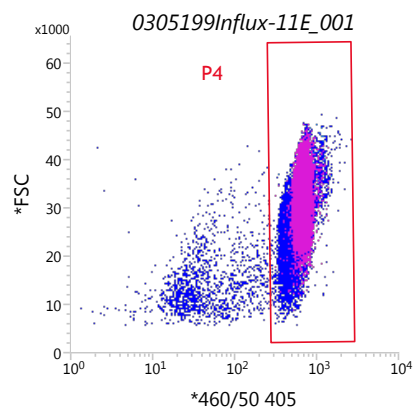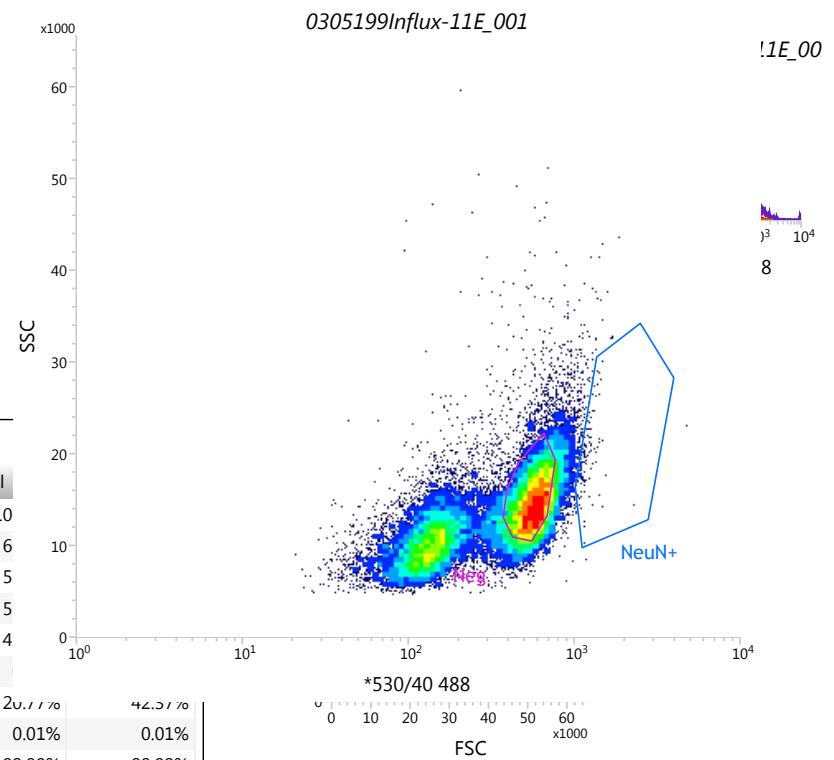

Populations: 0305199Influx-11E\_001

| Populations | Events | % Total |        |
|-------------|--------|---------|--------|
| All Events  | 50,000 | 10      |        |
| P1          | 34,151 | 6       |        |
| P2          | 26,267 | 5       |        |
| P3          | 25,913 | 5       |        |
| P4          | 24,509 | 4       |        |
| NeuN+       | 118    |         |        |
| Neg         | 10,385 | 20.11%  | 42.51% |
| P7          | 3      | 0.01%   | 0.01%  |
| NOT(P7)     | 49,997 | 99.99%  | 99.99% |

Supplement: Supplementary file 4 — This Tar/Gzip compressed file contains FANS images and gating strategies of the nuclei preparation experiments in this study. [file 41586_2020_3182_MOESM4_ESM.tgz › FANS_images/11E/CEMBA190305-11E.pdf]

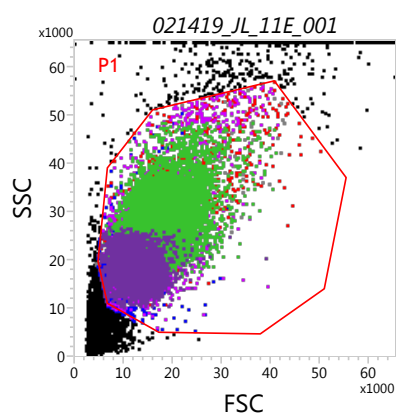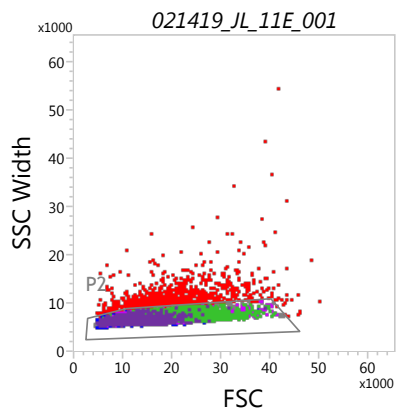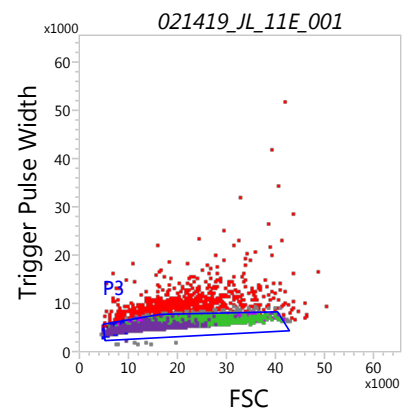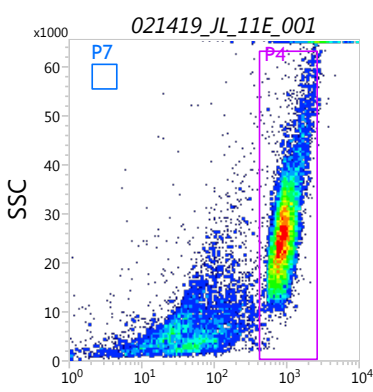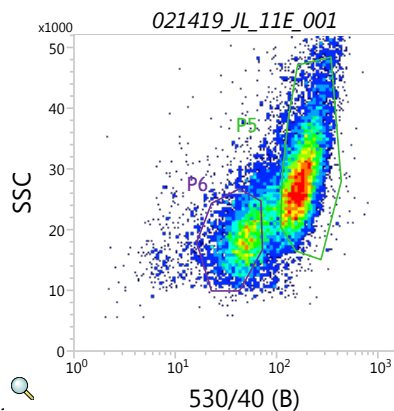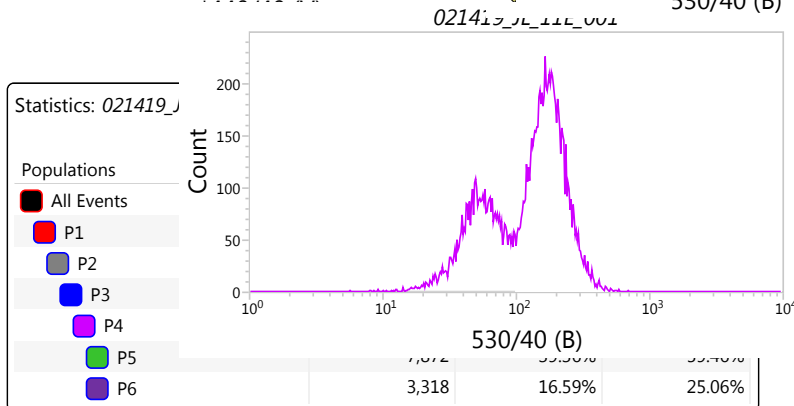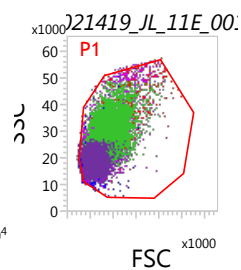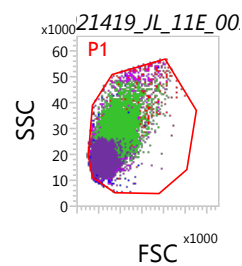

Supplement: Supplementary file 4 — This Tar/Gzip compressed file contains FANS images and gating strategies of the nuclei preparation experiments in this study. [file 41586_2020_3182_MOESM4_ESM.tgz › FANS_images/11E/CEMBA190214-11E.pdf]

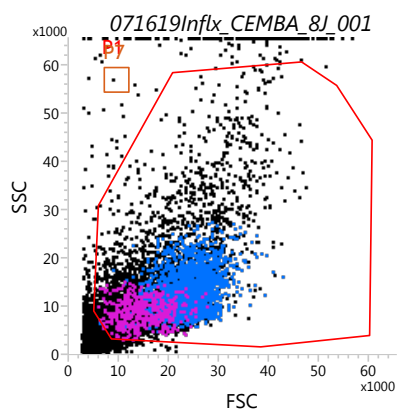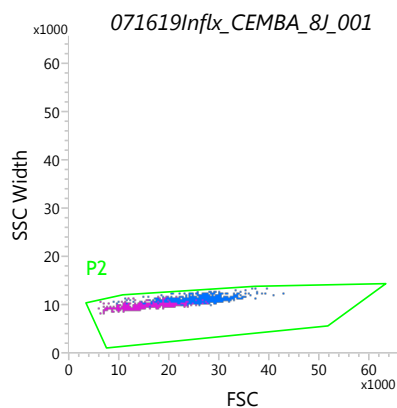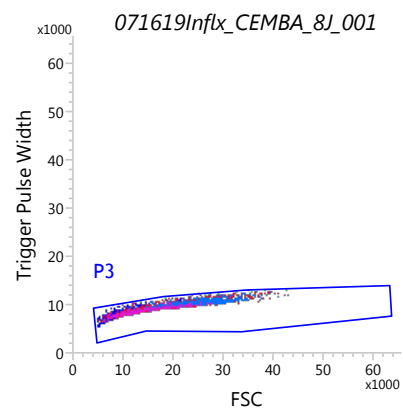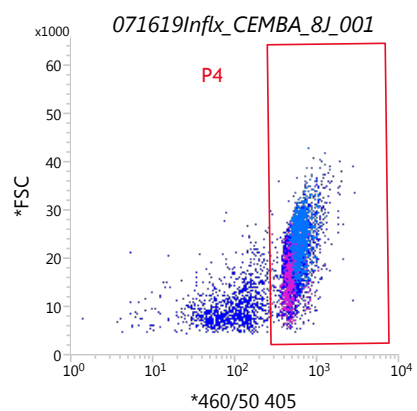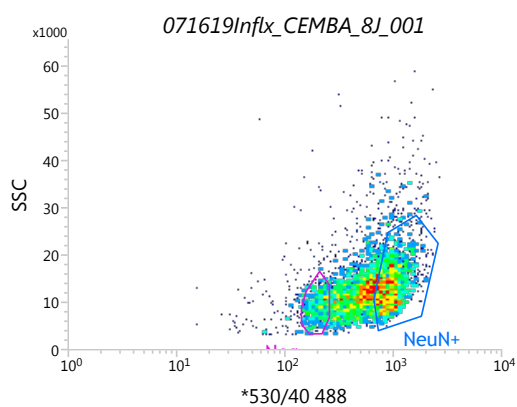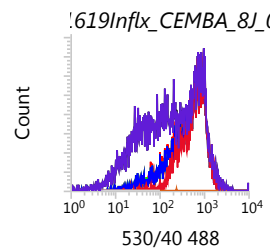

Populations: 071619Inflx\_CEMBA\_8J\_001

| Populations | Events | % Total | % Parent |
|-------------|--------|---------|----------|
| All Events  | 10,404 | 100.00% | ####     |
| P1          | 5,915  | 56.85%  | 56.85%   |
| P2          | 5,322  | 51.15%  | 89.97%   |
| P3          | 5,307  | 51.01%  | 99.72%   |
| P4          | 4,223  | 40.59%  | 79.57%   |
| NeuN+       | 1,666  | 16.01%  | 39.45%   |
| Neg         | 426    | 4.09%   | 10.09%   |
| P7          | 1      | 0.01%   | 0.01%    |
| NOT(P7)     | 10,403 | 99.99%  | 99.99%   |

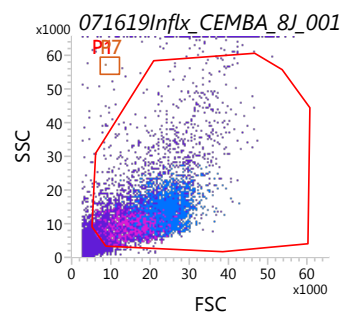

Supplement: Supplementary file 4 — This Tar/Gzip compressed file contains FANS images and gating strategies of the nuclei preparation experiments in this study. [file 41586_2020_3182_MOESM4_ESM.tgz › FANS_images/8J/CEMBA190716-8J.pdf]

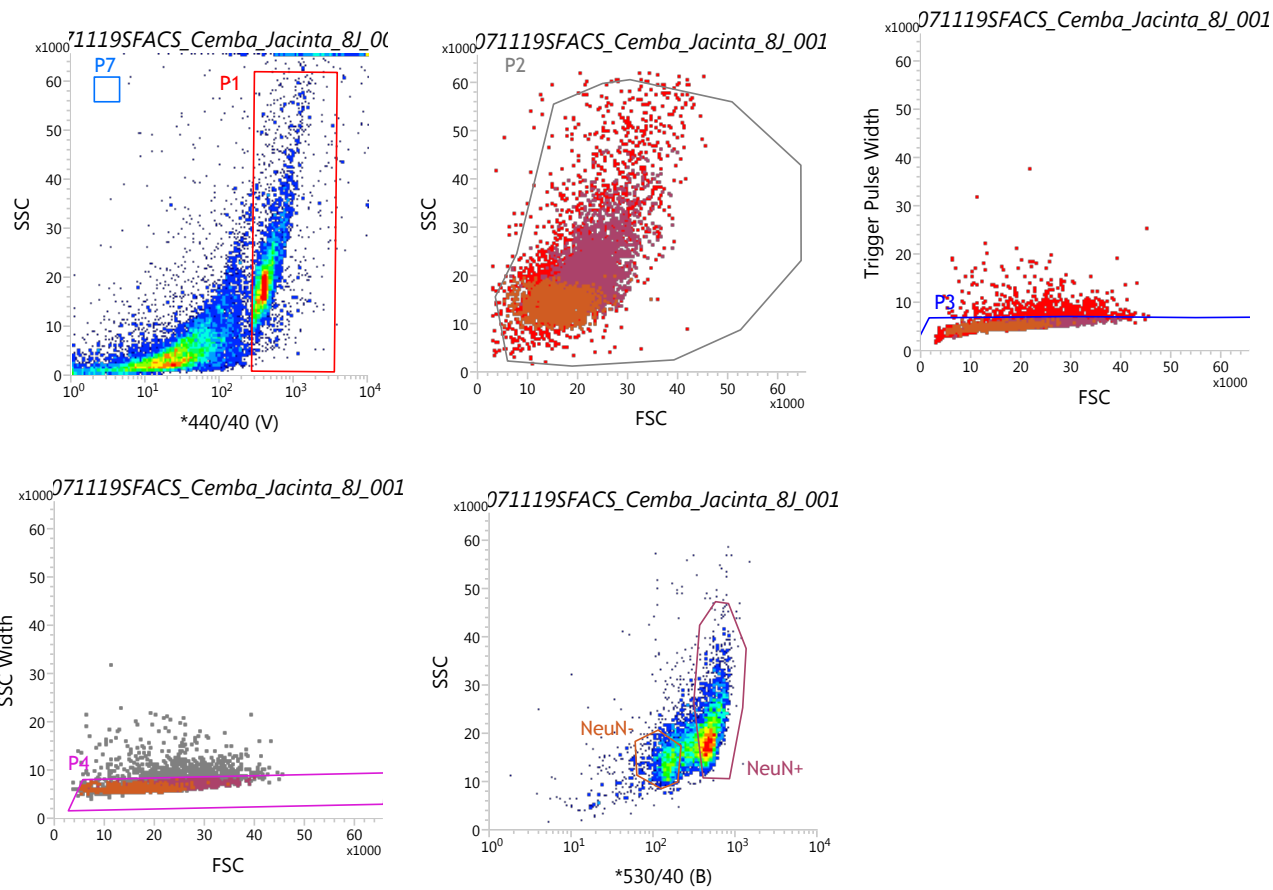

Statistics: 071119SFACS\_Cemba\_Jacinta\_8J\_001

| Populations | Events | % Total | % Parent |
|-------------|--------|---------|----------|
| All Events  | 20,000 | 100.00% | ####     |
| P1          | 7,352  | 36.76%  | 36.76%   |
| P2          | 7,290  | 36.45%  | 99.16%   |
| P3          | 6,586  | 32.93%  | 90.34%   |
| P4          | 6,483  | 32.42%  | 98.44%   |
| NeuN+       | 3,786  | 18.93%  | 58.40%   |
| NeuN-       | 1,169  | 5.85%   | 18.03%   |

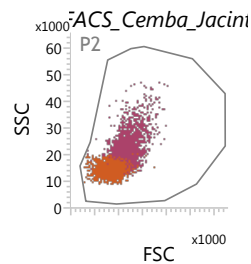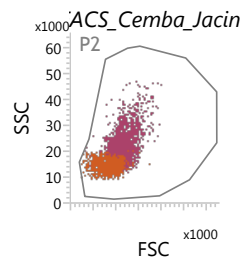

Supplement: Supplementary file 4 — This Tar/Gzip compressed file contains FANS images and gating strategies of the nuclei preparation experiments in this study. [file 41586_2020_3182_MOESM4_ESM.tgz › FANS_images/8J/CEMBA190711-8J.pdf]

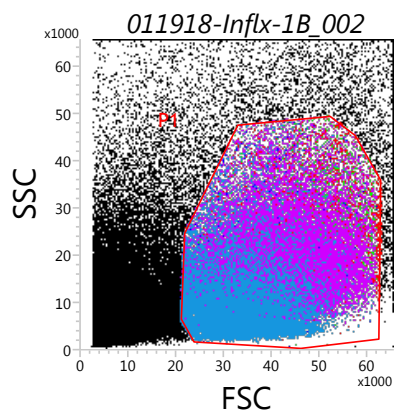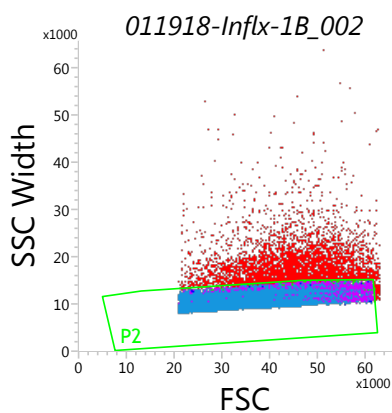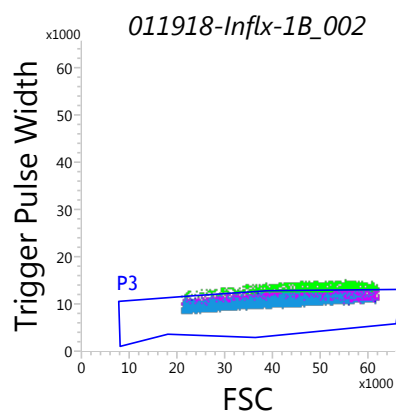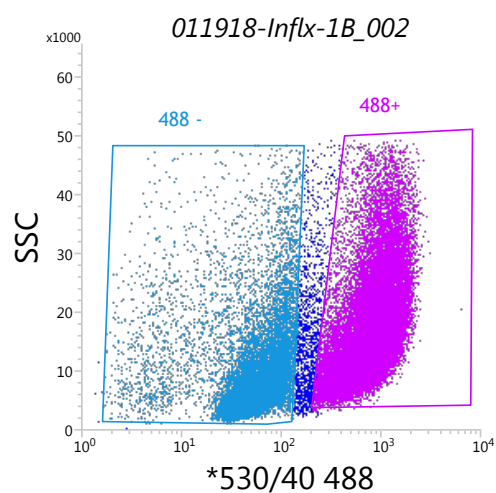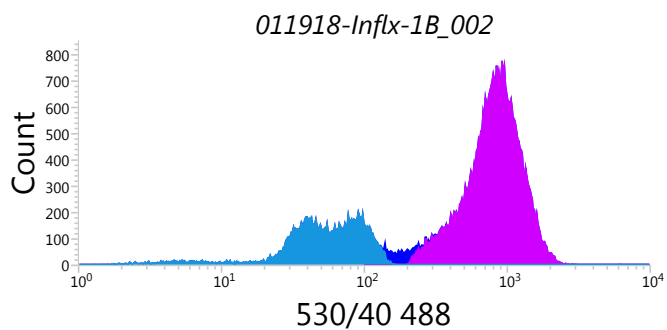

Populations: 011918-Inflx-1B\_002

| Populations | Events  | % Total | % Parent |
|-------------|---------|---------|----------|
| All Events  | 146,481 | 100.00% | ####     |
| P1          | 60,892  | 41.57%  | 41.57%   |
| P2          | 56,318  | 38.45%  | 92.49%   |
| P3          | 54,626  | 37.29%  | 97.00%   |
| 488+        | 39,228  | 26.78%  | 71.81%   |
| 488 -       | 13,997  | 9.56%   | 25.62%   |

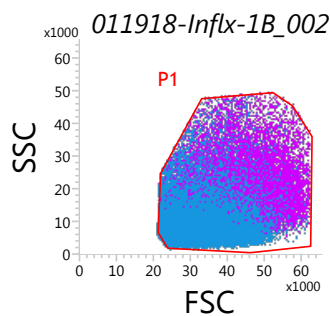

Supplement: Supplementary file 4 — This Tar/Gzip compressed file contains FANS images and gating strategies of the nuclei preparation experiments in this study. [file 41586_2020_3182_MOESM4_ESM.tgz › FANS_images/1B/CEMBA180119-1B.pdf]

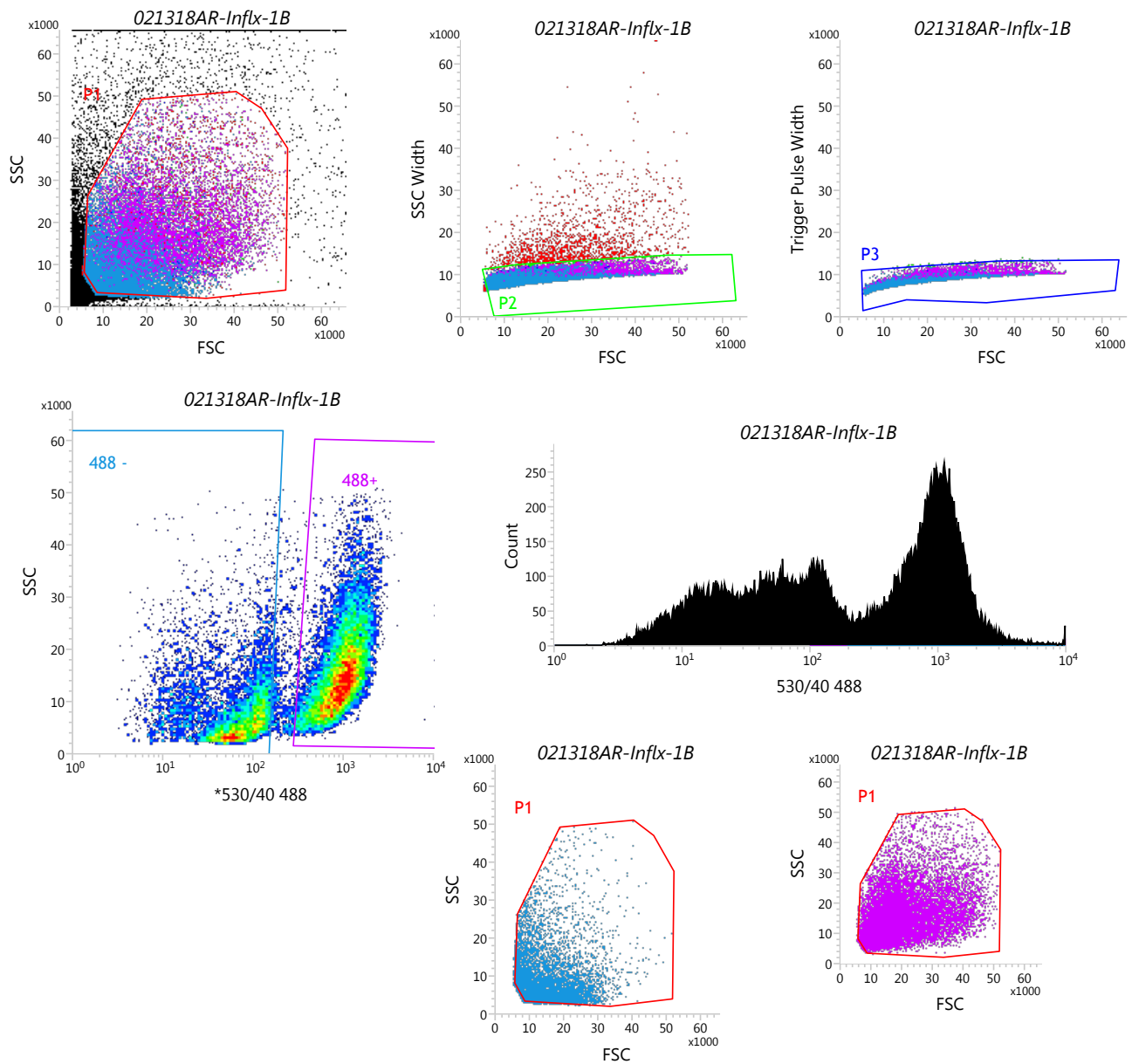

| Populations: 021318AR-Inflx-1B |        |         |          |
|--------------------------------|--------|---------|----------|
| Populations                    | Events | % Total | % Parent |
| All Events                     | 31,933 | 100.00% | ####     |
| P1                             | 21,819 | 68.33%  | 68.33%   |
| P2                             | 20,087 | 62.90%  | 92.06%   |
| P3                             | 20,000 | 62.63%  | 99.57%   |
| 488+                           | 12,554 | 39.31%  | 62.77%   |
| 488 -                          | 6,778  | 21.23%  | 33.89%   |

Supplement: Supplementary file 4 — This Tar/Gzip compressed file contains FANS images and gating strategies of the nuclei preparation experiments in this study. [file 41586_2020_3182_MOESM4_ESM.tgz › FANS_images/1B/CEMBA180213-1B.pdf]

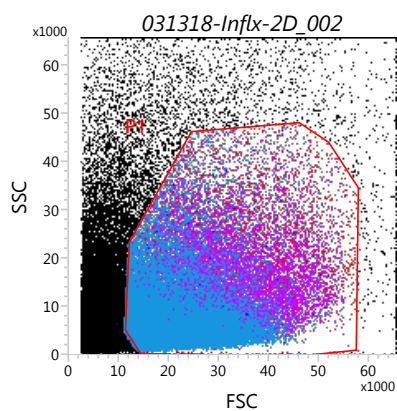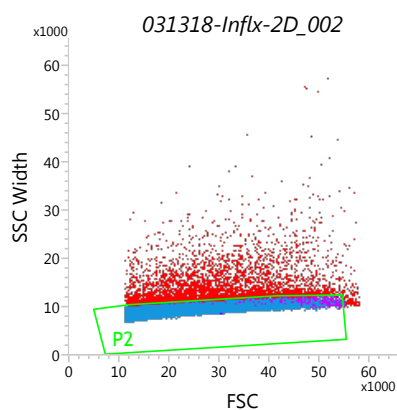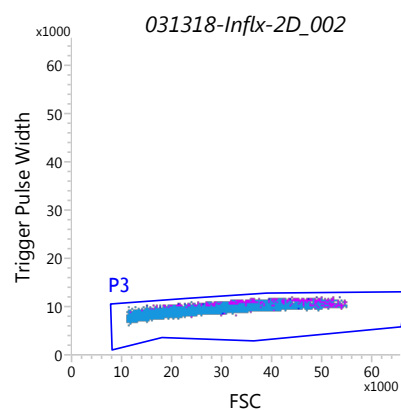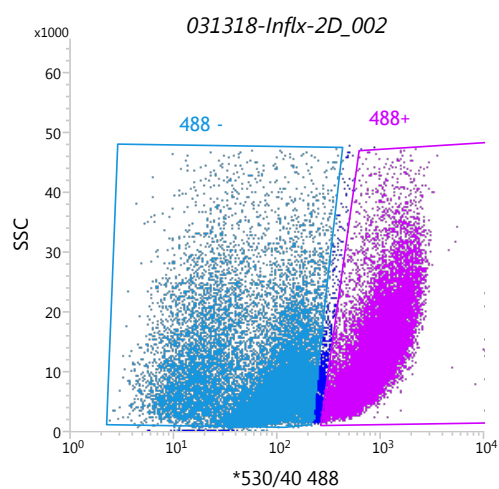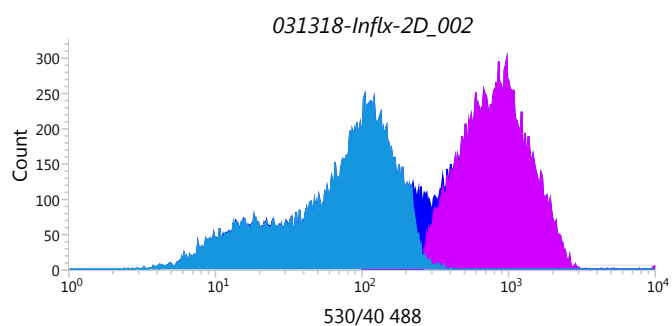

Populations: 031318-Inflx-2D\_002

| Populations | Events  | % Total | % Parent |
|-------------|---------|---------|----------|
| All Events  | 109,791 | 100.00% | ####     |
| P1          | 46,144  | 42.03%  | 42.03%   |
| P2          | 41,153  | 37.48%  | 89.18%   |
| P3          | 41,153  | 37.48%  | 100.00%  |
| 488+        | 19,649  | 17.90%  | 47.75%   |
| 488 -       | 20,398  | 18.58%  | 49.57%   |

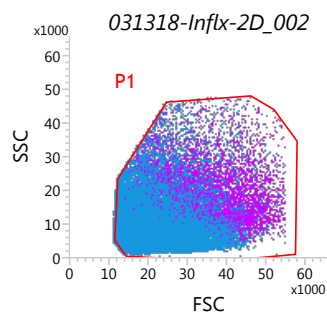

Supplement: Supplementary file 4 — This Tar/Gzip compressed file contains FANS images and gating strategies of the nuclei preparation experiments in this study. [file 41586_2020_3182_MOESM4_ESM.tgz › FANS_images/2D/CEMBA180313-2D.pdf]

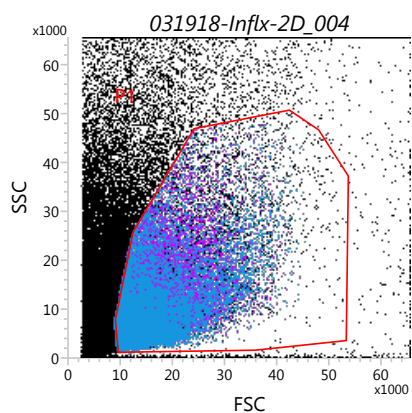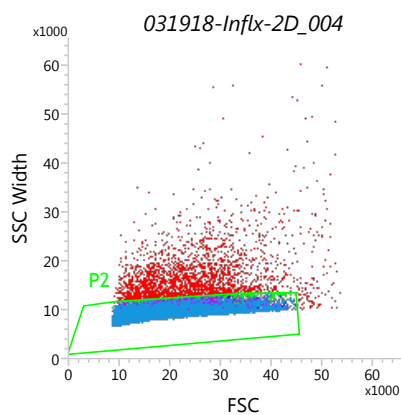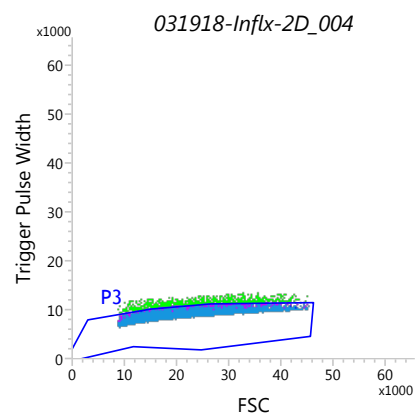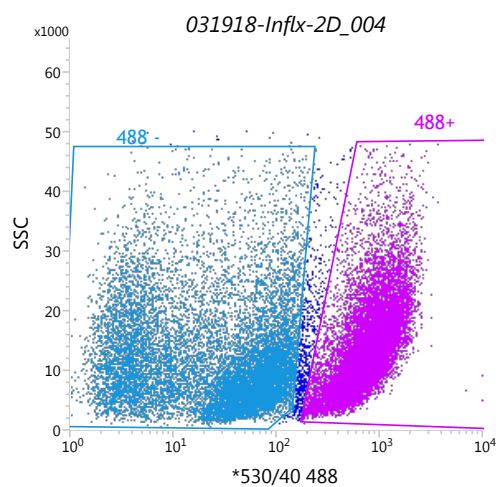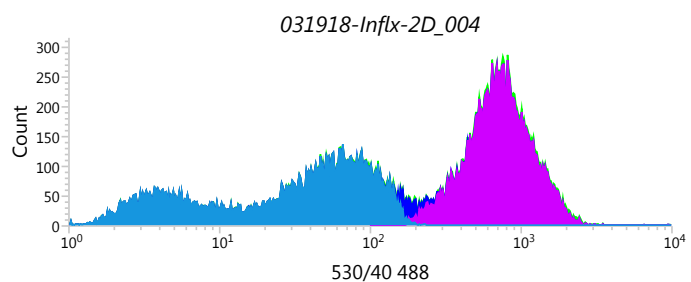

Populations: 031918-Inflx-2D\_004

| Populations | Events | % Total | % Parent |
|-------------|--------|---------|----------|
| All Events  | 81,562 | 100.00% | ####     |
| P1          | 34,734 | 42.59%  | 42.59%   |
| P2          | 32,197 | 39.48%  | 92.70%   |
| P3          | 31,343 | 38.43%  | 97.35%   |
| 488+        | 16,033 | 19.66%  | 51.15%   |
| 488 -       | 14,667 | 17.98%  | 46.80%   |

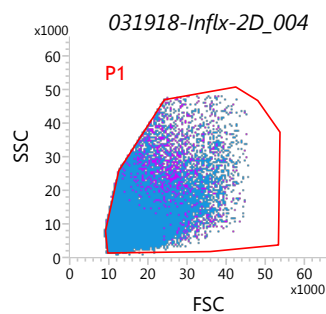

Supplement: Supplementary file 4 — This Tar/Gzip compressed file contains FANS images and gating strategies of the nuclei preparation experiments in this study. [file 41586_2020_3182_MOESM4_ESM.tgz › FANS_images/2D/CEMBA180319-2D.pdf]

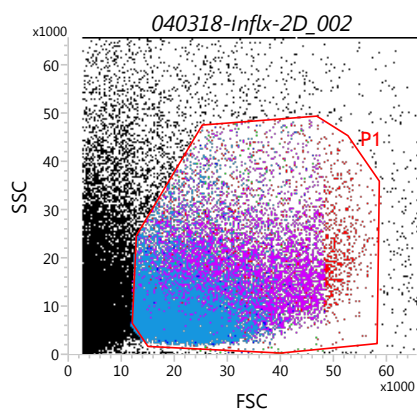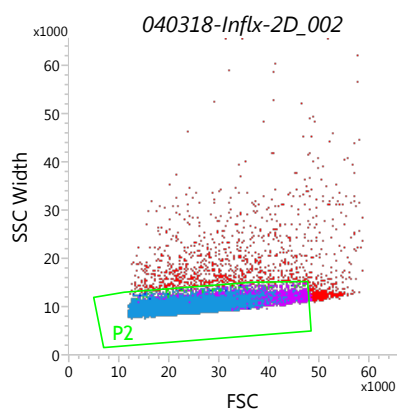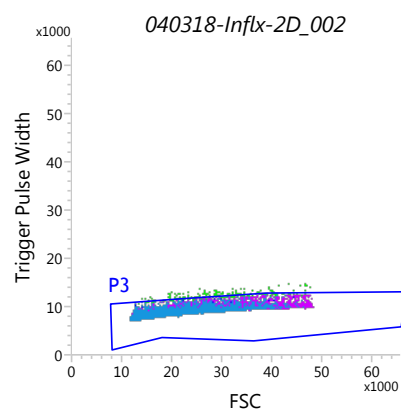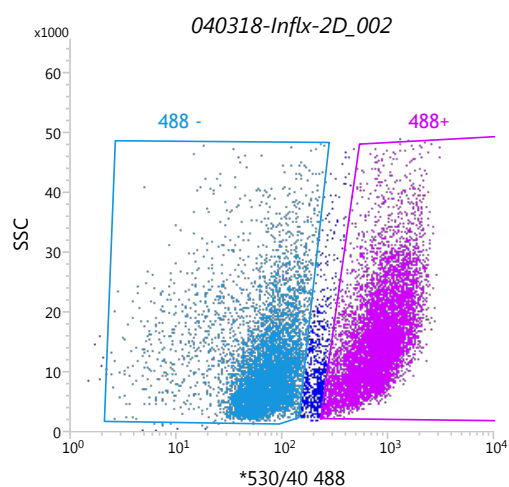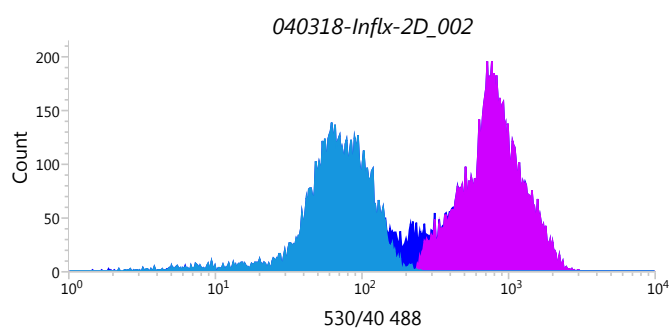

Populations: 040318-Inflx-2D\_002

| Populations | Events | % Total | % Parent |
|-------------|--------|---------|----------|
| All Events  | 44,994 | 100.00% | ####     |
| P1          | 20,254 | 45.01%  | 45.01%   |
| P2          | 18,693 | 41.55%  | 92.29%   |
| P3          | 18,499 | 41.11%  | 98.96%   |
| 488+        | 9,578  | 21.29%  | 51.78%   |
| 488 -       | 8,109  | 18.02%  | 43.83%   |

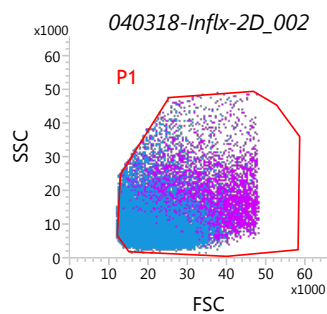

Supplement: Supplementary file 4 — This Tar/Gzip compressed file contains FANS images and gating strategies of the nuclei preparation experiments in this study. [file 41586_2020_3182_MOESM4_ESM.tgz › FANS_images/2D/CEMBA180403-2D.pdf]

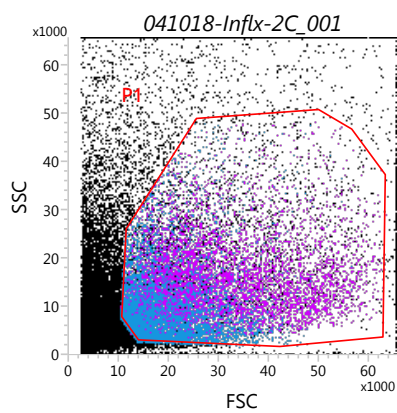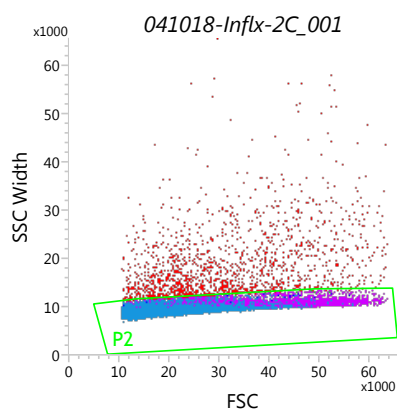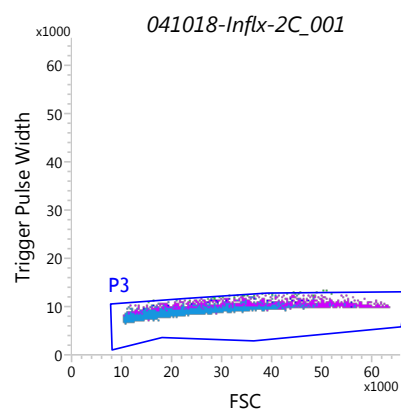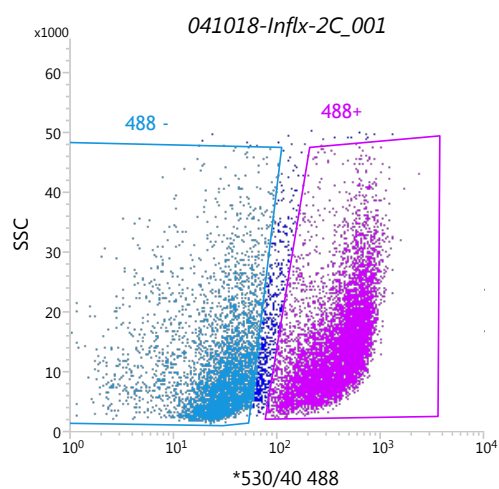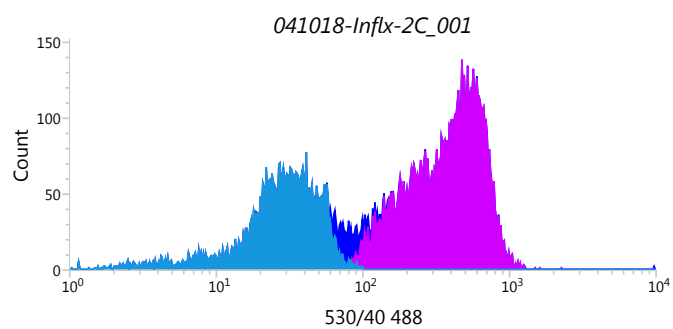

Populations: 041018-Inflx-2C\_001

| Populations | Events | % Total | % Parent |
|-------------|--------|---------|----------|
| All Events  | 38,657 | 100.00% | ####     |
| P1          | 15,430 | 39.92%  | 39.92%   |
| P2          | 13,767 | 35.61%  | 89.22%   |
| P3          | 13,735 | 35.53%  | 99.77%   |
| 488+        | 8,371  | 21.65%  | 60.95%   |
| 488 -       | 4,773  | 12.35%  | 34.75%   |

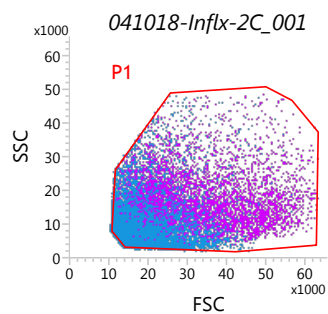

Supplement: Supplementary file 4 — This Tar/Gzip compressed file contains FANS images and gating strategies of the nuclei preparation experiments in this study. [file 41586_2020_3182_MOESM4_ESM.tgz › FANS_images/2C/CEMBA180410-2C.pdf]

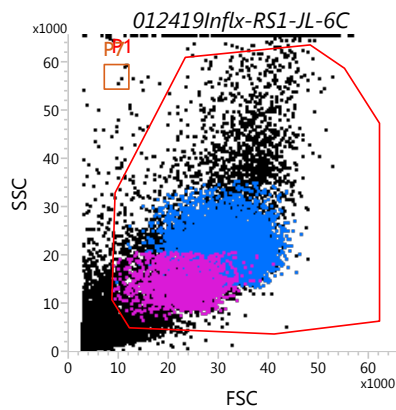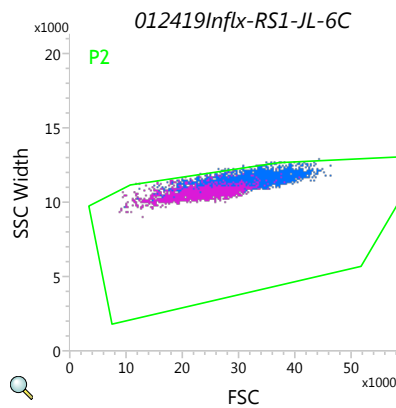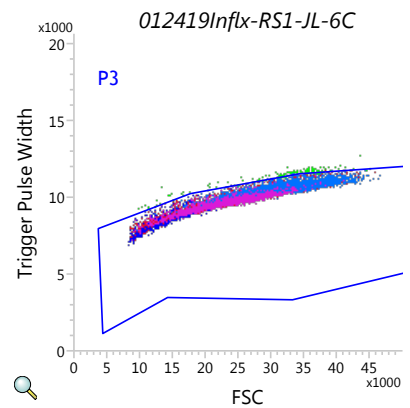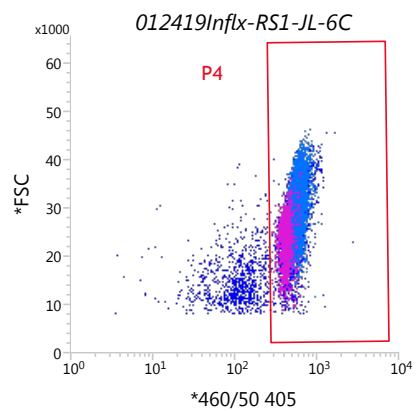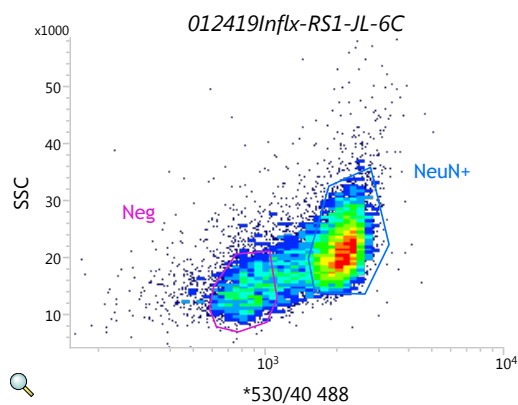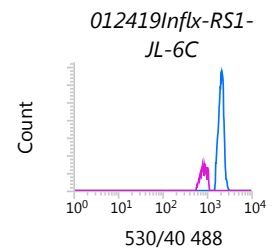

Populations: 012419Inflx-RS1-JL-6C

| Populations | Events | % Total | % Parent |
|-------------|--------|---------|----------|
| All Events  | 27,054 | 100.00% | ####     |
| P1          | 12,630 | 46.68%  | 46.68%   |
| P2          | 11,068 | 40.91%  | 87.63%   |
| P3          | 10,906 | 40.31%  | 98.54%   |
| P4          | 10,000 | 36.96%  | 91.69%   |
| NeuN+       | 6,350  | 23.47%  | 63.50%   |
| Neg         | 1,645  | 6.08%   | 16.45%   |
| P7          | 4      | 0.01%   | 0.01%    |
| NOT(P7)     | 27,050 | 99.99%  | 99.99%   |

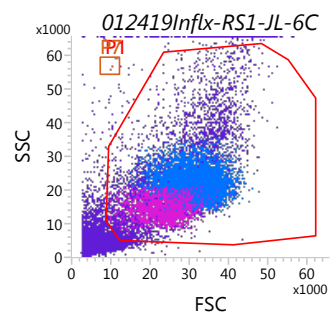

Supplement: Supplementary file 4 — This Tar/Gzip compressed file contains FANS images and gating strategies of the nuclei preparation experiments in this study. [file 41586_2020_3182_MOESM4_ESM.tgz › FANS_images/6C/CEMBA190124-6C.pdf]

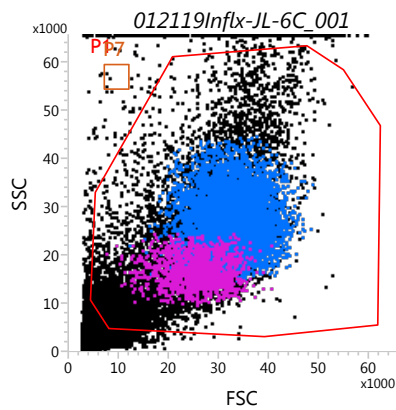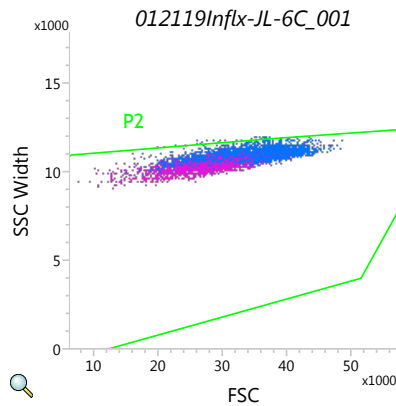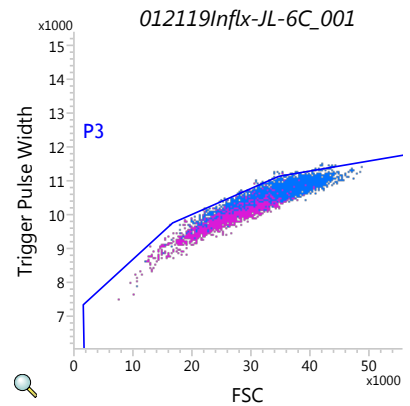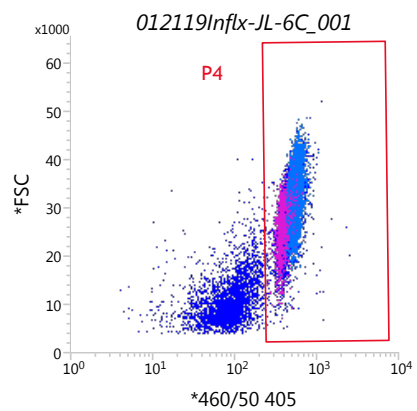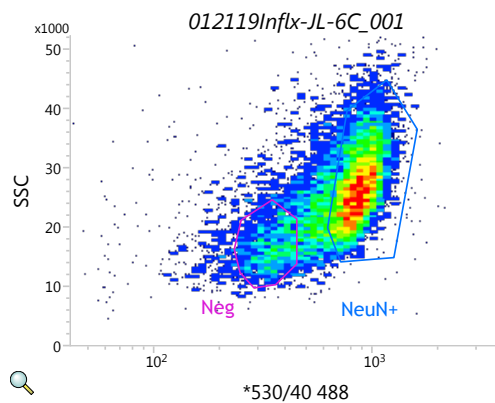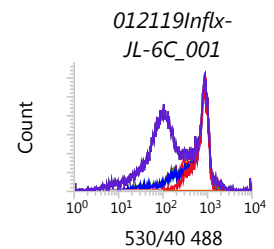

Populations: 012119Inflx-JL-6C\_001

| Populations | Events | % Total | % Parent |
|-------------|--------|---------|----------|
| All Events  | 32,863 | 100.00% | ####     |
| P1          | 15,075 | 45.87%  | 45.87%   |
| P2          | 13,425 | 40.85%  | 89.05%   |
| P3          | 12,951 | 39.41%  | 96.47%   |
| P4          | 9,999  | 30.43%  | 77.21%   |
| NeuN+       | 6,268  | 19.07%  | 62.69%   |
| Neg         | 1,152  | 3.51%   | 11.52%   |
| P7          | 3      | 0.01%   | 0.01%    |
| NOT(P7)     | 32,860 | 99.99%  | 99.99%   |

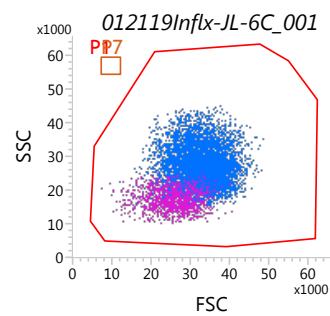

Supplement: Supplementary file 4 — This Tar/Gzip compressed file contains FANS images and gating strategies of the nuclei preparation experiments in this study. [file 41586_2020_3182_MOESM4_ESM.tgz › FANS_images/6C/CEMBA190121-6C.pdf]

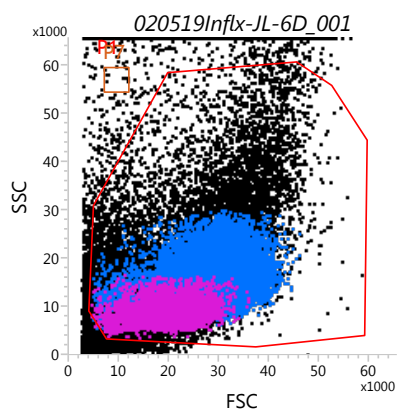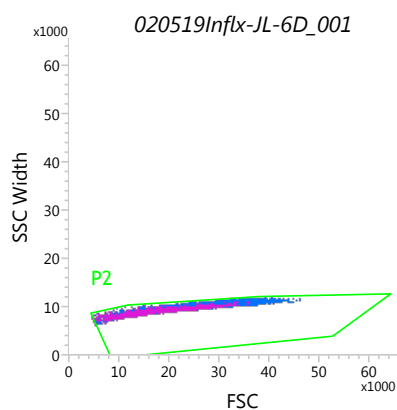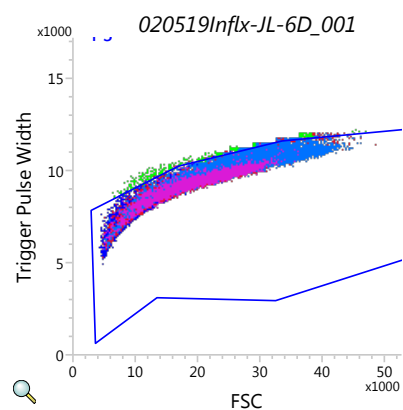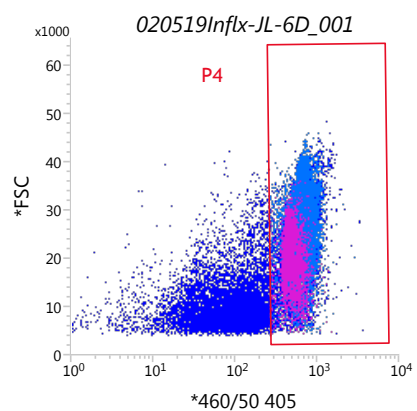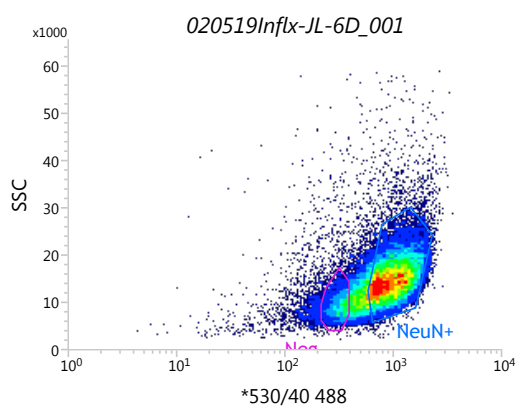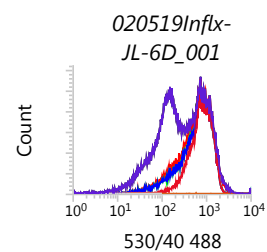

Populations: 020519Inflx-JL-6D\_001

| Populations | Events | % Total | % Parent |
|-------------|--------|---------|----------|
| All Events  | 84,039 | 100.00% | ####     |
| P1          | 49,695 | 59.13%  | 59.13%   |
| P2          | 42,790 | 50.92%  | 86.11%   |
| P3          | 42,416 | 50.47%  | 99.13%   |
| P4          | 33,900 | 40.34%  | 79.92%   |
| NeuN+       | 22,526 | 26.80%  | 66.45%   |
| Neg         | 3,214  | 3.82%   | 9.48%    |
| P7          | 11     | 0.01%   | 0.01%    |
| NOT(P7)     | 84,028 | 99.99%  | 99.99%   |

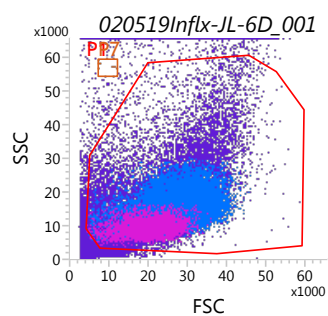

Supplement: Supplementary file 4 — This Tar/Gzip compressed file contains FANS images and gating strategies of the nuclei preparation experiments in this study. [file 41586_2020_3182_MOESM4_ESM.tgz › FANS_images/6D/CEMBA190205-6D.pdf]

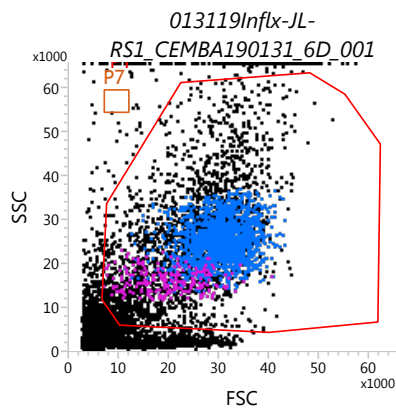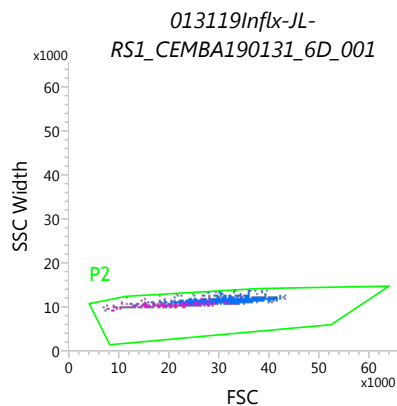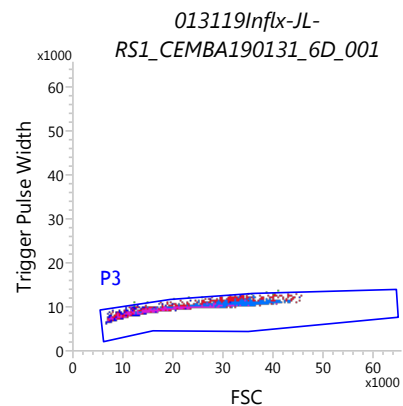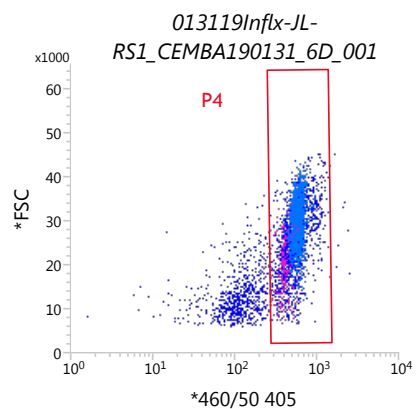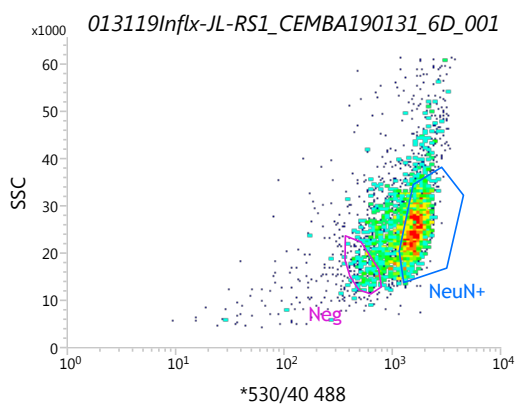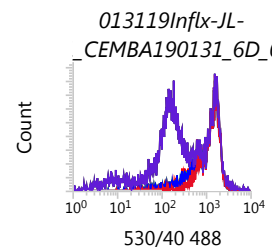

Populations: 013119Inflx-JL-RS1\_CEMBA190131\_6D\_001

| Populations | Events | % Total | % Parent |
|-------------|--------|---------|----------|
| All Events  | 9,632  | 100.00% | ####     |
| P1          | 4,202  | 43.63%  | 43.63%   |
| P2          | 3,980  | 41.32%  | 94.72%   |
| P3          | 3,969  | 41.21%  | 99.72%   |
| P4          | 3,301  | 34.27%  | 83.17%   |
| NeuN+       | 1,596  | 16.57%  | 48.35%   |
| Neg         | 221    | 2.29%   | 6.69%    |
| P7          | 0      | 0.00%   | 0.00%    |
| NOT(P7)     | 9,632  | 100.00% | 100.00%  |

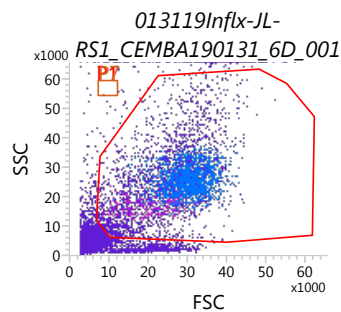

Supplement: Supplementary file 4 — This Tar/Gzip compressed file contains FANS images and gating strategies of the nuclei preparation experiments in this study. [file 41586_2020_3182_MOESM4_ESM.tgz › FANS_images/6D/CEMBA190131-6D.pdf]

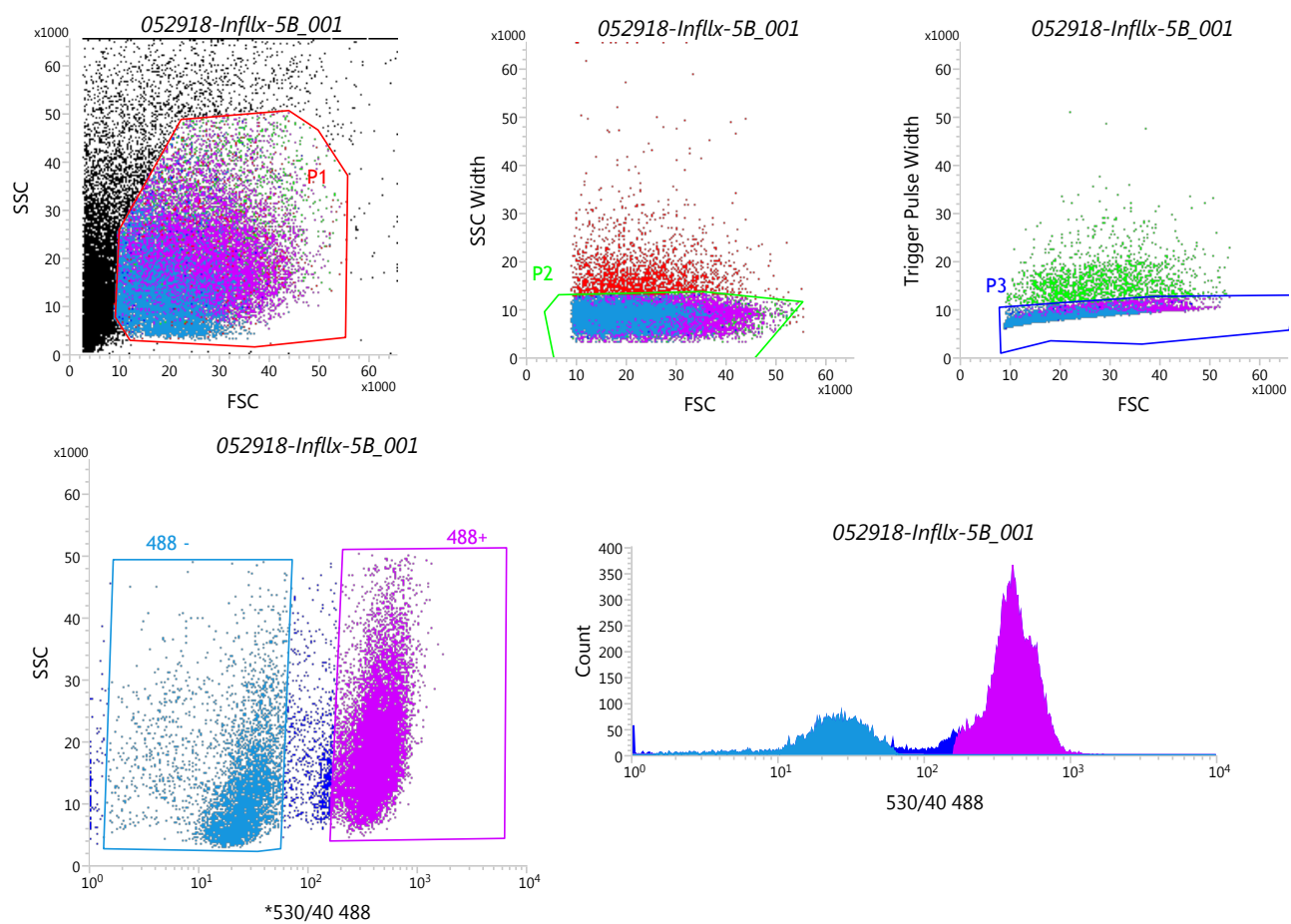

Populations: 052918-Inflx-5B\_001

| Populations | Events | % Total | % Parent |
|-------------|--------|---------|----------|
| All Events  | 38,208 | 100.00% | ####     |
| P1          | 23,323 | 61.04%  | 61.04%   |
| P2          | 21,255 | 55.63%  | 91.13%   |
| P3          | 19,494 | 51.02%  | 91.71%   |
| 488+        | 13,678 | 35.80%  | 70.17%   |
| 488 -       | 4,844  | 12.68%  | 24.85%   |

Supplement: Supplementary file 4 — This Tar/Gzip compressed file contains FANS images and gating strategies of the nuclei preparation experiments in this study. [file 41586_2020_3182_MOESM4_ESM.tgz › FANS_images/5B/CEMBA180529-5B.pdf]

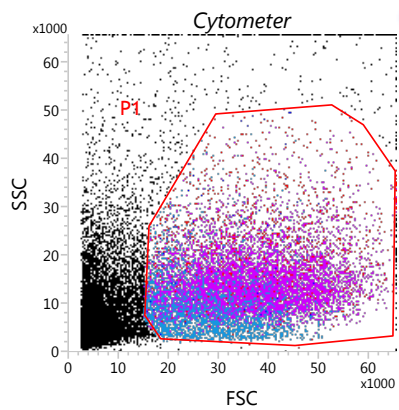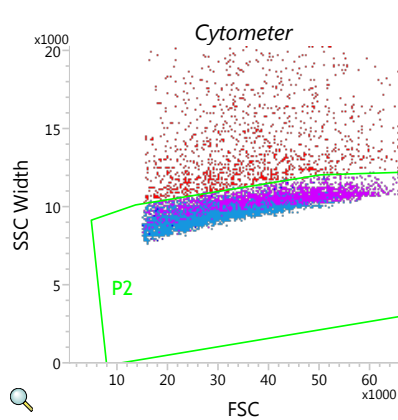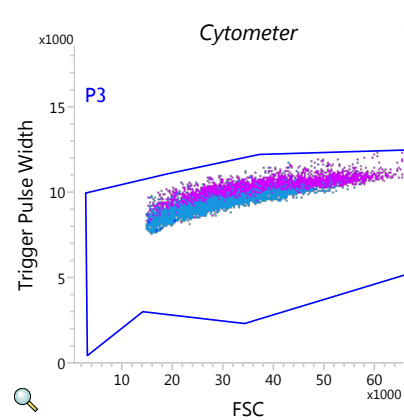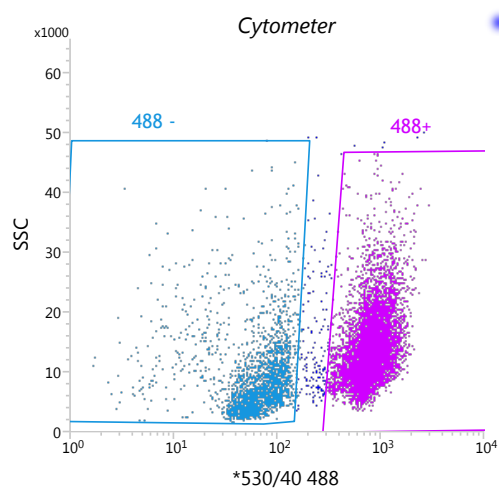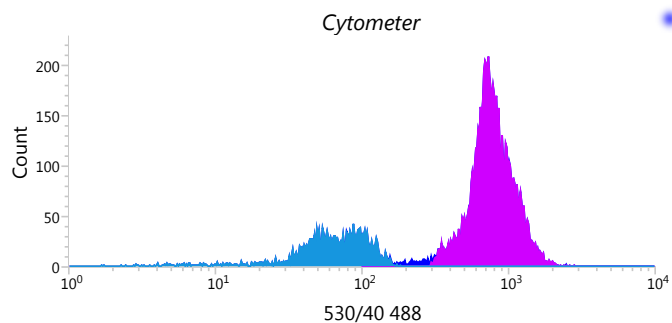

Populations: *Cytometer*

| Populations | Events | % Total | % Parent |
|-------------|--------|---------|----------|
| All Events  | 23,790 | 100.00% | ####     |
| P1          | 11,154 | 46.89%  | 46.89%   |
| P2          | 9,631  | 40.48%  | 86.35%   |
| P3          | 9,631  | 40.48%  | 100.00%  |
| 488+        | 7,159  | 30.09%  | 74.33%   |
| 488 -       | 2,309  | 9.71%   | 23.97%   |

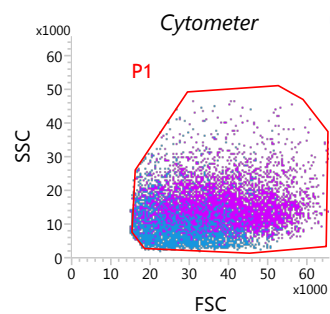

Supplement: Supplementary file 4 — This Tar/Gzip compressed file contains FANS images and gating strategies of the nuclei preparation experiments in this study. [file 41586_2020_3182_MOESM4_ESM.tgz › FANS_images/5B/CEMBA180514-5B.pdf]

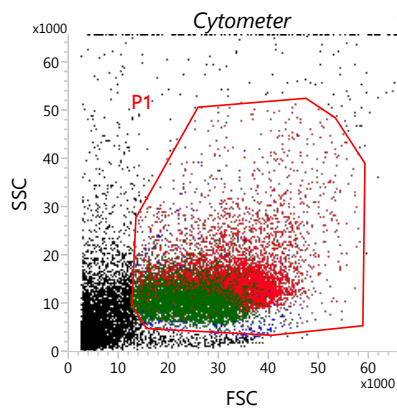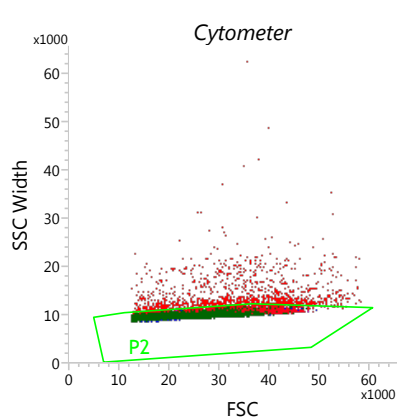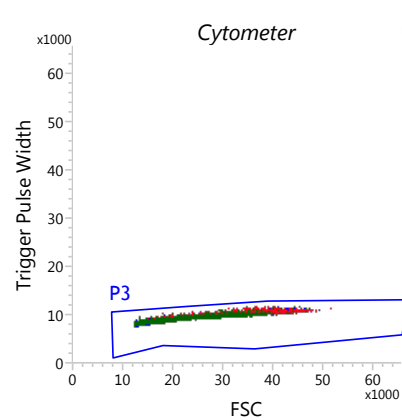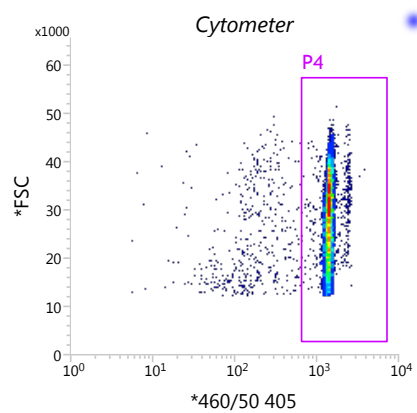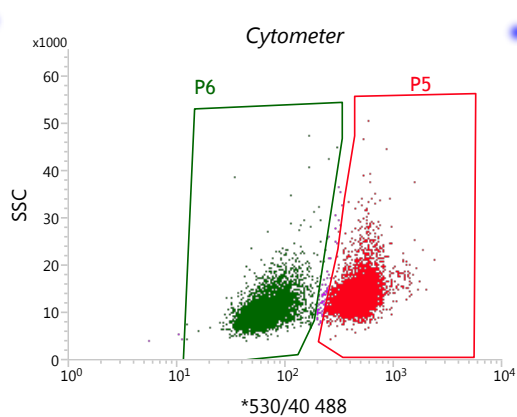

Populations: Cytometer

| Populations | Events | % Total | % Parent |
|-------------|--------|---------|----------|
| All Events  | 20,000 | 100.00% | ####     |
| P1          | 12,525 | 62.62%  | 62.62%   |
| P2          | 10,998 | 54.99%  | 87.81%   |
| P3          | 10,998 | 54.99%  | 100.00%  |
| P4          | 10,575 | 52.88%  | 96.15%   |
| P5          | 6,143  | 30.71%  | 58.09%   |
| P6          | 4,343  | 21.72%  | 41.07%   |

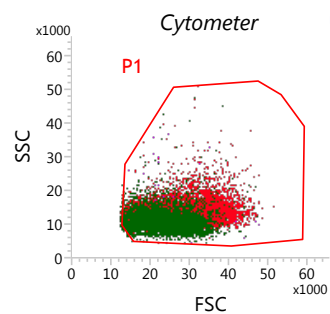

Supplement: Supplementary file 4 — This Tar/Gzip compressed file contains FANS images and gating strategies of the nuclei preparation experiments in this study. [file 41586_2020_3182_MOESM4_ESM.tgz › FANS_images/5E/CEMBA180925-5E.pdf]

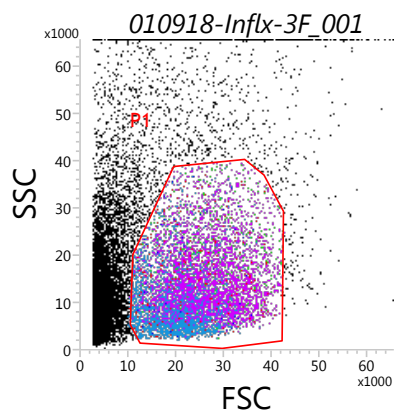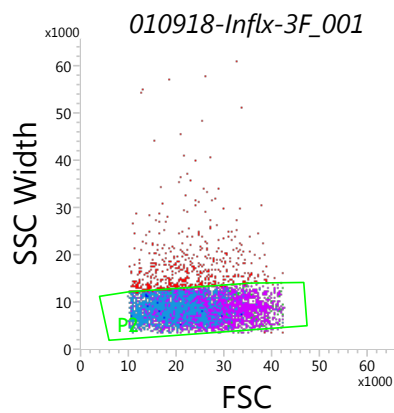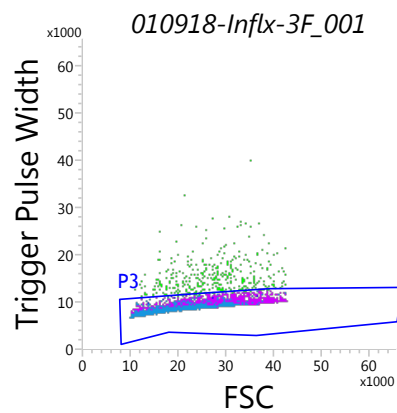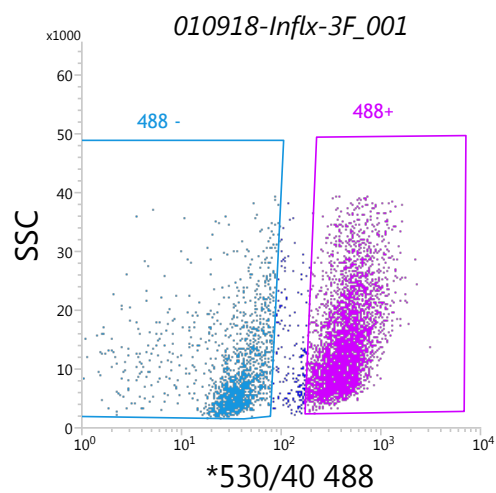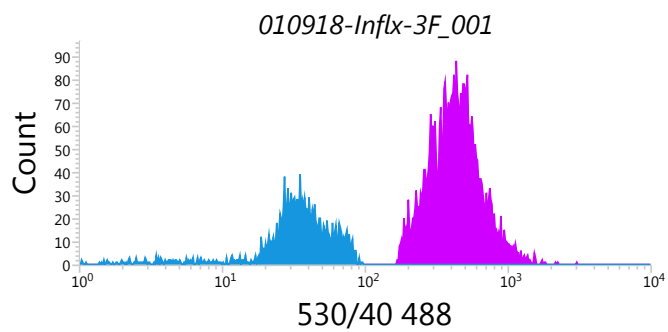

Populations: 010918-Inflx-3F\_001

| Populations | Events | % Total | % Parent |
|-------------|--------|---------|----------|
| All Events  | 16,895 | 100.00% | ####     |
| P1          | 6,947  | 41.12%  | 41.12%   |
| P2          | 6,323  | 37.43%  | 91.02%   |
| P3          | 5,937  | 35.14%  | 93.90%   |
| 488 +       | 4,096  | 24.24%  | 68.99%   |
| 488 -       | 1,658  | 9.81%   | 27.93%   |

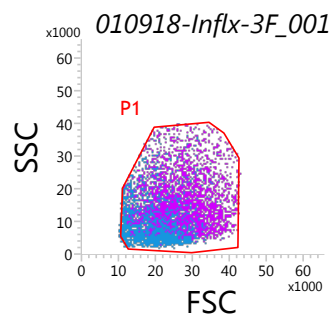

Supplement: Supplementary file 4 — This Tar/Gzip compressed file contains FANS images and gating strategies of the nuclei preparation experiments in this study. [file 41586_2020_3182_MOESM4_ESM.tgz › FANS_images/3F/CEMBA180109-3F.pdf]

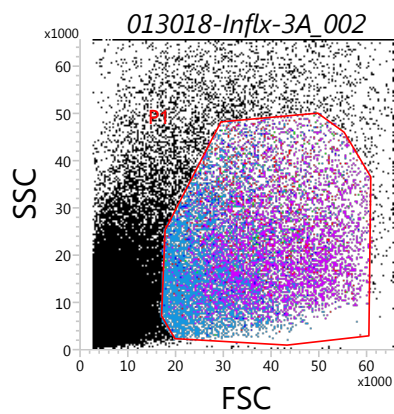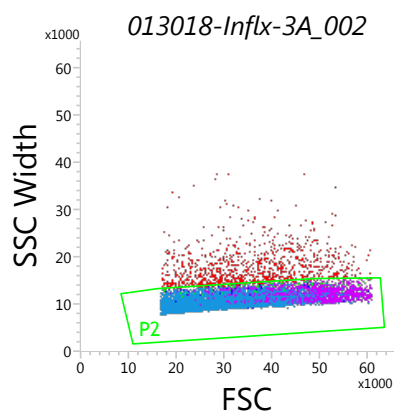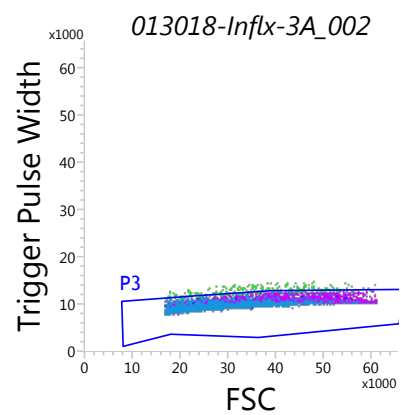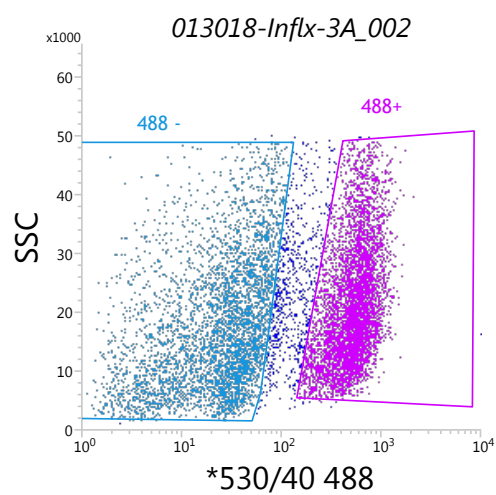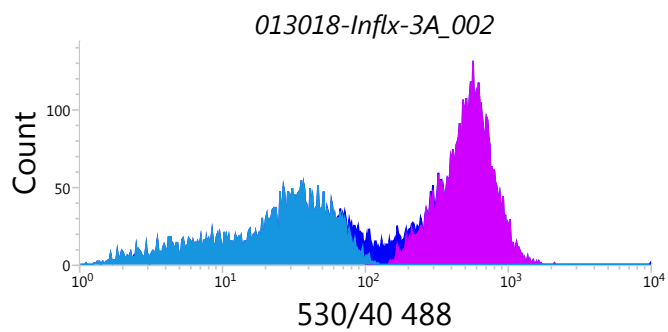

Populations: 013018-Inflx-3A\_002

| Populations | Events | % Total | % Parent |
|-------------|--------|---------|----------|
| All Events  | 69,922 | 100.00% | ####     |
| P1          | 12,153 | 17.38%  | 17.38%   |
| P2          | 11,110 | 15.89%  | 91.42%   |
| P3          | 10,806 | 15.45%  | 97.26%   |
| 488+        | 5,611  | 8.02%   | 51.92%   |
| 488 -       | 4,509  | 6.45%   | 41.73%   |

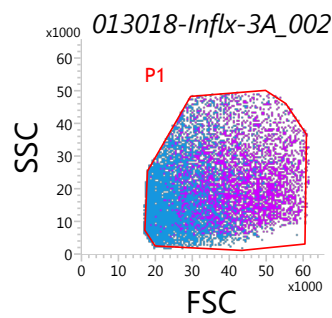

Supplement: Supplementary file 4 — This Tar/Gzip compressed file contains FANS images and gating strategies of the nuclei preparation experiments in this study. [file 41586_2020_3182_MOESM4_ESM.tgz › FANS_images/3A/CEMBA180130-3A.pdf]

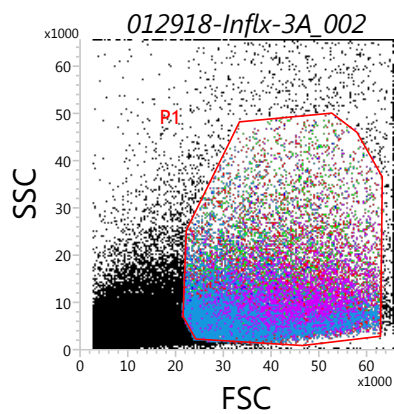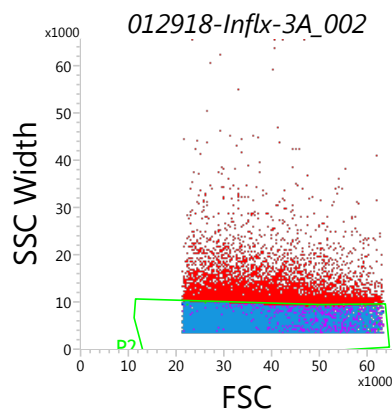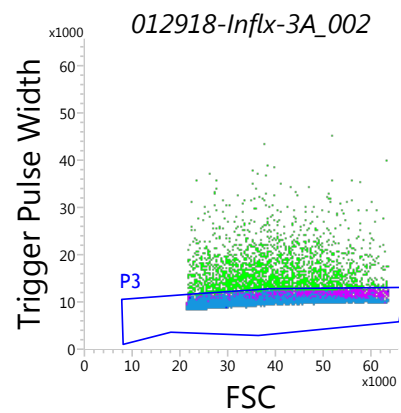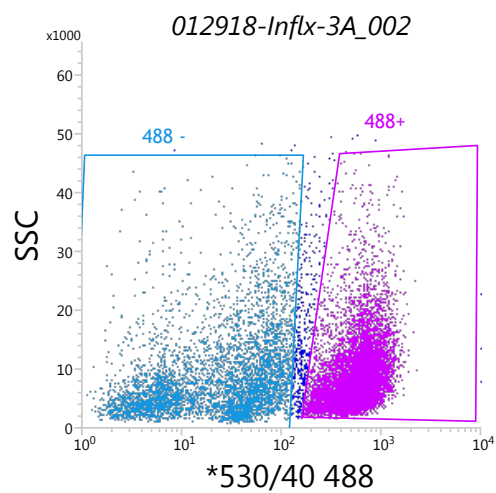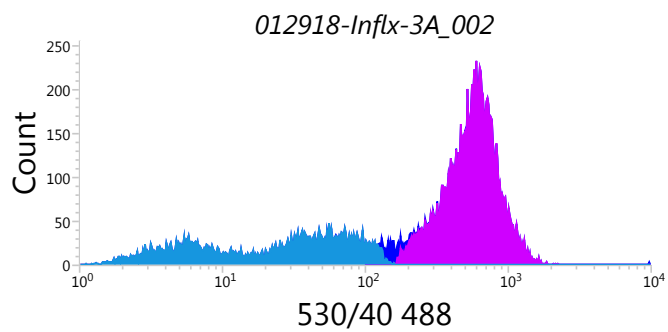

Populations: 012918-Inflx-3A\_002

| Populations | Events  | % Total | % Parent |
|-------------|---------|---------|----------|
| All Events  | 100,000 | 100.00% | ####     |
| P1          | 27,760  | 27.76%  | 27.76%   |
| P2          | 18,589  | 18.59%  | 66.96%   |
| P3          | 15,830  | 15.83%  | 85.16%   |
| 488+        | 10,441  | 10.44%  | 65.96%   |
| 488 -       | 4,971   | 4.97%   | 31.40%   |

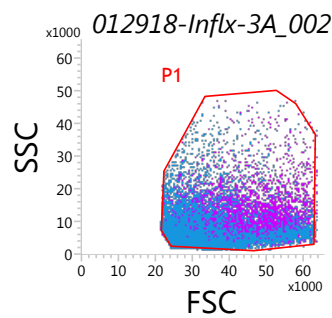

Supplement: Supplementary file 4 — This Tar/Gzip compressed file contains FANS images and gating strategies of the nuclei preparation experiments in this study. [file 41586_2020_3182_MOESM4_ESM.tgz › FANS_images/3A/CEMBA180129-3A.pdf]

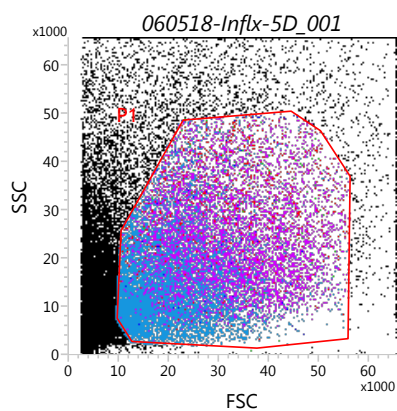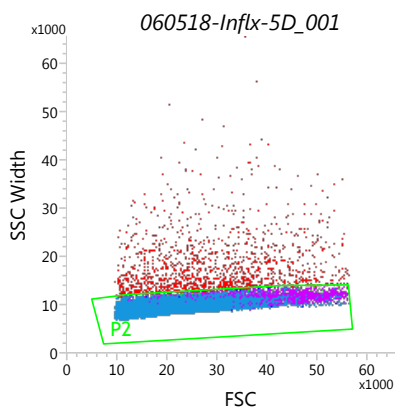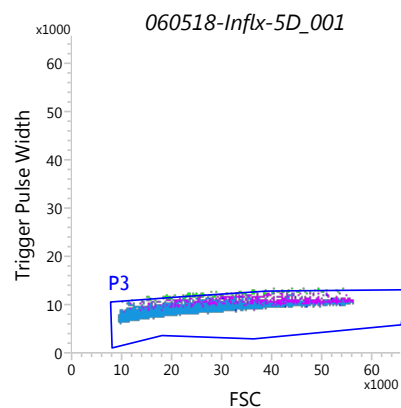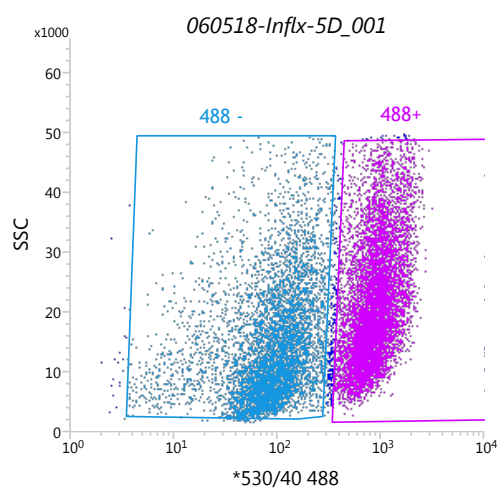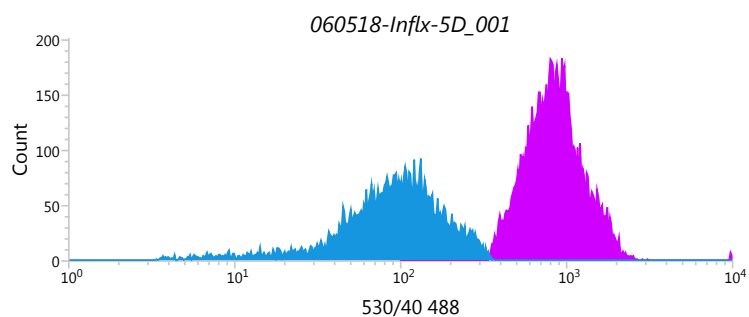

Populations: 060518-Inflx-5D\_001

| Populations | Events | % Total | % Parent |
|-------------|--------|---------|----------|
| All Events  | 44,072 | 100.00% | ####     |
| P1          | 17,062 | 38.71%  | 38.71%   |
| P2          | 15,543 | 35.27%  | 91.10%   |
| P3          | 15,406 | 34.96%  | 99.12%   |
| 488+        | 8,729  | 19.81%  | 56.66%   |
| 488 -       | 6,379  | 14.47%  | 41.41%   |

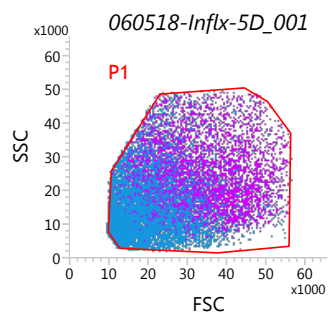

Supplement: Supplementary file 4 — This Tar/Gzip compressed file contains FANS images and gating strategies of the nuclei preparation experiments in this study. [file 41586_2020_3182_MOESM4_ESM.tgz › FANS_images/5D/CEMBA180605-5D.pdf]

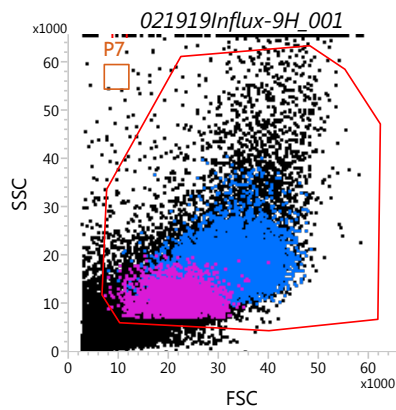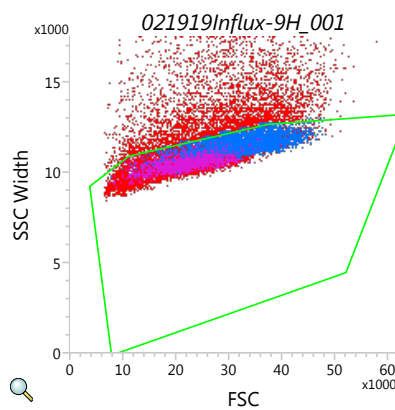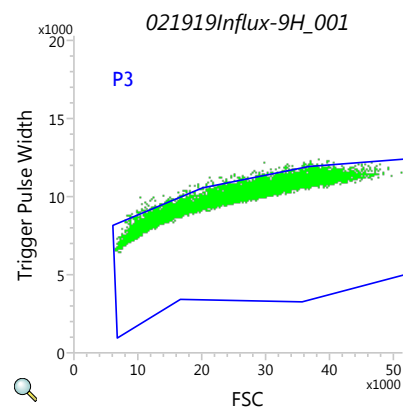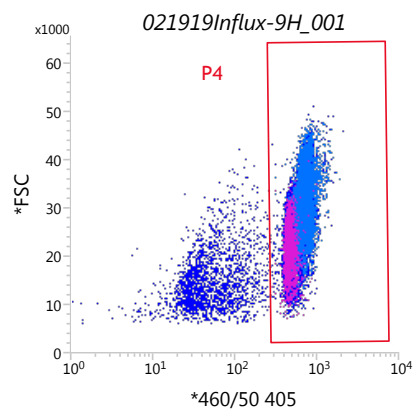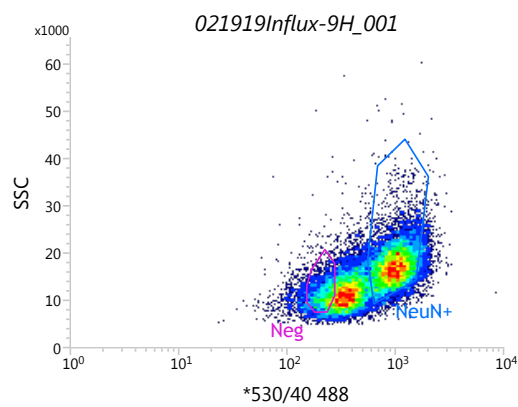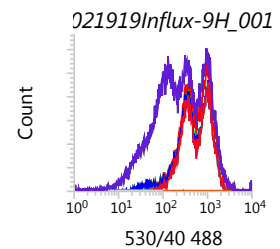

Populations: 021919Influx-9H\_001

| Populations | Events | % Total | % Parent |
|-------------|--------|---------|----------|
| All Events  | 50,000 | 100.00% | ####     |
| P1          | 25,237 | 50.47%  | 50.47%   |
| P2          | 20,498 | 41.00%  | 81.22%   |
| P3          | 20,387 | 40.77%  | 99.46%   |
| P4          | 18,229 | 36.46%  | 89.41%   |
| NeuN+       | 8,283  | 16.57%  | 45.44%   |
| Neg         | 1,857  | 3.71%   | 10.19%   |
| P7          | 1      | 0.00%   | 0.00%    |
| NOT(P7)     | 49,999 | 100.00% | 100.00%  |

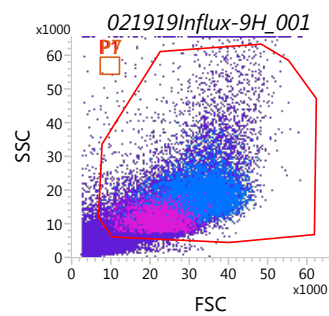

Supplement: Supplementary file 4 — This Tar/Gzip compressed file contains FANS images and gating strategies of the nuclei preparation experiments in this study. [file 41586_2020_3182_MOESM4_ESM.tgz › FANS_images/9H/CEMBA190219-9H.pdf]

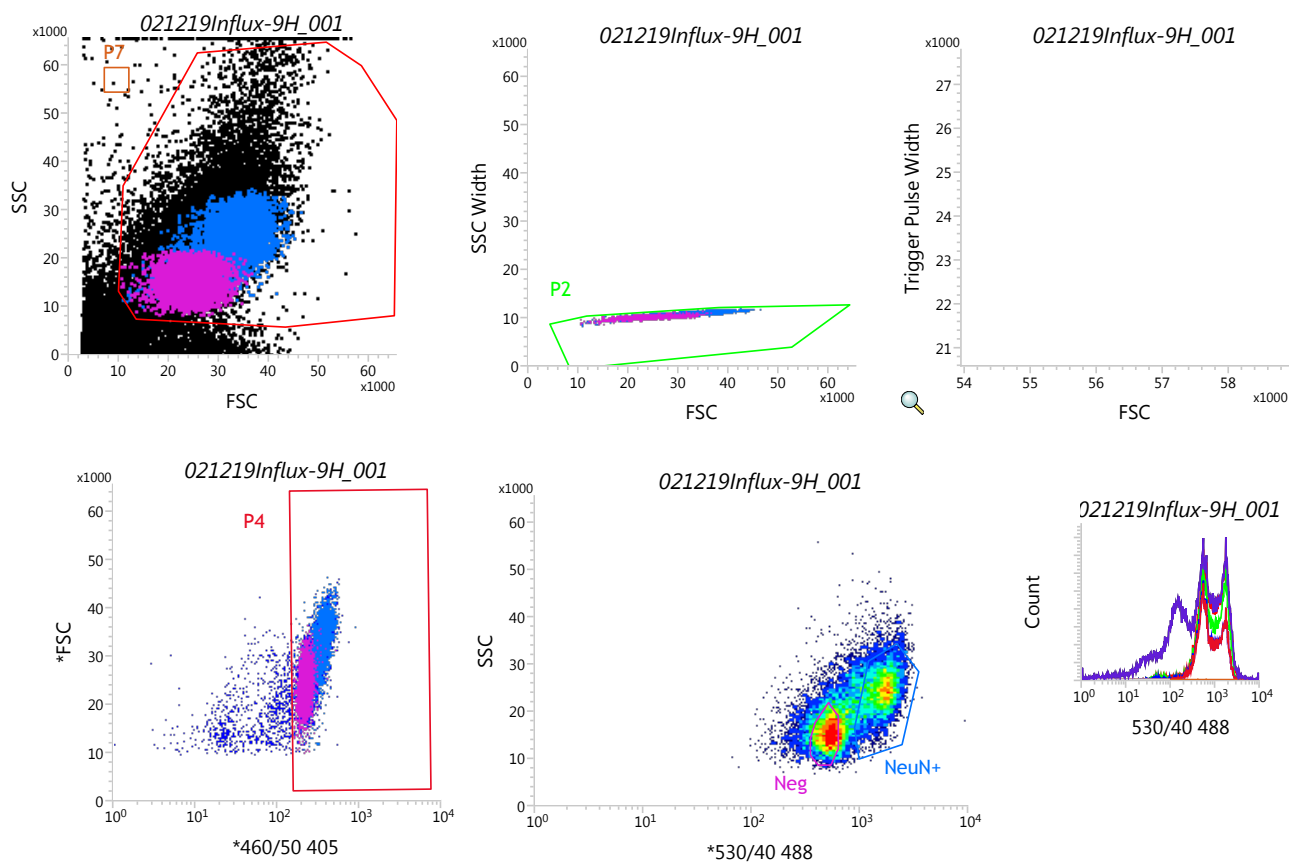

Populations: 021219Influx-9H\_001

| Populations | Events | % Total | % Parent |
|-------------|--------|---------|----------|
| All Events  | 50,000 | 100.00% | ####     |
| P1          | 28,530 | 57.06%  | 57.06%   |
| P2          | 22,872 | 45.74%  | 80.17%   |
| P3          | 16,070 | 32.14%  | 70.26%   |
| P4          | 15,200 | 30.40%  | 94.59%   |
| NeuN+       | 5,449  | 10.90%  | 35.85%   |
| Neg         | 4,585  | 9.17%   | 30.16%   |
| P7          | 2      | 0.00%   | 0.00%    |
| NOT(P7)     | 49,998 | 100.00% | 100.00%  |

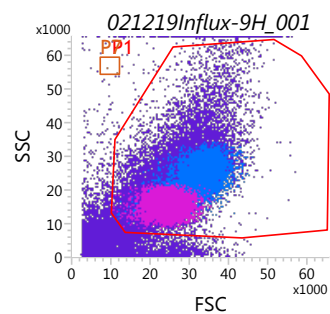

Supplement: Supplementary file 4 — This Tar/Gzip compressed file contains FANS images and gating strategies of the nuclei preparation experiments in this study. [file 41586_2020_3182_MOESM4_ESM.tgz › FANS_images/9H/CEMBA190212-9H.pdf]

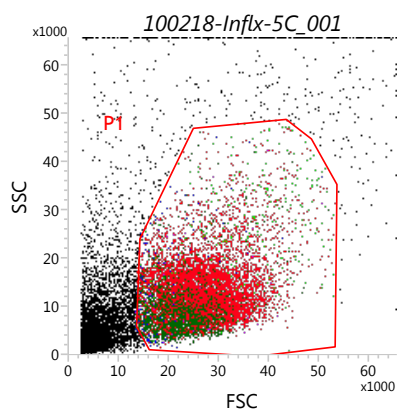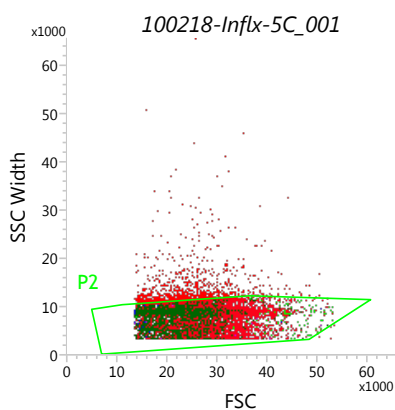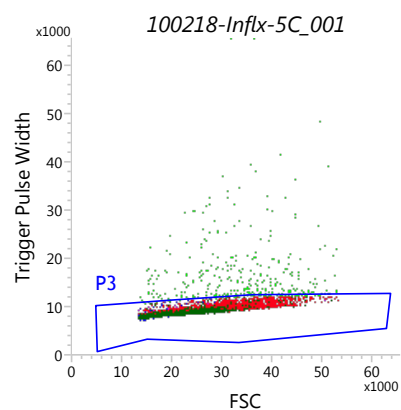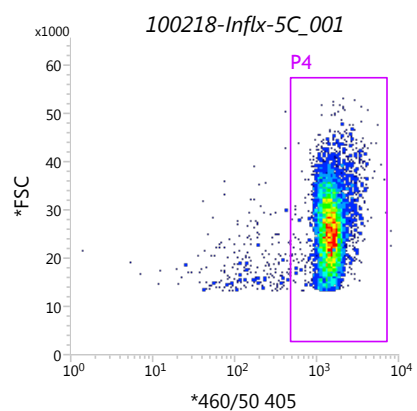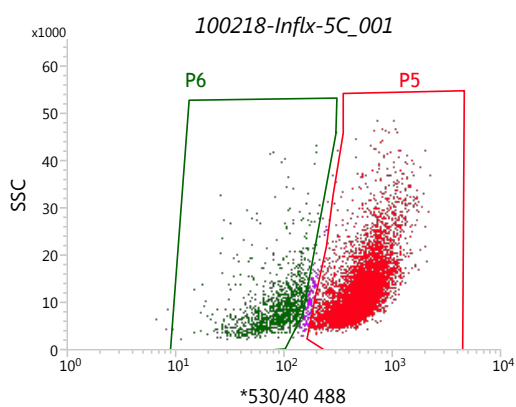

Populations: 100218-Inflx-5C\_001

| Populations | Events | % Total | % Parent |
|-------------|--------|---------|----------|
| All Events  | 16,495 | 100.00% | ####     |
| P1          | 9,430  | 57.17%  | 57.17%   |
| P2          | 8,117  | 49.21%  | 86.08%   |
| P3          | 7,770  | 47.11%  | 95.73%   |
| P4          | 7,532  | 45.66%  | 96.94%   |
| P5          | 6,087  | 36.90%  | 80.82%   |
| P6          | 1,303  | 7.90%   | 17.30%   |

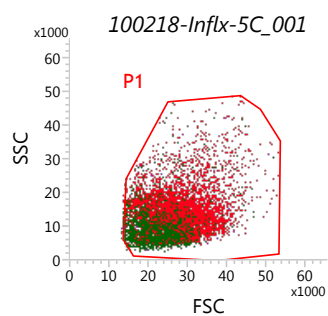

Supplement: Supplementary file 4 — This Tar/Gzip compressed file contains FANS images and gating strategies of the nuclei preparation experiments in this study. [file 41586_2020_3182_MOESM4_ESM.tgz › FANS_images/5C/CEMBA181002-5C.pdf]

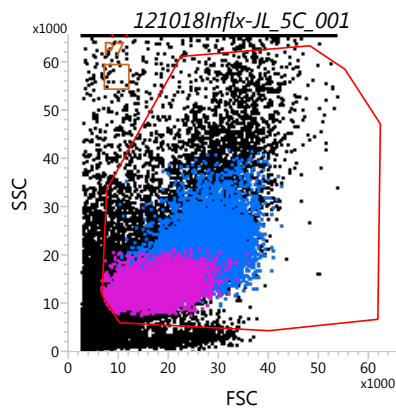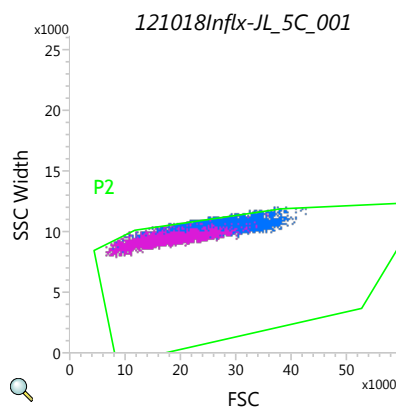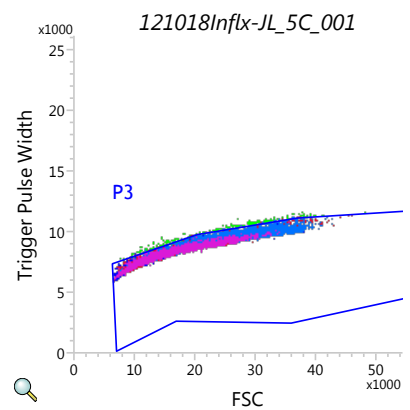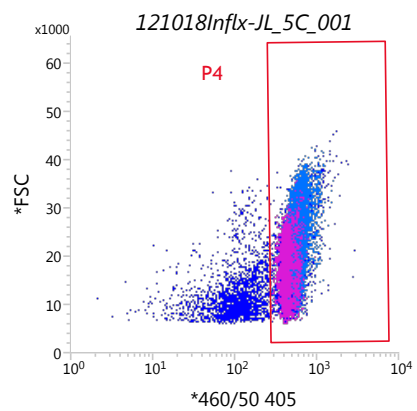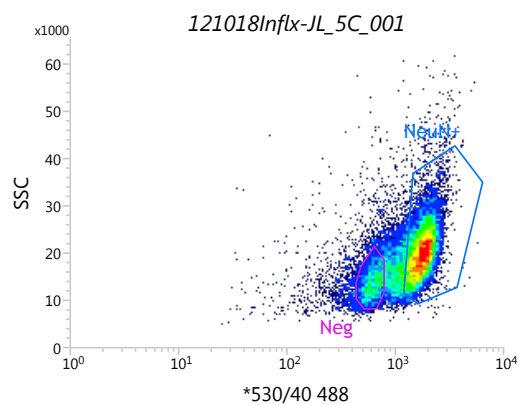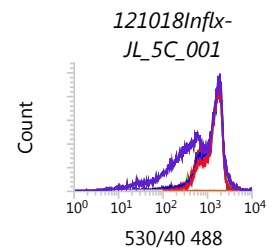

Populations: 121018Inflx-JL\_5C\_001

| Populations | Events | % Total | % Parent |
|-------------|--------|---------|----------|
| All Events  | 42,678 | 100.00% | ####     |
| P1          | 25,749 | 60.33%  | 60.33%   |
| P2          | 22,346 | 52.36%  | 86.78%   |
| P3          | 22,030 | 51.62%  | 98.59%   |
| P4          | 20,012 | 46.89%  | 90.84%   |
| NeuN+       | 12,501 | 29.29%  | 62.47%   |
| Neg         | 2,659  | 6.23%   | 13.29%   |
| P7          | 14     | 0.03%   | 0.03%    |
| NOT(P7)     | 42,664 | 99.97%  | 99.97%   |

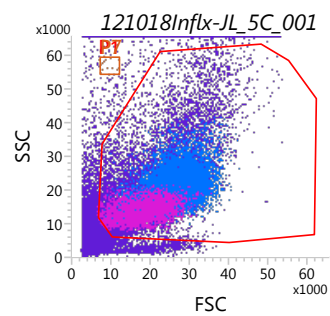

Supplement: Supplementary file 4 — This Tar/Gzip compressed file contains FANS images and gating strategies of the nuclei preparation experiments in this study. [file 41586_2020_3182_MOESM4_ESM.tgz › FANS_images/5C/CEMBA181210-5C.pdf]

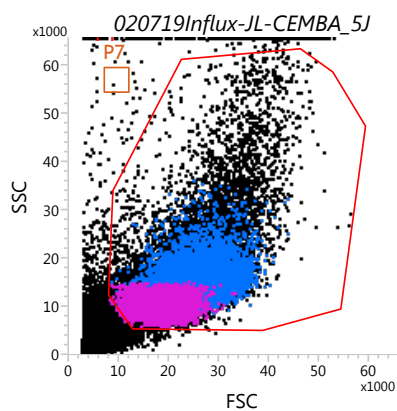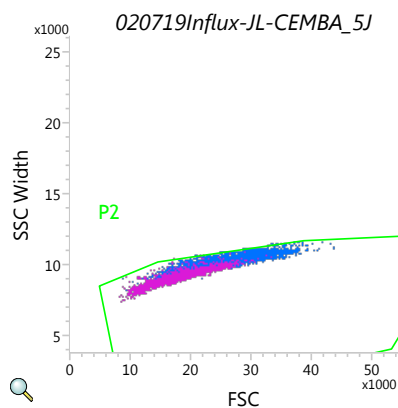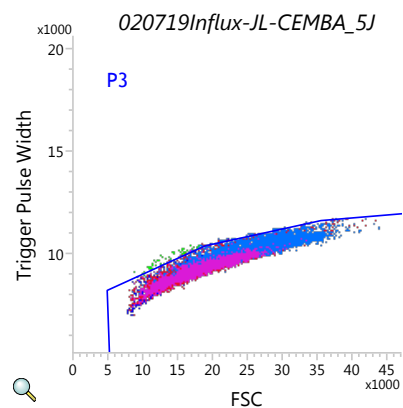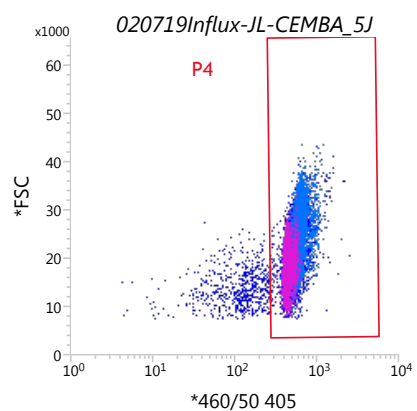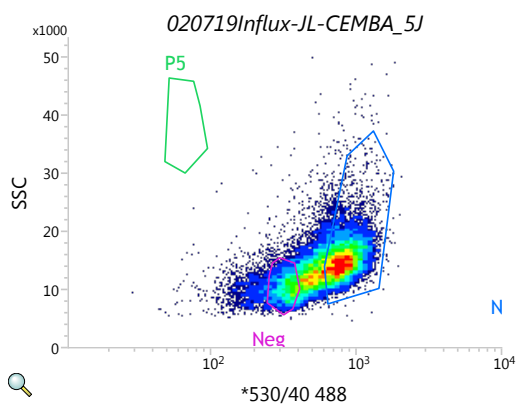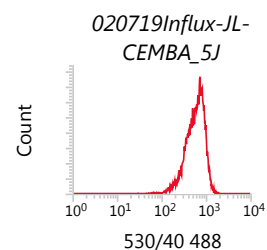

Populations: 020719Influx-JL-CEMBA\_5J

| Populations | Events | % Total | % Parent |
|-------------|--------|---------|----------|
| All Events  | 39,318 | 100.00% | ####     |
| P1          | 26,871 | 68.34%  | 68.34%   |
| P2          | 20,647 | 52.51%  | 76.84%   |
| P3          | 20,600 | 52.39%  | 99.77%   |
| P4          | 20,000 | 50.87%  | 97.09%   |
| NeuN+       | 9,695  | 24.66%  | 48.48%   |
| Neg         | 3,040  | 7.73%   | 15.20%   |
| P5          | 0      | 0.00%   | 0.00%    |
| P7          | 1      | 0.00%   | 0.00%    |
| NOT(P7)     | 39,317 | 100.00% | 100.00%  |

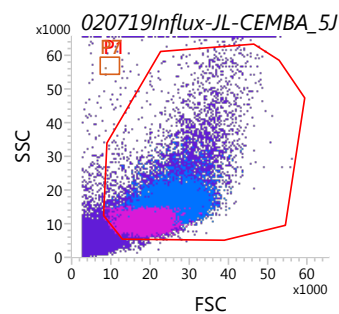

Supplement: Supplementary file 4 — This Tar/Gzip compressed file contains FANS images and gating strategies of the nuclei preparation experiments in this study. [file 41586_2020_3182_MOESM4_ESM.tgz › FANS_images/5J/CEMBA190207-5J.pdf]

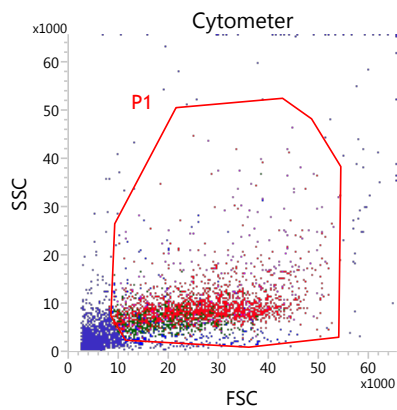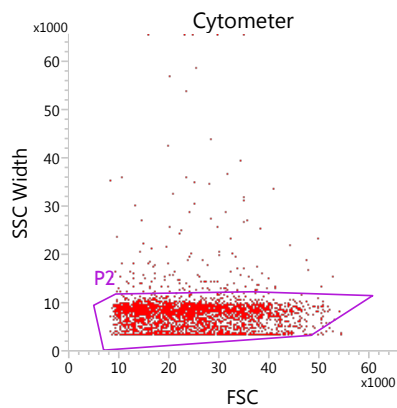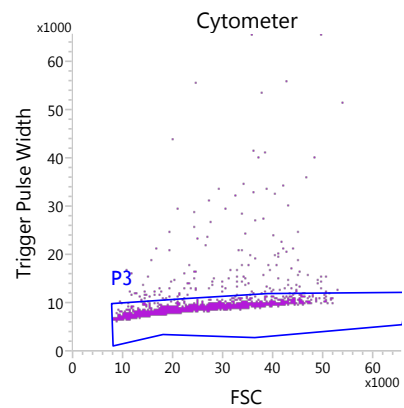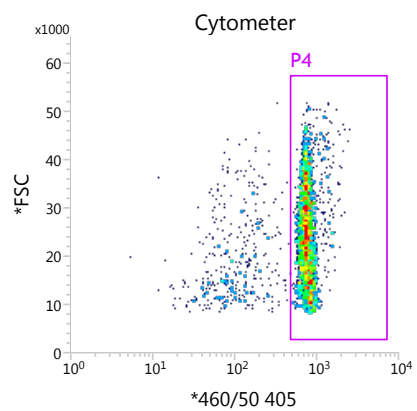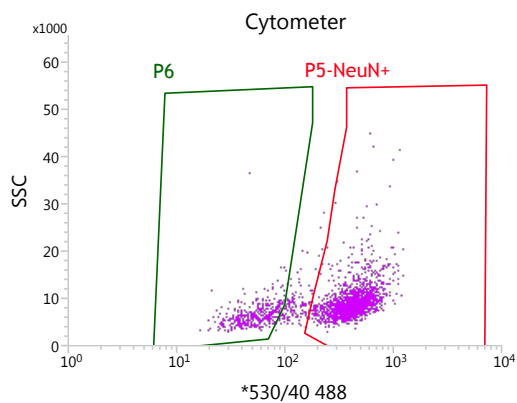

Populations: Cytometer

| Populations | Events | % Total | % Parent |
|-------------|--------|---------|----------|
| All Events  | 5,000  | 100.00% | ####     |
| P1          | 2,973  | 59.46%  | 59.46%   |
| P2          | 2,801  | 56.02%  | 94.21%   |
| P3          | 2,629  | 52.58%  | 93.86%   |
| P4          | 2,269  | 45.38%  | 86.31%   |
| P5-NeuN+    | 1,753  | 35.06%  | 77.26%   |
| P6          | 396    | 7.92%   | 17.45%   |
| NOT(P1)     | 2,027  | 40.54%  | 40.54%   |

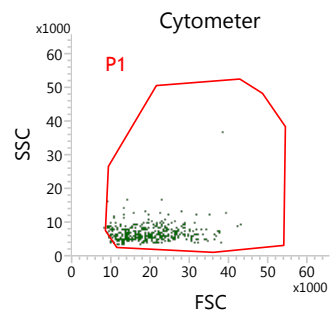

Supplement: Supplementary file 4 — This Tar/Gzip compressed file contains FANS images and gating strategies of the nuclei preparation experiments in this study. [file 41586_2020_3182_MOESM4_ESM.tgz › FANS_images/6B/CEMBA181022-6B .pdf]

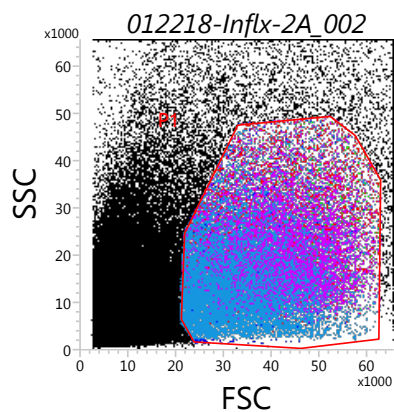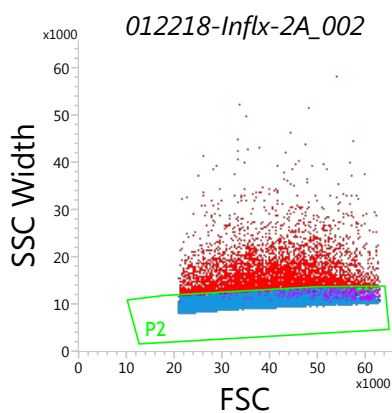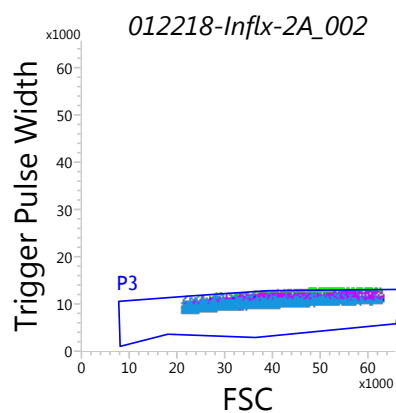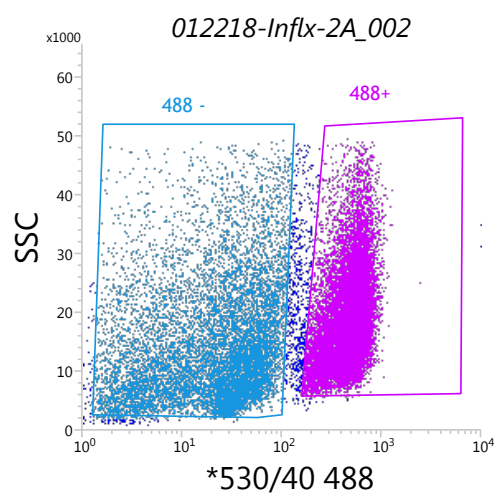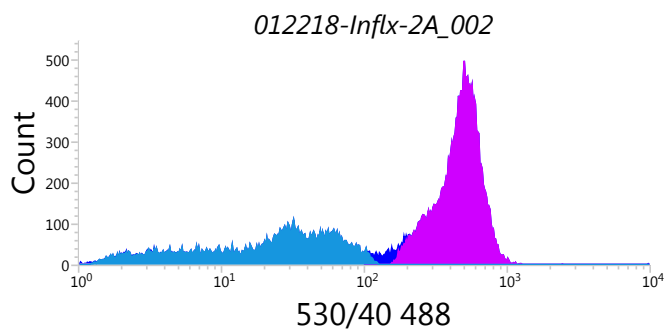

Populations: 012218-Inflx-2A\_002

| Populations | Events  | % Total | % Parent |
|-------------|---------|---------|----------|
| All Events  | 200,000 | 100.00% | ####     |
| P1          | 35,124  | 17.56%  | 17.56%   |
| P2          | 29,746  | 14.87%  | 84.69%   |
| P3          | 29,297  | 14.65%  | 98.49%   |
| 488+        | 18,017  | 9.01%   | 61.50%   |
| 488 -       | 10,503  | 5.25%   | 35.85%   |

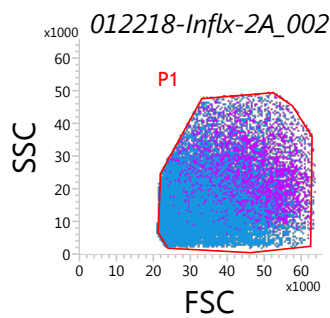

Supplement: Supplementary file 4 — This Tar/Gzip compressed file contains FANS images and gating strategies of the nuclei preparation experiments in this study. [file 41586_2020_3182_MOESM4_ESM.tgz › FANS_images/2A/CEMBA180122-2A.pdf]

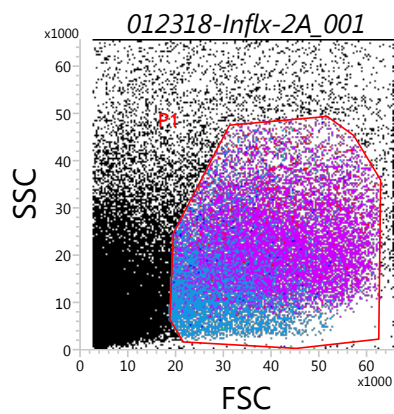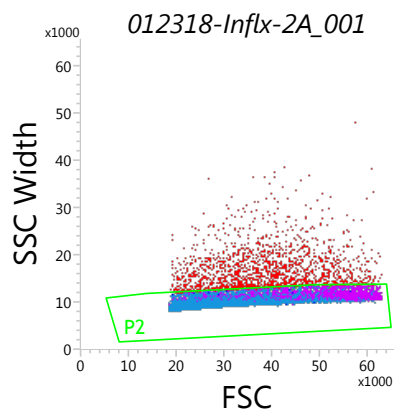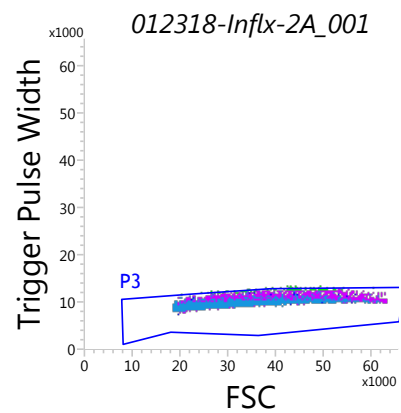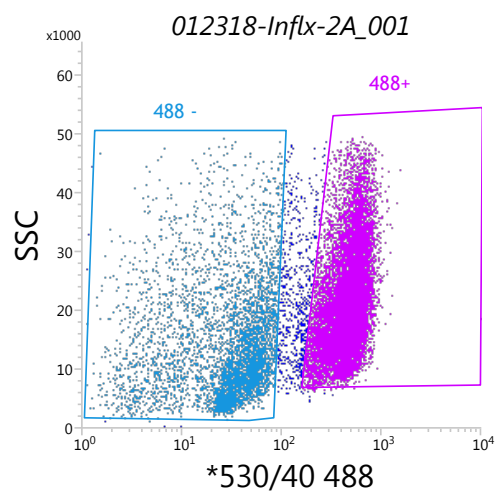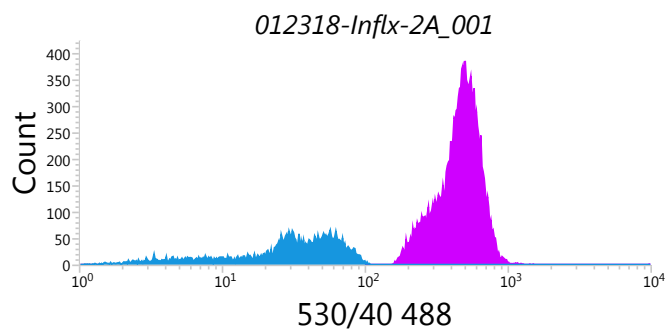

Populations: 012318-Inflx-2A\_001

| Populations | Events | % Total | % Parent |
|-------------|--------|---------|----------|
| All Events  | 62,546 | 100.00% | ####     |
| P1          | 21,739 | 34.76%  | 34.76%   |
| P2          | 19,929 | 31.86%  | 91.67%   |
| P3          | 19,820 | 31.69%  | 99.45%   |
| 488+        | 14,245 | 22.78%  | 71.87%   |
| 488 -       | 4,929  | 7.88%   | 24.87%   |

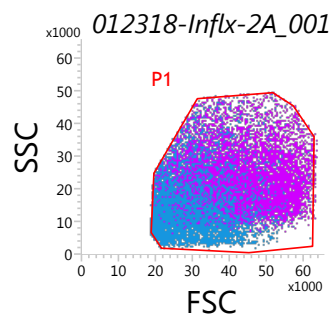

Supplement: Supplementary file 4 — This Tar/Gzip compressed file contains FANS images and gating strategies of the nuclei preparation experiments in this study. [file 41586_2020_3182_MOESM4_ESM.tgz › FANS_images/2A/CEMBA180123-2A.pdf]

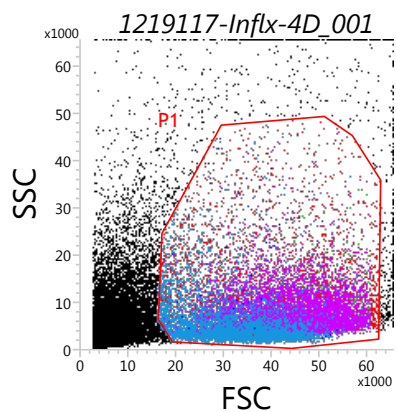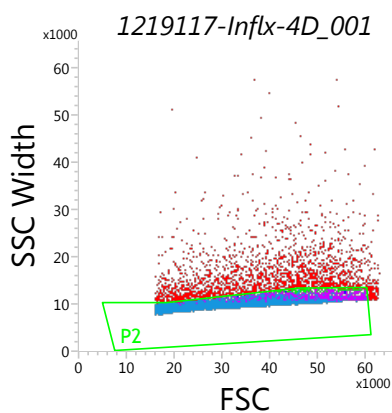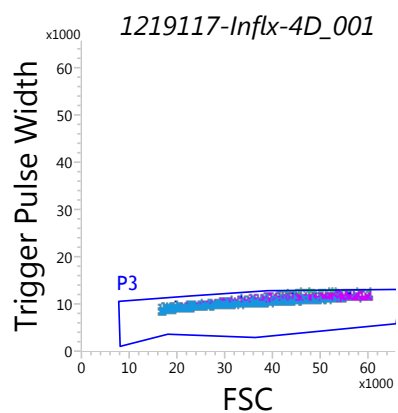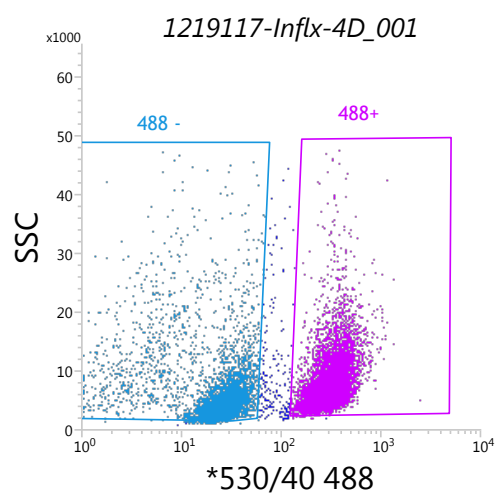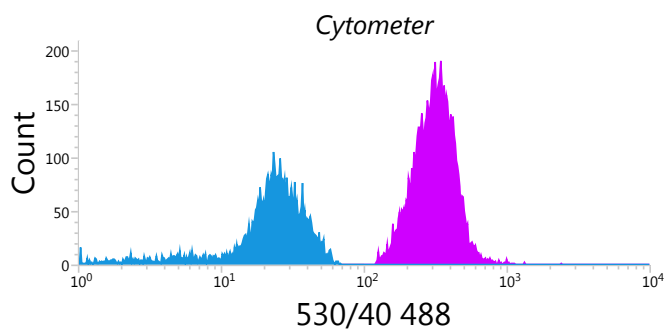

Populations: 1219117-Inflx-4D\_001

| Populations | Events | % Total | % Parent |
|-------------|--------|---------|----------|
| All Events  | 36,148 | 100.00% | ####     |
| P1          | 14,969 | 41.41%  | 41.41%   |
| P2          | 12,713 | 35.17%  | 84.93%   |
| P3          | 12,549 | 34.72%  | 98.71%   |
| 488+        | 7,511  | 20.78%  | 59.85%   |
| 488-        | 4,845  | 13.40%  | 38.61%   |

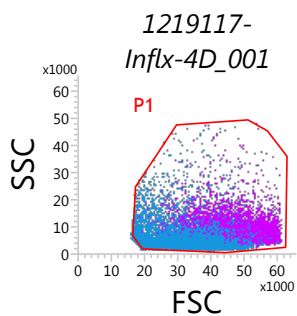

Supplement: Supplementary file 4 — This Tar/Gzip compressed file contains FANS images and gating strategies of the nuclei preparation experiments in this study. [file 41586_2020_3182_MOESM4_ESM.tgz › FANS_images/4D/CEMBA171219-4D.pdf]

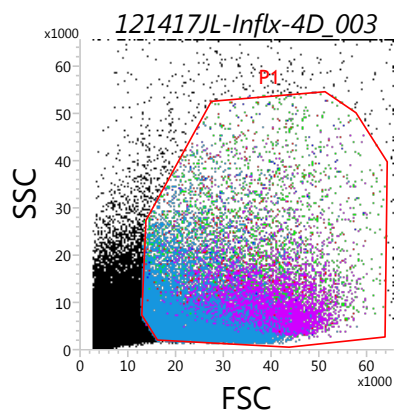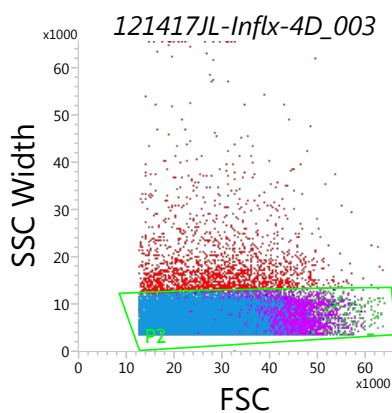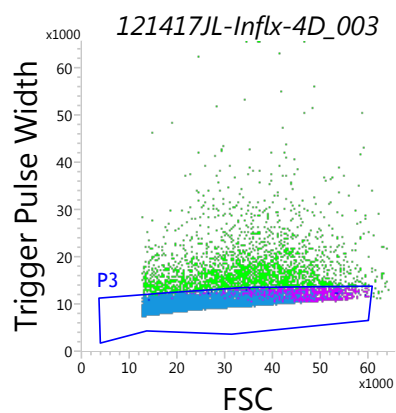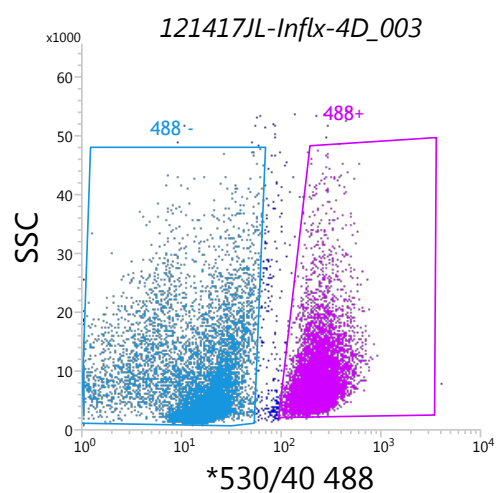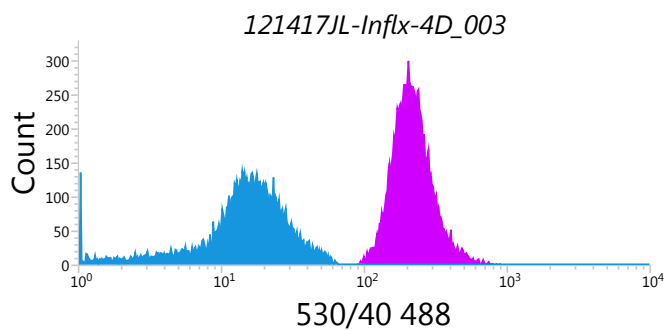

Populations: 121417JL-Inflx-4D\_003

| Populations | Events | % Total | % Parent |
|-------------|--------|---------|----------|
| All Events  | 73,711 | 100.00% | ####     |
| P1          | 25,622 | 34.76%  | 34.76%   |
| P2          | 23,423 | 31.78%  | 91.42%   |
| P3          | 20,679 | 28.05%  | 88.29%   |
| 488+        | 10,896 | 14.78%  | 52.69%   |
| 488 -       | 9,528  | 12.93%  | 46.08%   |

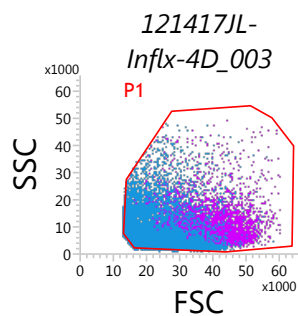

Supplement: Supplementary file 4 — This Tar/Gzip compressed file contains FANS images and gating strategies of the nuclei preparation experiments in this study. [file 41586_2020_3182_MOESM4_ESM.tgz › FANS_images/4D/CEMBA171214-4D.pdf]

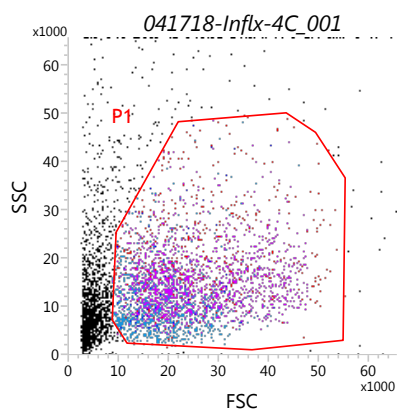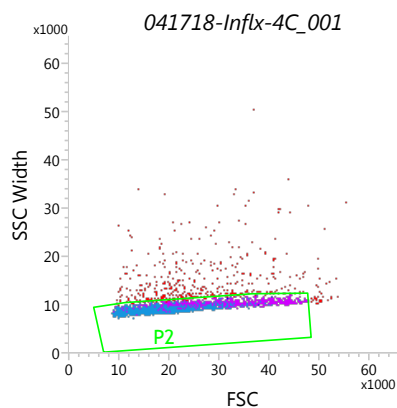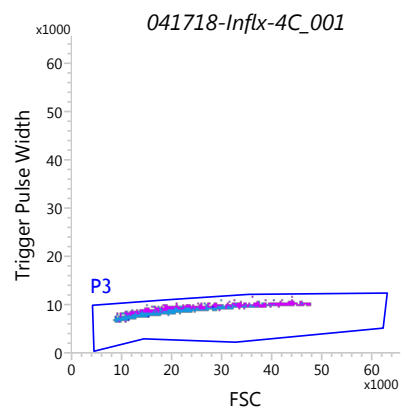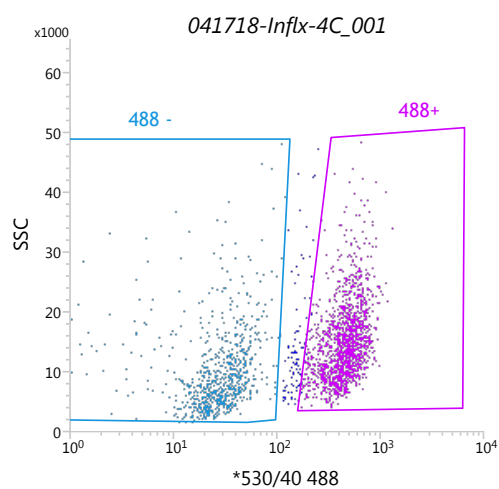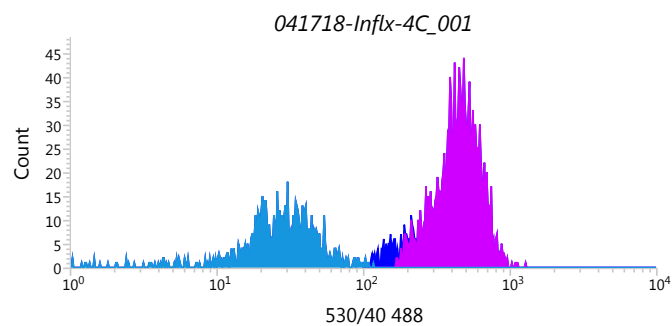

Populations: 041718-Inflx-4C\_001

| Populations | Events | % Total | % Parent |
|-------------|--------|---------|----------|
| All Events  | 4,855  | 100.00% | ####     |
| P1          | 2,900  | 59.73%  | 59.73%   |
| P2          | 2,427  | 49.99%  | 83.69%   |
| P3          | 2,427  | 49.99%  | 100.00%  |
| 488+        | 1,548  | 31.88%  | 63.78%   |
| 488 -       | 777    | 16.00%  | 32.01%   |

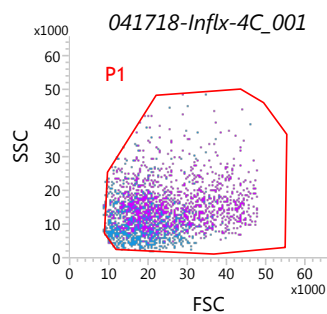

Supplement: Supplementary file 4 — This Tar/Gzip compressed file contains FANS images and gating strategies of the nuclei preparation experiments in this study. [file 41586_2020_3182_MOESM4_ESM.tgz › FANS_images/4C/CEMBA180417-4C.pdf]

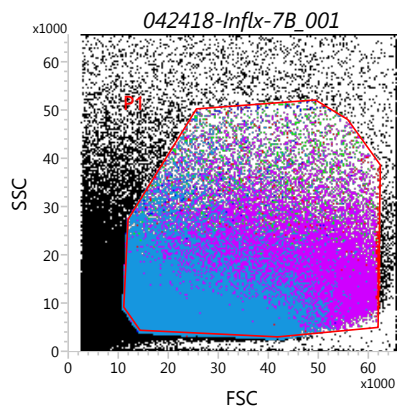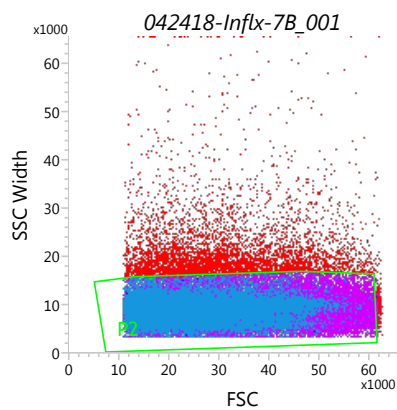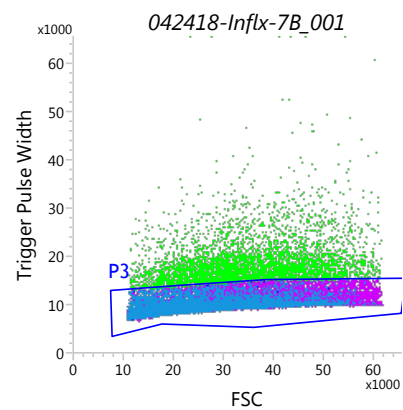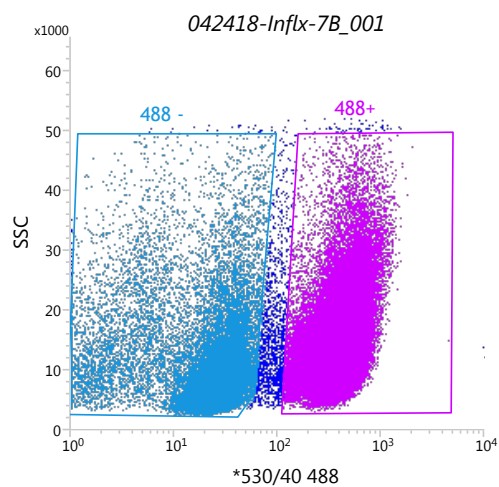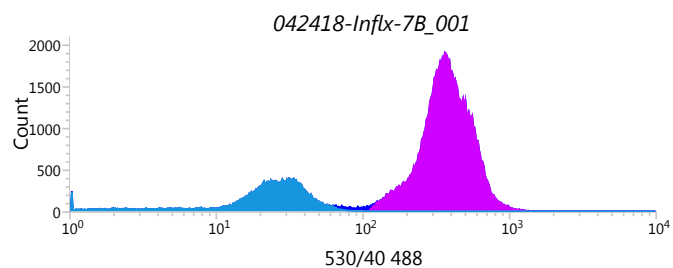

Populations: 042418-Inflx-7B\_001

| Populations | Events  | % Total | % Parent |
|-------------|---------|---------|----------|
| All Events  | 203,642 | 100.00% | ####     |
| P1          | 127,601 | 62.66%  | 62.66%   |
| P2          | 122,219 | 60.02%  | 95.78%   |
| P3          | 117,019 | 57.46%  | 95.75%   |
| 488+        | 89,722  | 44.06%  | 76.67%   |
| 488 -       | 25,753  | 12.65%  | 22.01%   |

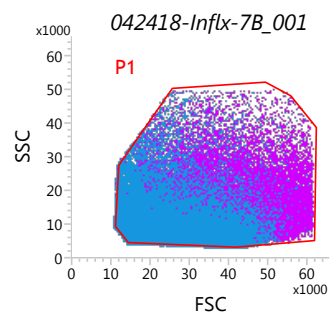

Supplement: Supplementary file 4 — This Tar/Gzip compressed file contains FANS images and gating strategies of the nuclei preparation experiments in this study. [file 41586_2020_3182_MOESM4_ESM.tgz › FANS_images/7B/CEMBA180424-7B.pdf]

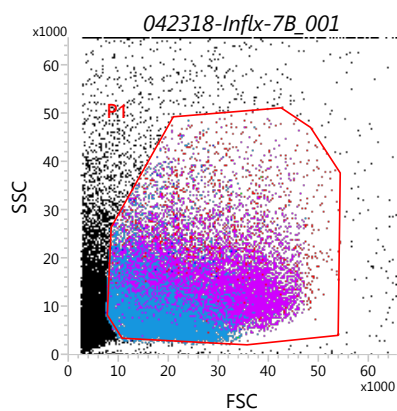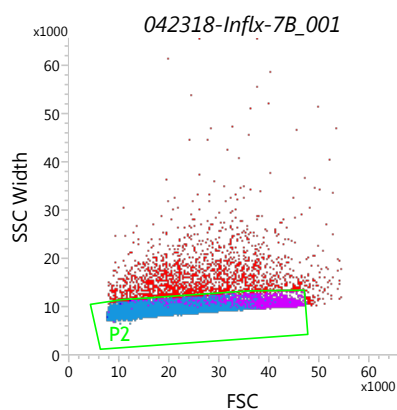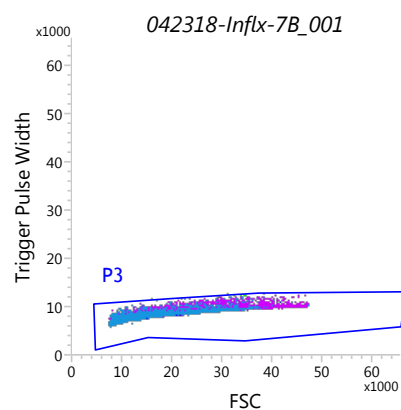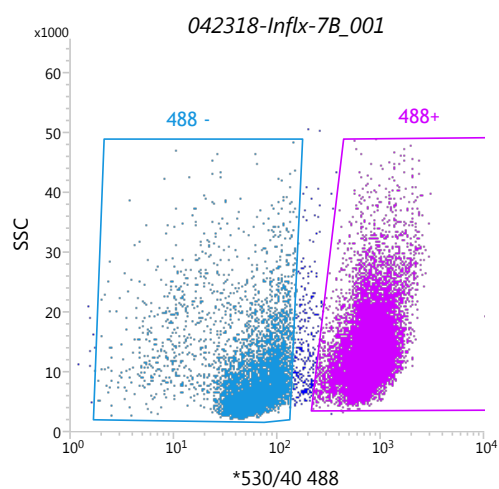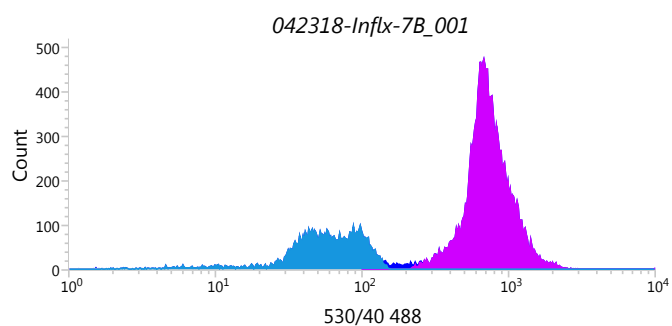

Populations: 042318-Inflx-7B\_001

| Populations | Events | % Total | % Parent |
|-------------|--------|---------|----------|
| All Events  | 35,039 | 100.00% | ####     |
| P1          | 25,441 | 72.61%  | 72.61%   |
| P2          | 23,239 | 66.32%  | 91.34%   |
| P3          | 23,229 | 66.29%  | 99.96%   |
| 488+        | 16,641 | 47.49%  | 71.64%   |
| 488 -       | 6,293  | 17.96%  | 27.09%   |

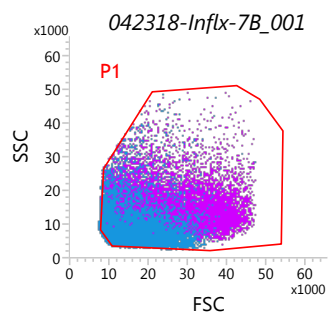

Supplement: Supplementary file 4 — This Tar/Gzip compressed file contains FANS images and gating strategies of the nuclei preparation experiments in this study. [file 41586_2020_3182_MOESM4_ESM.tgz › FANS_images/7B/CEMBA180423-7B.pdf]

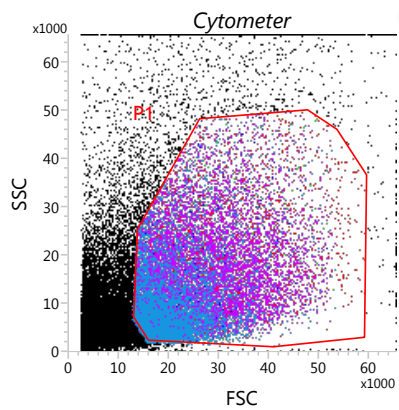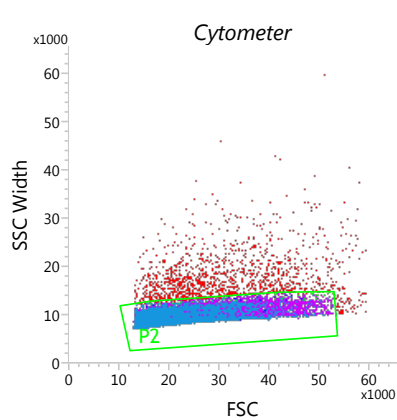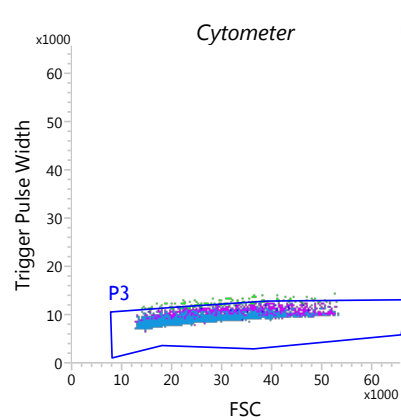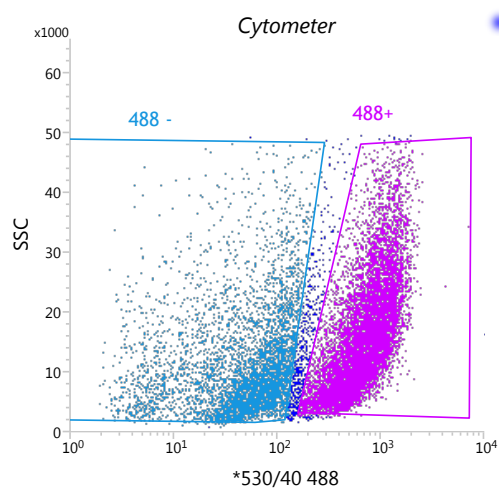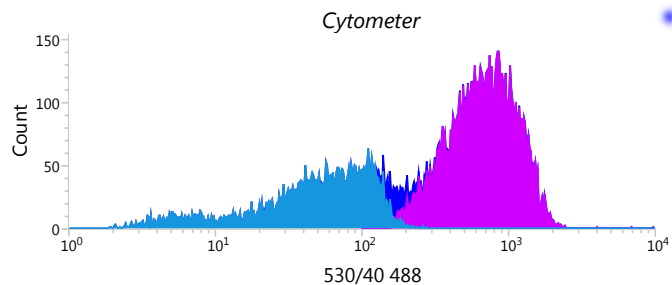

Populations: *Cytometer*

| Populations | Events | % Total | % Parent |
|-------------|--------|---------|----------|
| All Events  | 37,717 | 100.00% | ####     |
| P1          | 16,641 | 44.12%  | 44.12%   |
| P2          | 15,270 | 40.49%  | 91.76%   |
| P3          | 15,113 | 40.07%  | 98.97%   |
| 488 +       | 9,592  | 25.43%  | 63.47%   |
| 488 -       | 5,020  | 13.31%  | 33.22%   |

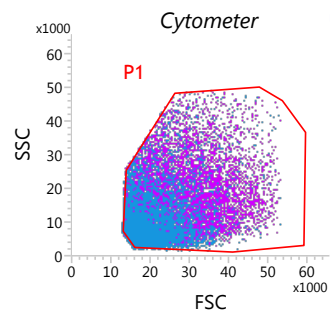

Supplement: Supplementary file 4 — This Tar/Gzip compressed file contains FANS images and gating strategies of the nuclei preparation experiments in this study. [file 41586_2020_3182_MOESM4_ESM.tgz › FANS_images/1A/CEMBA180226-1A.pdf]

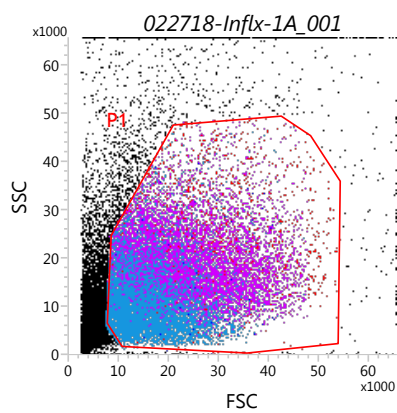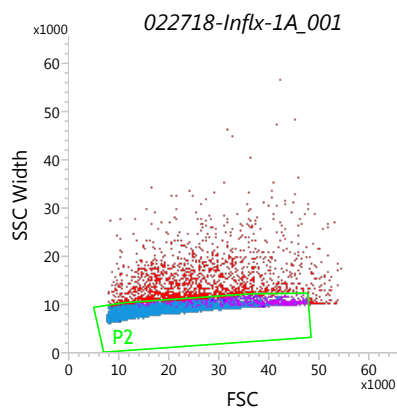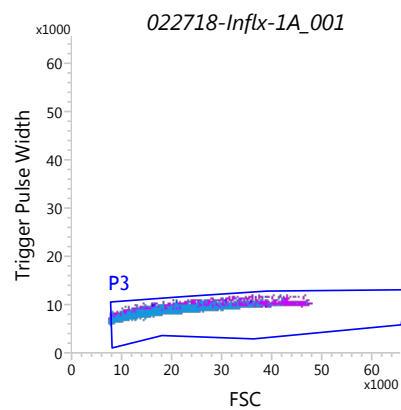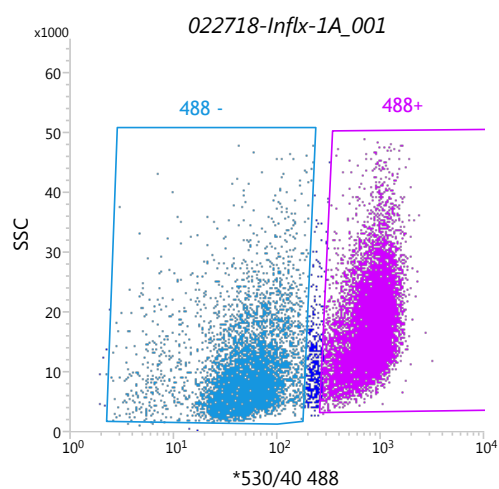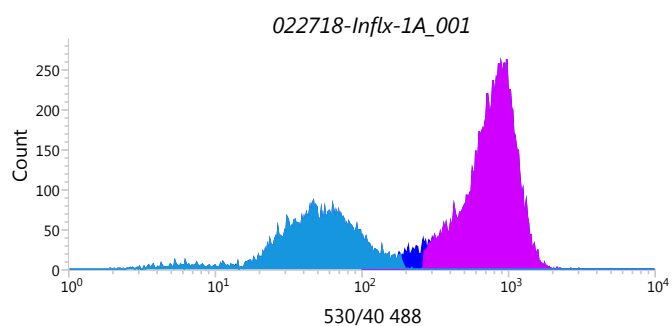

Populations: 022718-Inflx-1A\_001

| Populations | Events | % Total | % Parent |
|-------------|--------|---------|----------|
| All Events  | 27,791 | 100.00% | ####     |
| P1          | 19,792 | 71.22%  | 71.22%   |
| P2          | 17,774 | 63.96%  | 89.80%   |
| P3          | 17,774 | 63.96%  | 100.00%  |
| 488+        | 11,292 | 40.63%  | 63.53%   |
| 488 -       | 6,023  | 21.67%  | 33.89%   |

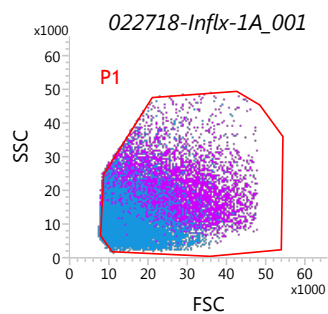

Supplement: Supplementary file 4 — This Tar/Gzip compressed file contains FANS images and gating strategies of the nuclei preparation experiments in this study. [file 41586_2020_3182_MOESM4_ESM.tgz › FANS_images/1A/CEMBA180227-1A.pdf]

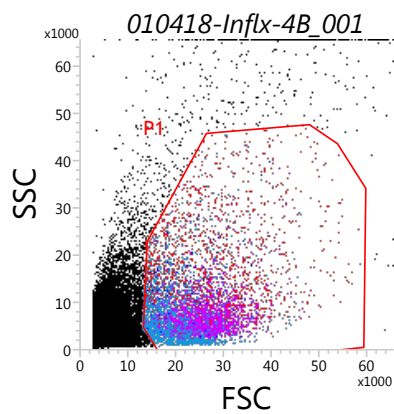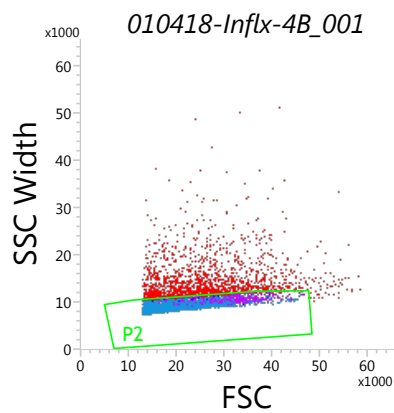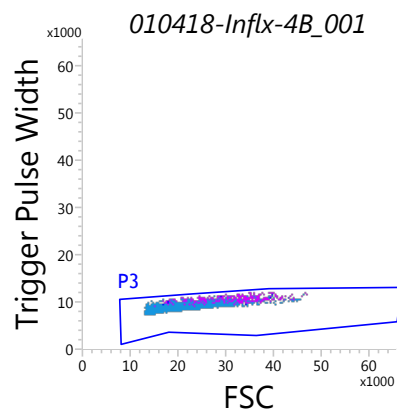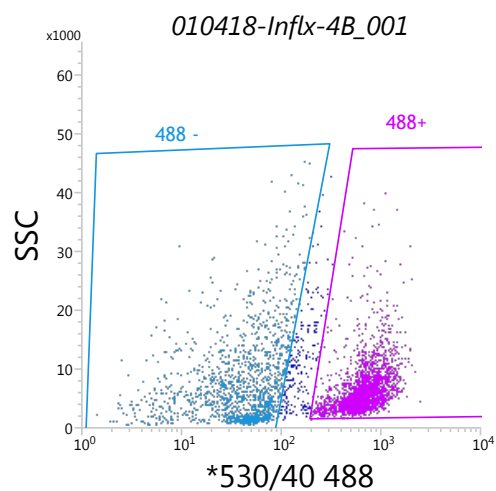

Populations: 010418-Inflx-4B\_001

| Populations | Events | % Total | % Parent |
|-------------|--------|---------|----------|
| All Events  | 27,173 | 100.00% | ####     |
| P1          | 5,663  | 20.84%  | 20.84%   |
| P2          | 4,014  | 14.77%  | 70.88%   |
| P3          | 4,014  | 14.77%  | 100.00%  |
| 488+        | 2,318  | 8.53%   | 57.75%   |
| 488 -       | 1,494  | 5.50%   | 37.22%   |

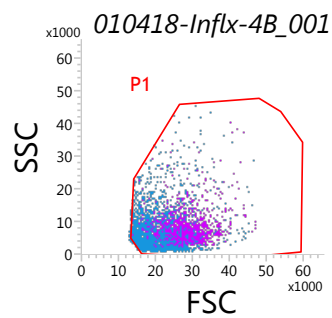

Supplement: Supplementary file 4 — This Tar/Gzip compressed file contains FANS images and gating strategies of the nuclei preparation experiments in this study. [file 41586_2020_3182_MOESM4_ESM.tgz › FANS_images/4B/CEMBA180104-4B.pdf]

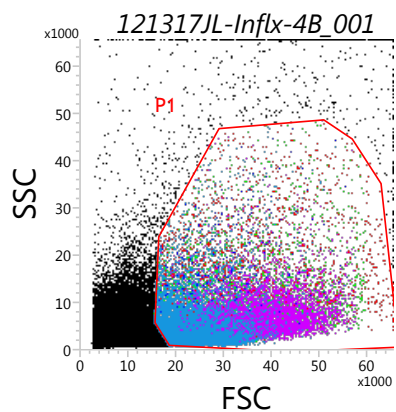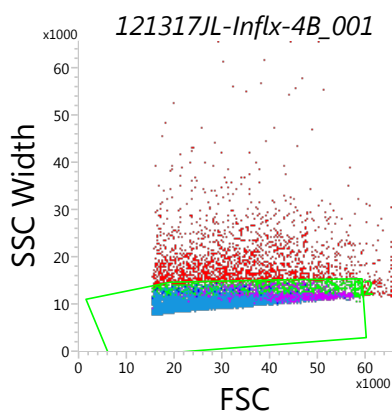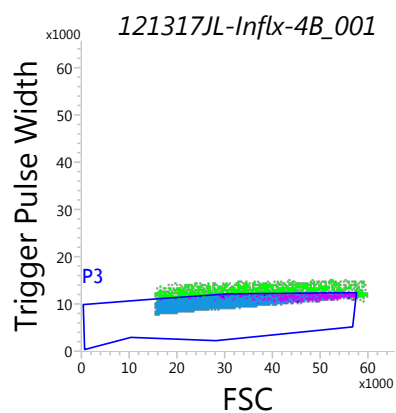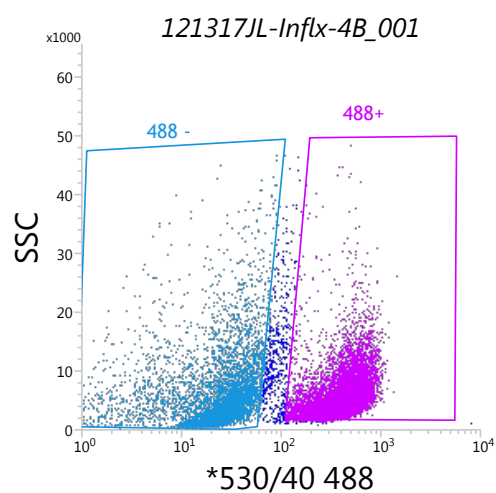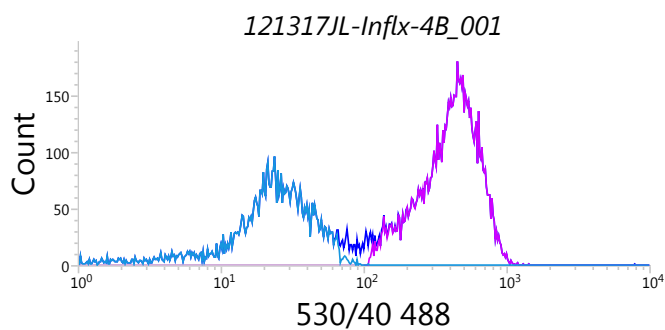

Populations: 121317JL-Inflx-4B\_001

| Populations | Events | % Total | % Parent |
|-------------|--------|---------|----------|
| All Events  | 86,378 | 100.00% | ####     |
| P1          | 19,128 | 22.14%  | 22.14%   |
| P2          | 17,027 | 19.71%  | 89.02%   |
| P3          | 15,253 | 17.66%  | 89.58%   |
| 488+        | 8,988  | 10.41%  | 58.93%   |
| 488 -       | 5,701  | 6.60%   | 37.38%   |

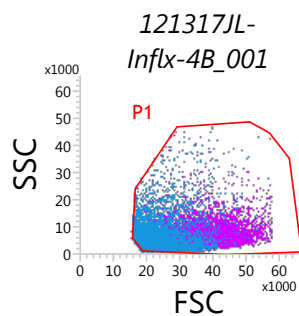

Supplement: Supplementary file 4 — This Tar/Gzip compressed file contains FANS images and gating strategies of the nuclei preparation experiments in this study. [file 41586_2020_3182_MOESM4_ESM.tgz › FANS_images/4B/CEMBA171213-4B.pdf]

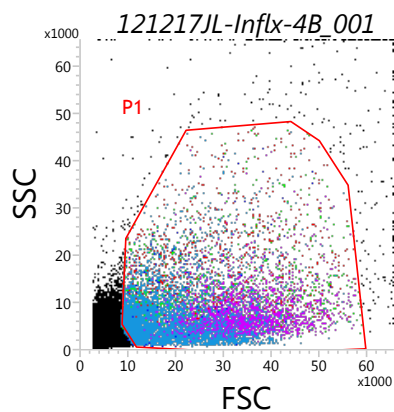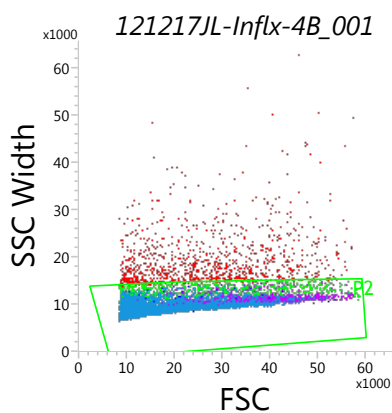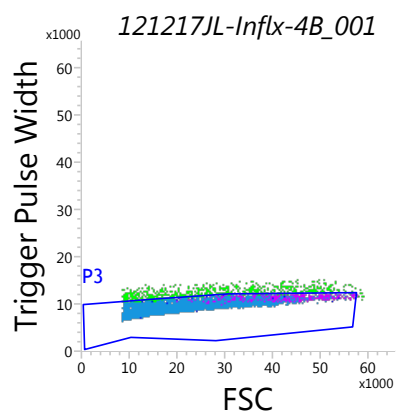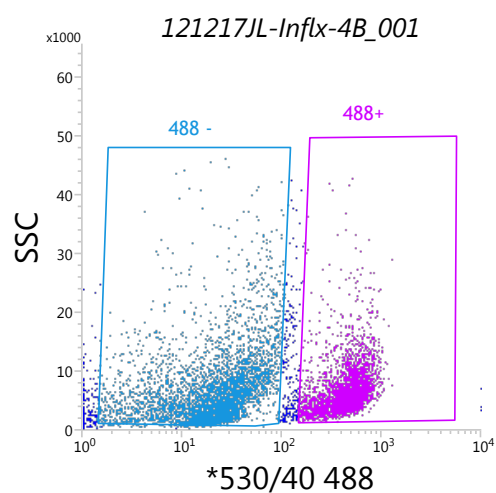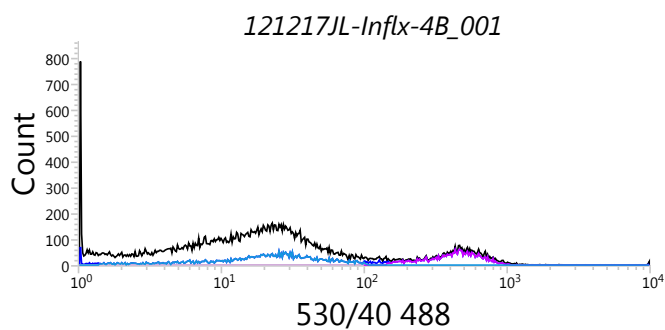

Populations: 121217JL-Inflx-4B\_001

| Populations | Events | % Total | % Parent |
|-------------|--------|---------|----------|
| All Events  | 24,386 | 100.00% | ####     |
| P1          | 9,052  | 37.12%  | 37.12%   |
| P2          | 8,036  | 32.95%  | 88.78%   |
| P3          | 7,294  | 29.91%  | 90.77%   |
| 488+        | 2,714  | 11.13%  | 37.21%   |
| 488 -       | 4,194  | 17.20%  | 57.50%   |

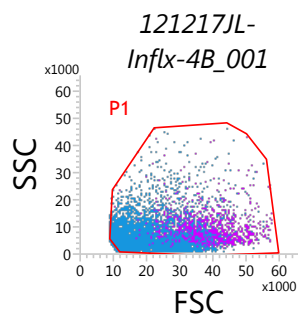

Supplement: Supplementary file 4 — This Tar/Gzip compressed file contains FANS images and gating strategies of the nuclei preparation experiments in this study. [file 41586_2020_3182_MOESM4_ESM.tgz › FANS_images/4B/CEMBA171212-4B.pdf]

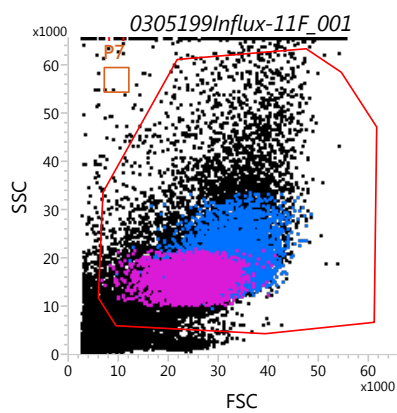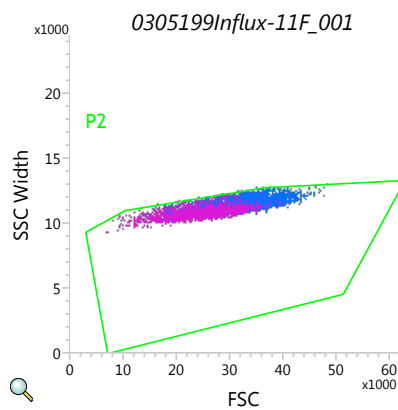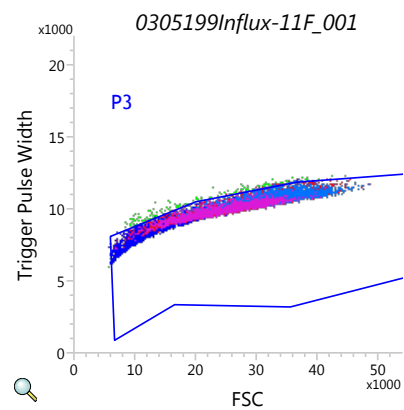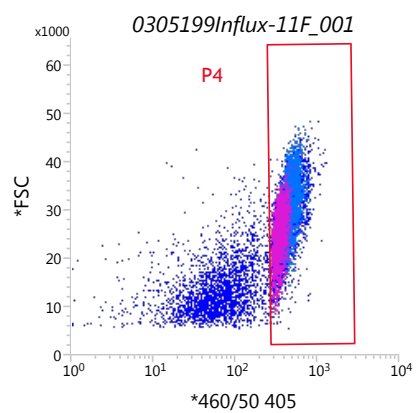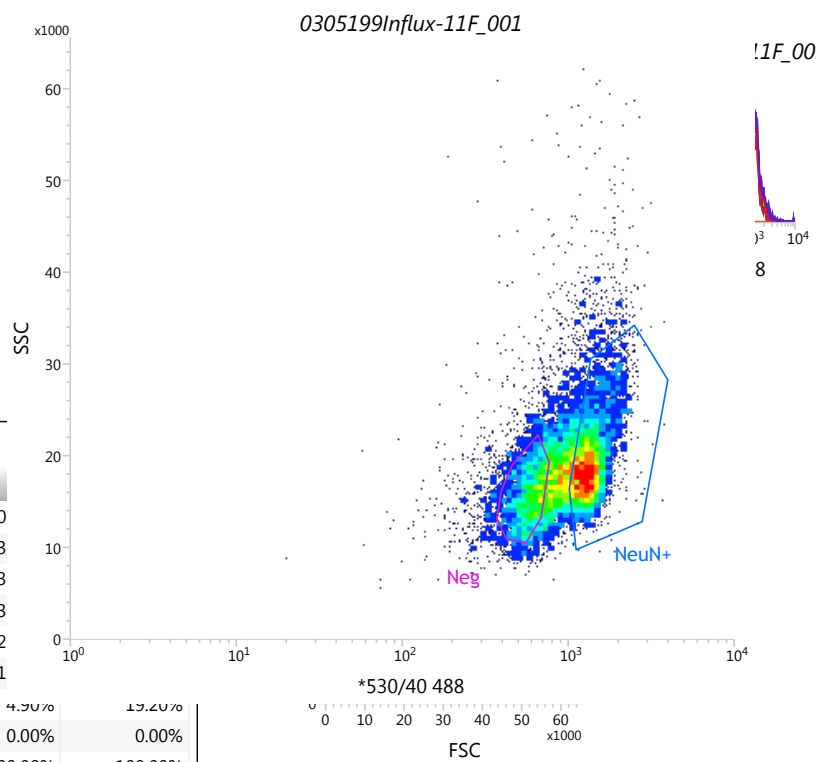

Populations: 0305199Influx-11F\_001

| Populations | Events | % Total |         |
|-------------|--------|---------|---------|
| All Events  | 50,000 | 10      |         |
| P1          | 19,724 | 3       |         |
| P2          | 15,436 | 3       |         |
| P3          | 15,251 | 3       |         |
| P4          | 12,757 | 2       |         |
| NeuN+       | 5,659  | 1       |         |
| Neg         | 2,449  | 4.90%   | 19.20%  |
| P7          | 2      | 0.00%   | 0.00%   |
| NOT(P7)     | 49,998 | 100.00% | 100.00% |

Supplement: Supplementary file 4 — This Tar/Gzip compressed file contains FANS images and gating strategies of the nuclei preparation experiments in this study. [file 41586_2020_3182_MOESM4_ESM.tgz › FANS_images/11F/CEMBA190305-11F.pdf]

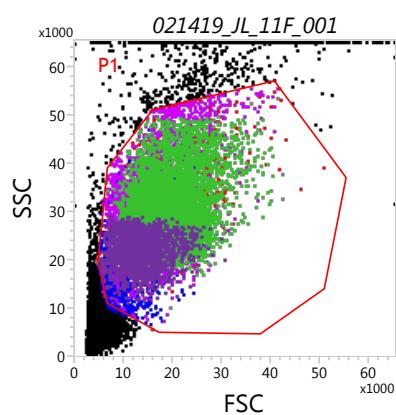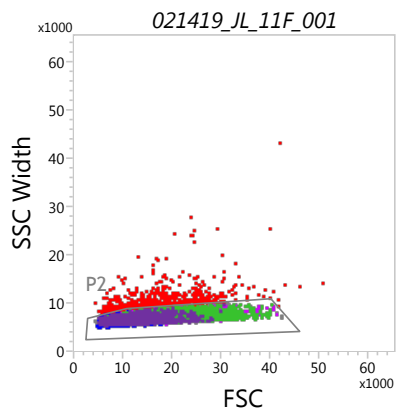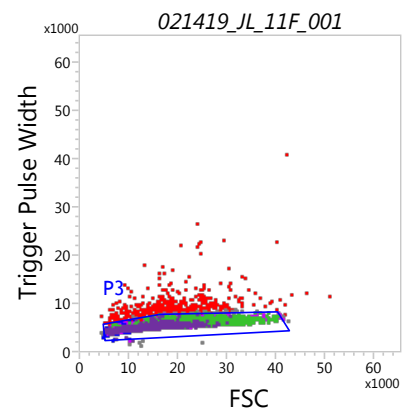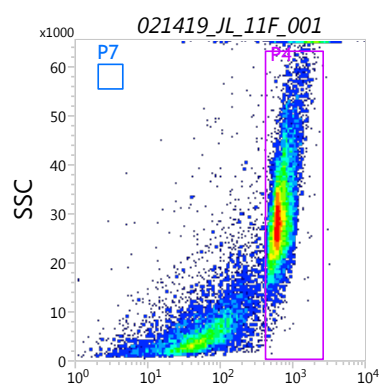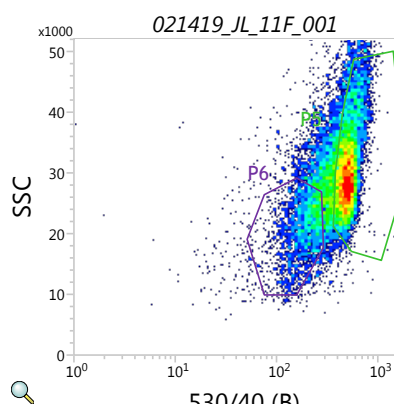

021419\_JL\_11F\_001

Statistics: 021419\_JL\_11F\_001

Populations

All Events

P1

P2

P3

P4

P5

P6

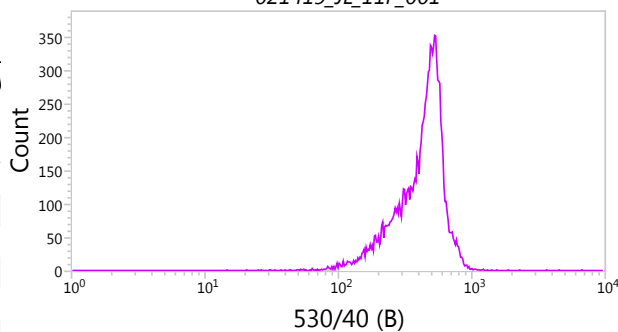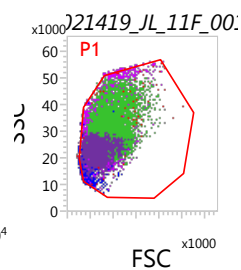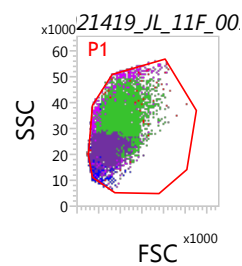

|       |       |        |
|-------|-------|--------|
| 1,721 | 8.60% | 14.12% |
|-------|-------|--------|

Supplement: Supplementary file 4 — This Tar/Gzip compressed file contains FANS images and gating strategies of the nuclei preparation experiments in this study. [file 41586_2020_3182_MOESM4_ESM.tgz › FANS_images/11F/CEMBA190214-11F.pdf]

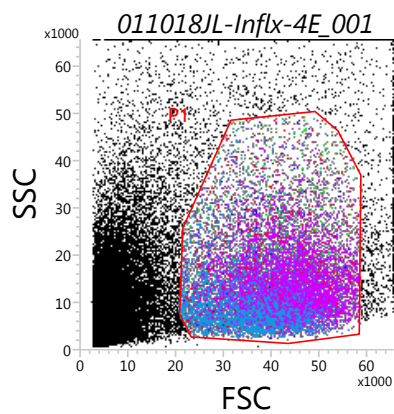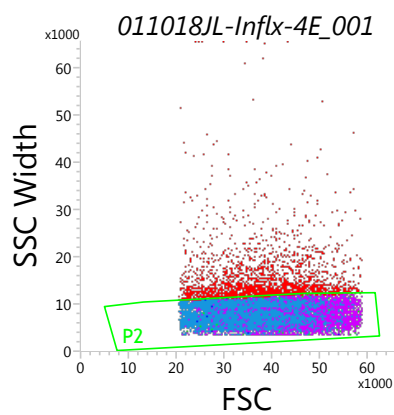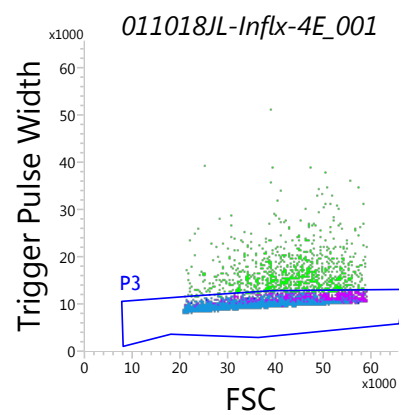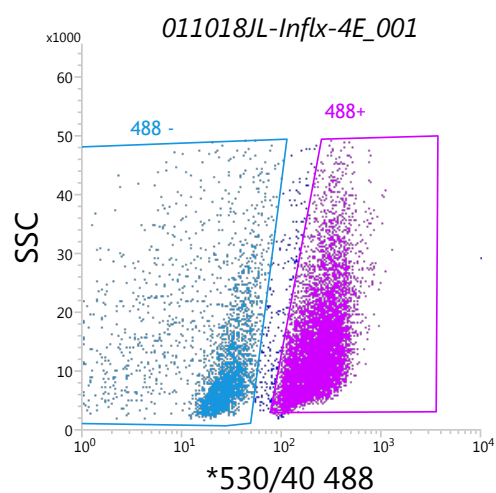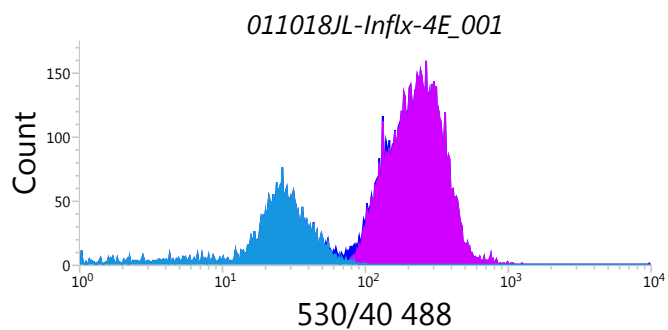

Populations: 011018JL-Inflx-4E\_001

| Populations | Events | % Total | % Parent |
|-------------|--------|---------|----------|
| All Events  | 48,117 | 100.00% | ####     |
| P1          | 15,470 | 32.15%  | 32.15%   |
| P2          | 13,193 | 27.42%  | 85.28%   |
| P3          | 12,111 | 25.17%  | 91.80%   |
| 488+        | 8,686  | 18.05%  | 71.72%   |
| 488 -       | 3,197  | 6.64%   | 26.40%   |

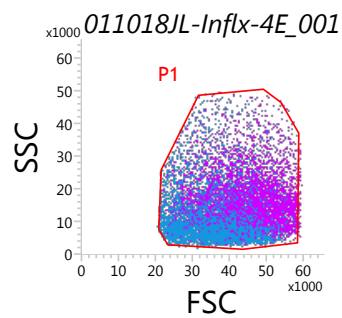

Supplement: Supplementary file 4 — This Tar/Gzip compressed file contains FANS images and gating strategies of the nuclei preparation experiments in this study. [file 41586_2020_3182_MOESM4_ESM.tgz › FANS_images/4E/CEMBA180110-4E.pdf]

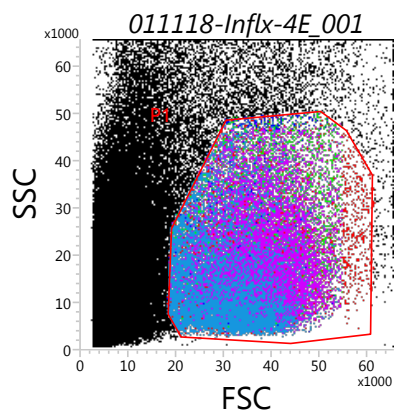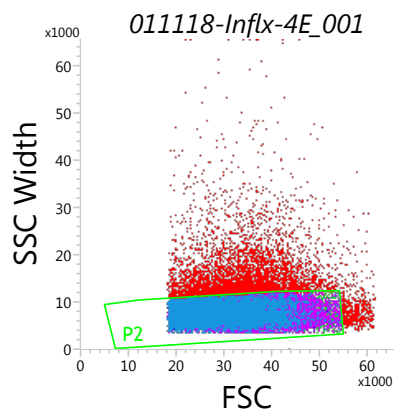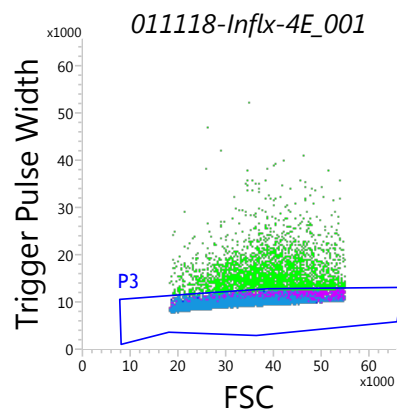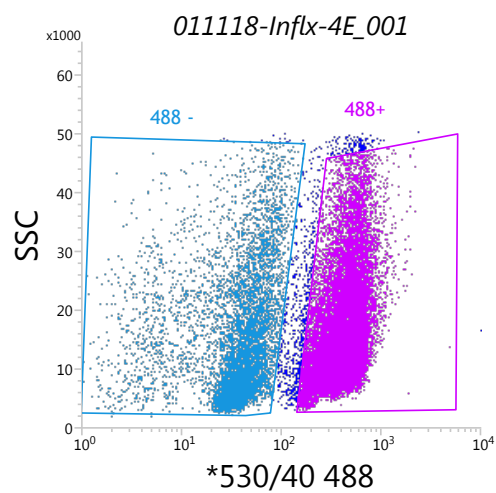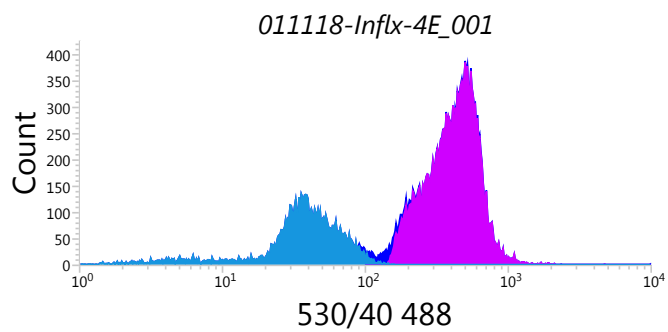

Populations: 011118-Inflx-4E\_001

| Populations | Events  | % Total | % Parent |
|-------------|---------|---------|----------|
| All Events  | 190,127 | 100.00% | ####     |
| P1          | 35,458  | 18.65%  | 18.65%   |
| P2          | 29,838  | 15.69%  | 84.15%   |
| P3          | 26,582  | 13.98%  | 89.09%   |
| 488+        | 18,229  | 9.59%   | 68.58%   |
| 488 -       | 7,544   | 3.97%   | 28.38%   |

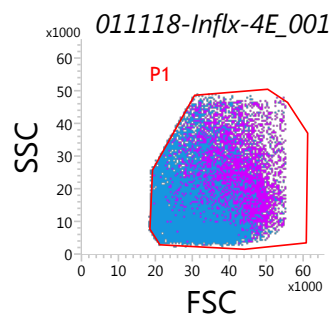

Supplement: Supplementary file 4 — This Tar/Gzip compressed file contains FANS images and gating strategies of the nuclei preparation experiments in this study. [file 41586_2020_3182_MOESM4_ESM.tgz › FANS_images/4E/CEMBA180111-4E.pdf]

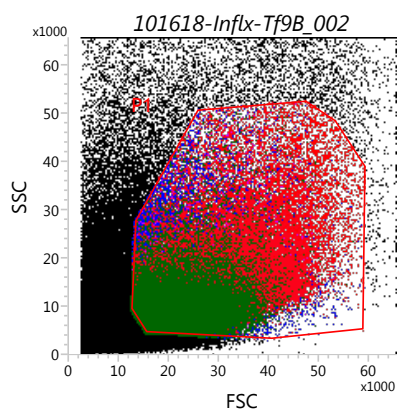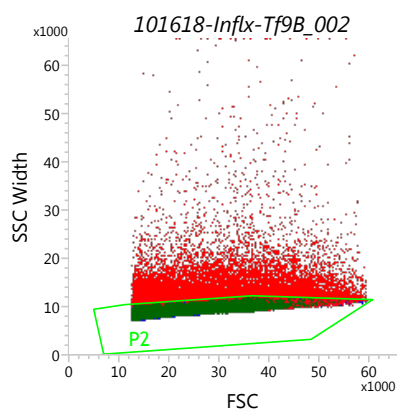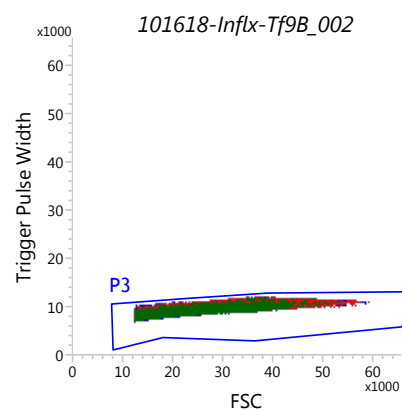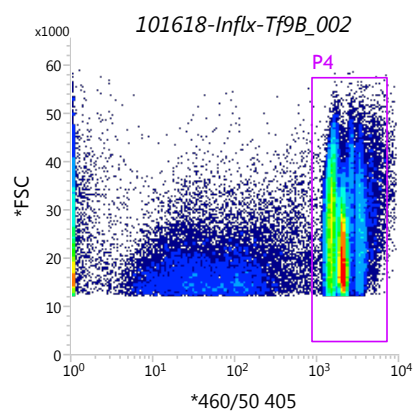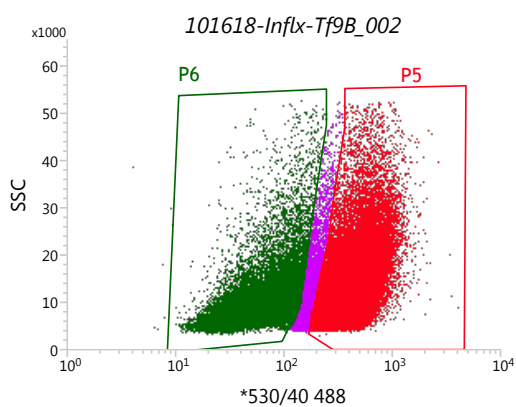

Populations: 101618-Inflx-Tf9B\_002

| Populations | Events  | % Total | % Parent |
|-------------|---------|---------|----------|
| All Events  | 545,150 | 100.00% | ####     |
| P1          | 134,911 | 24.75%  | 24.75%   |
| P2          | 120,041 | 22.02%  | 88.98%   |
| P3          | 120,041 | 22.02%  | 100.00%  |
| P4          | 95,955  | 17.60%  | 79.94%   |
| P5          | 62,095  | 11.39%  | 64.71%   |
| P6          | 27,882  | 5.11%   | 29.06%   |

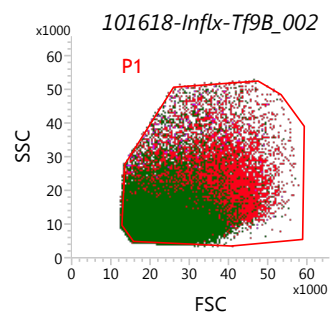

Supplement: Supplementary file 4 — This Tar/Gzip compressed file contains FANS images and gating strategies of the nuclei preparation experiments in this study. [file 41586_2020_3182_MOESM4_ESM.tgz › FANS_images/5H/CEMBA181016-5H.pdf]

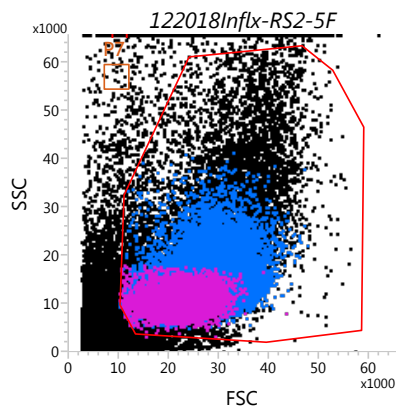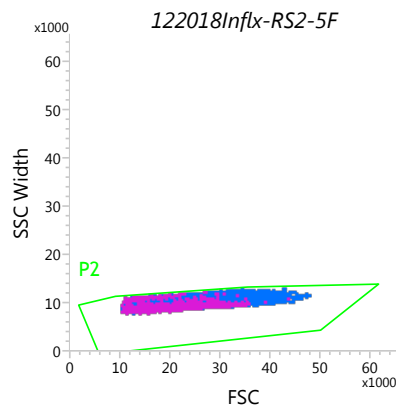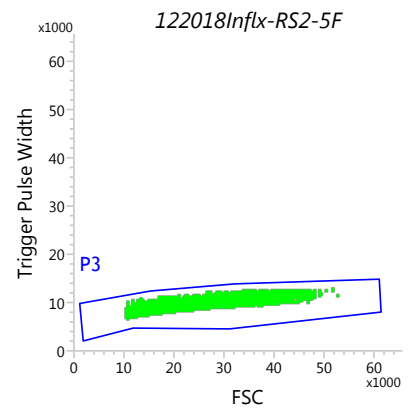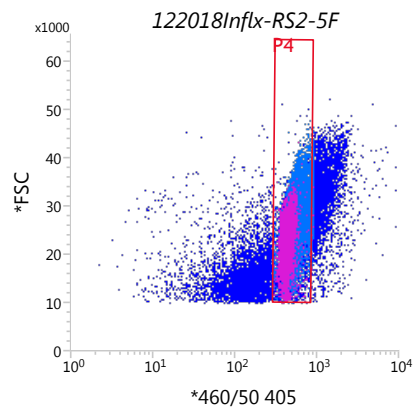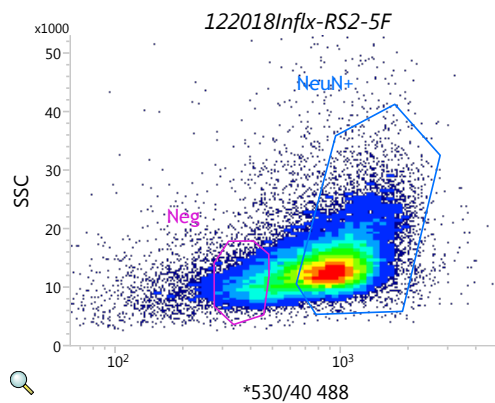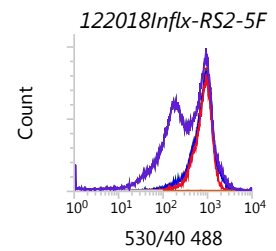

Populations: 122018Inflx-RS2-5F

| Populations | Events  | % Total | % Parent |
|-------------|---------|---------|----------|
| All Events  | 107,602 | 100.00% | ####     |
| P1          | 49,790  | 46.27%  | 46.27%   |
| P2          | 47,576  | 44.21%  | 95.55%   |
| P3          | 47,576  | 44.21%  | 100.00%  |
| P4          | 39,721  | 36.91%  | 83.49%   |
| NeuN+       | 27,825  | 25.86%  | 70.05%   |
| Neg         | 4,125   | 3.83%   | 10.38%   |
| P7          | 12      | 0.01%   | 0.01%    |
| NOT(P7)     | 107,590 | 99.99%  | 99.99%   |

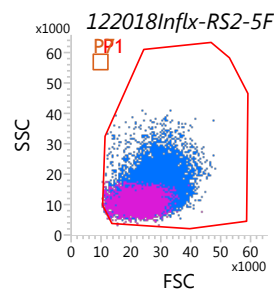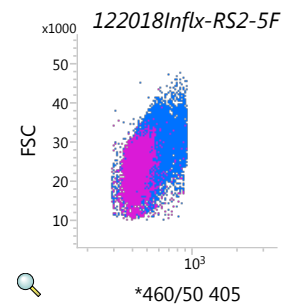

Supplement: Supplementary file 4 — This Tar/Gzip compressed file contains FANS images and gating strategies of the nuclei preparation experiments in this study. [file 41586_2020_3182_MOESM4_ESM.tgz › FANS_images/5F/CEMBA181220-5F.pdf]

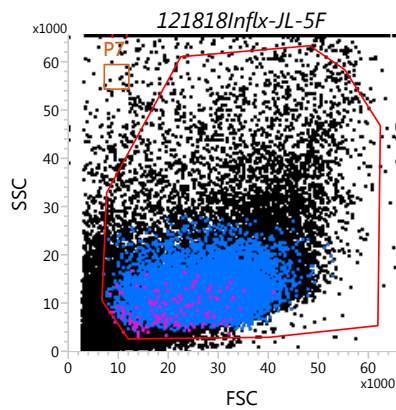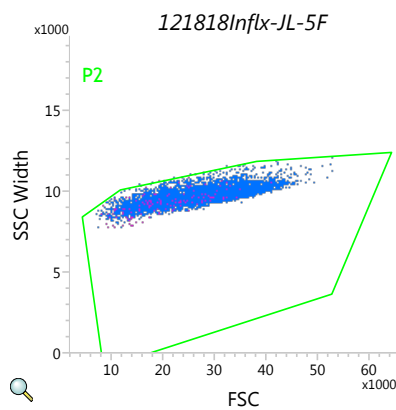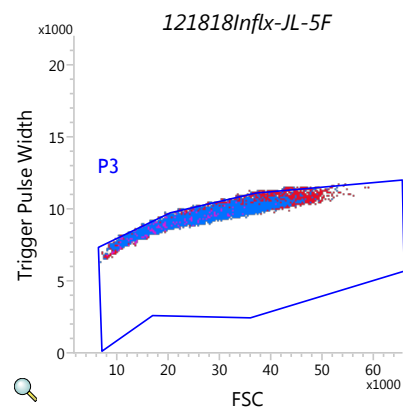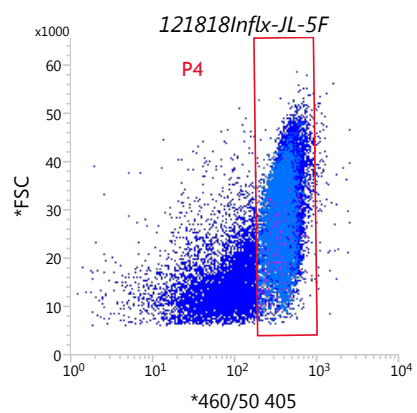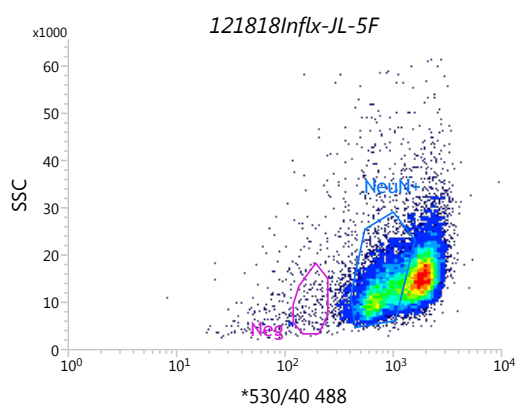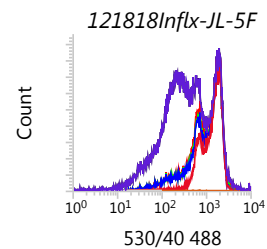

Populations: 121818Inflx-JL-5F

| Populations | Events | % Total | % Parent |
|-------------|--------|---------|----------|
| All Events  | 64,404 | 100.00% | ####     |
| P1          | 30,405 | 47.21%  | 47.21%   |
| P2          | 27,424 | 42.58%  | 90.20%   |
| P3          | 26,450 | 41.07%  | 96.45%   |
| P4          | 19,930 | 30.95%  | 75.35%   |
| NeuN+       | 7,436  | 11.55%  | 37.31%   |
| Neg         | 126    | 0.20%   | 0.63%    |
| P7          | 9      | 0.01%   | 0.01%    |
| NOT(P7)     | 64,395 | 99.99%  | 99.99%   |

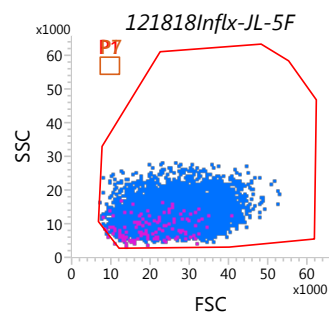

Supplement: Supplementary file 4 — This Tar/Gzip compressed file contains FANS images and gating strategies of the nuclei preparation experiments in this study. [file 41586_2020_3182_MOESM4_ESM.tgz › FANS_images/5F/CEMBA181218-5F.pdf]

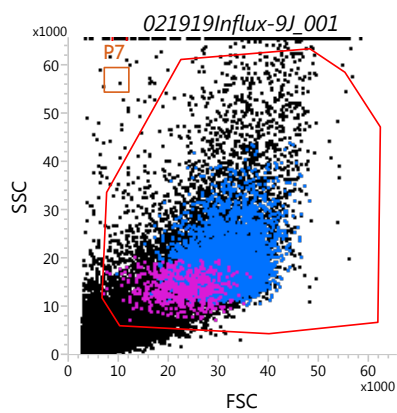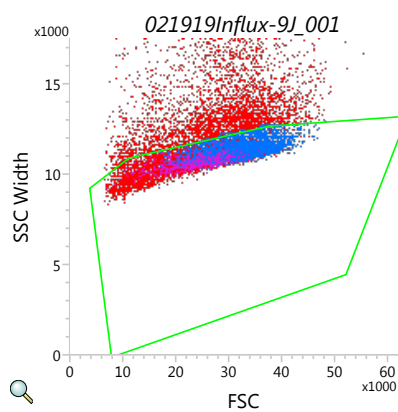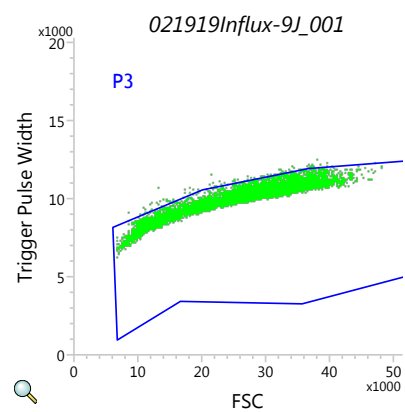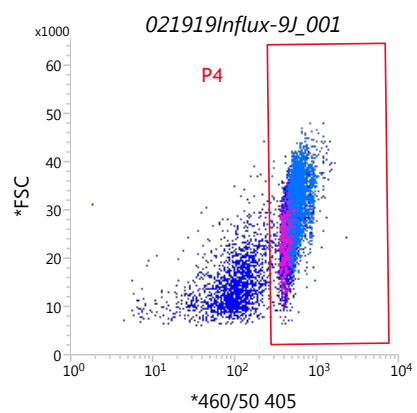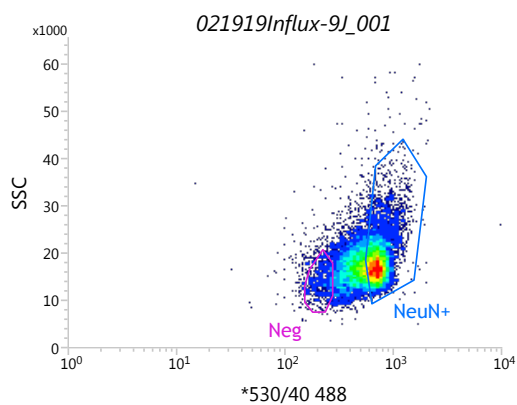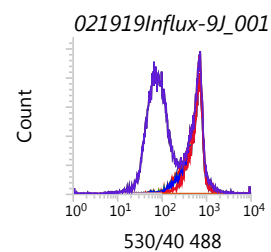

Populations: 021919Influx-9J\_001

| Populations | Events | % Total | % Parent |
|-------------|--------|---------|----------|
| All Events  | 50,000 | 100.00% | ####     |
| P1          | 19,962 | 39.92%  | 39.92%   |
| P2          | 15,855 | 31.71%  | 79.43%   |
| P3          | 15,791 | 31.58%  | 99.60%   |
| P4          | 14,104 | 28.21%  | 89.32%   |
| NeuN+       | 8,354  | 16.71%  | 59.23%   |
| Neg         | 561    | 1.12%   | 3.98%    |
| P7          | 1      | 0.00%   | 0.00%    |
| NOT(P7)     | 49,999 | 100.00% | 100.00%  |

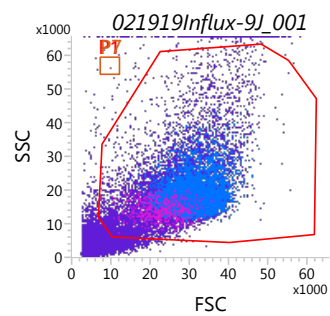

Supplement: Supplementary file 4 — This Tar/Gzip compressed file contains FANS images and gating strategies of the nuclei preparation experiments in this study. [file 41586_2020_3182_MOESM4_ESM.tgz › FANS_images/9J/CEMBA190219-9J.pdf]

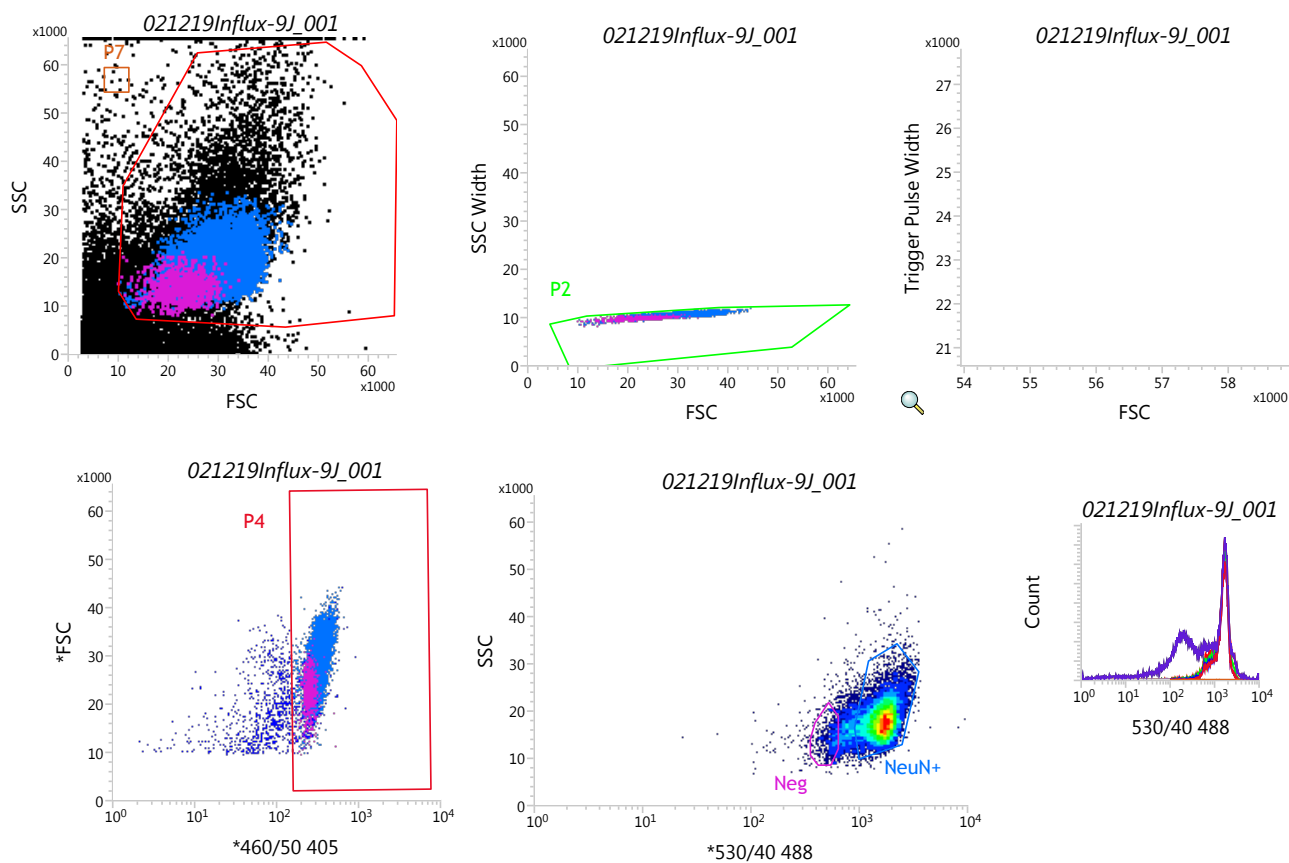

Populations: 021219Influx-9J\_001

| Populations | Events | % Total | % Parent |
|-------------|--------|---------|----------|
| All Events  | 50,000 | 100.00% | ####     |
| P1          | 26,503 | 53.01%  | 53.01%   |
| P2          | 22,183 | 44.37%  | 83.70%   |
| P3          | 19,033 | 38.07%  | 85.80%   |
| P4          | 18,281 | 36.56%  | 96.05%   |
| NeuN+       | 15,700 | 31.40%  | 85.88%   |
| Neg         | 779    | 1.56%   | 4.26%    |
| P7          | 7      | 0.01%   | 0.01%    |
| NOT(P7)     | 49,993 | 99.99%  | 99.99%   |

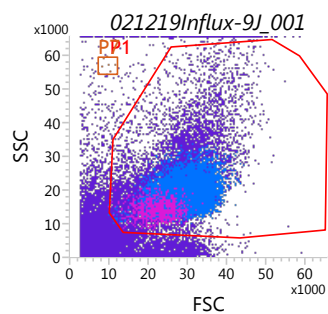

Supplement: Supplementary file 4 — This Tar/Gzip compressed file contains FANS images and gating strategies of the nuclei preparation experiments in this study. [file 41586_2020_3182_MOESM4_ESM.tgz › FANS_images/9J/CEMBA190212-9J.pdf]

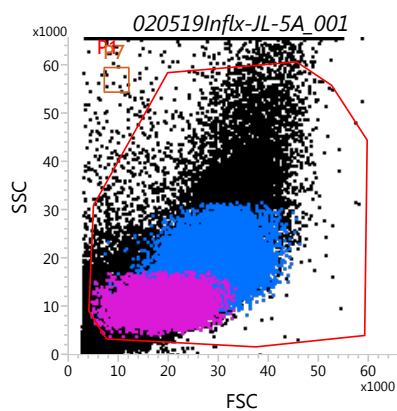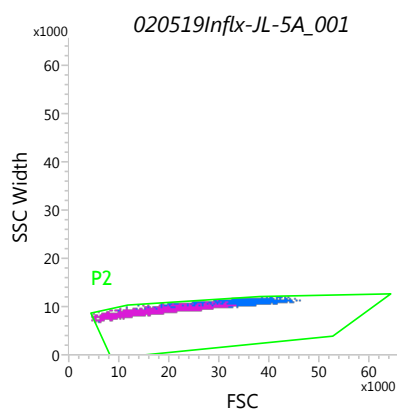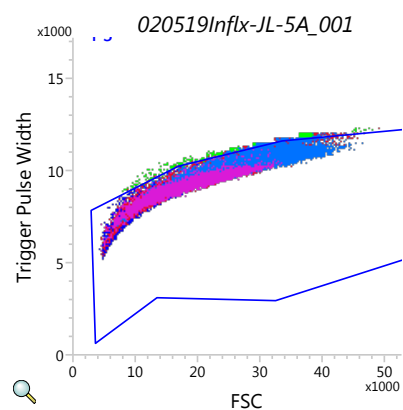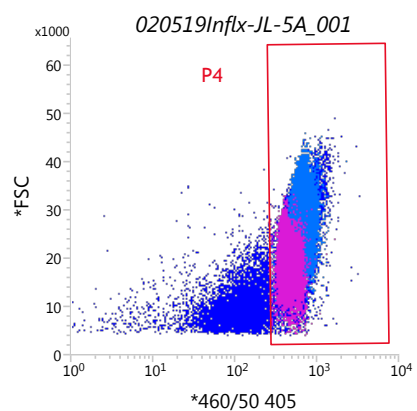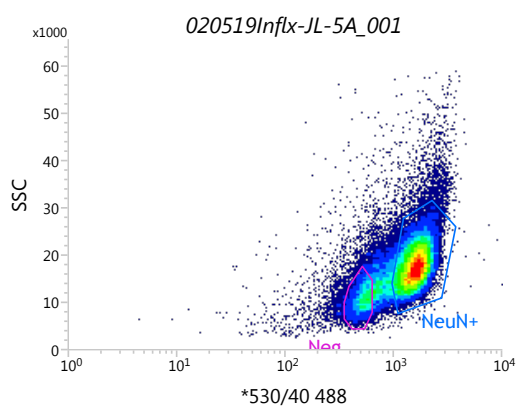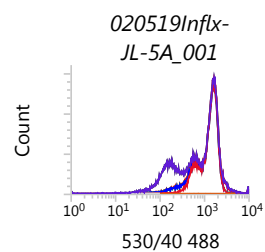

Populations: 020519Inflx-JL-5A\_001

| Populations | Events  | % Total | % Parent |
|-------------|---------|---------|----------|
| All Events  | 100,000 | 100.00% | ####     |
| P1          | 74,426  | 74.43%  | 74.43%   |
| P2          | 67,399  | 67.40%  | 90.56%   |
| P3          | 66,937  | 66.94%  | 99.31%   |
| P4          | 60,578  | 60.58%  | 90.50%   |
| NeuN+       | 41,876  | 41.88%  | 69.13%   |
| Neg         | 6,868   | 6.87%   | 11.34%   |
| P7          | 9       | 0.01%   | 0.01%    |
| NOT(P7)     | 99,991  | 99.99%  | 99.99%   |

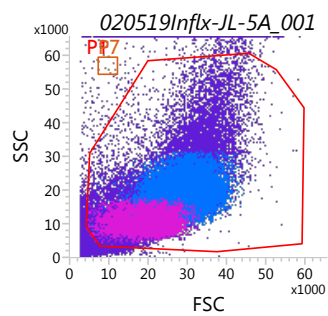

Supplement: Supplementary file 4 — This Tar/Gzip compressed file contains FANS images and gating strategies of the nuclei preparation experiments in this study. [file 41586_2020_3182_MOESM4_ESM.tgz › FANS_images/5A/CEMBA190205-5A.pdf]

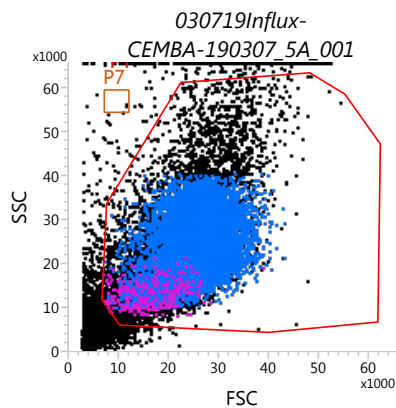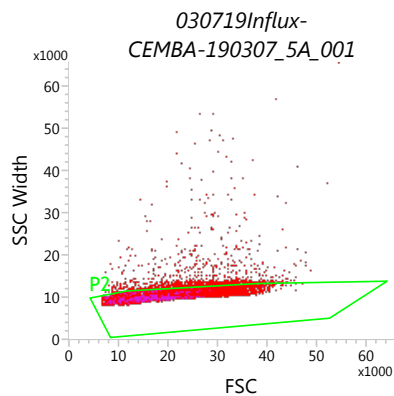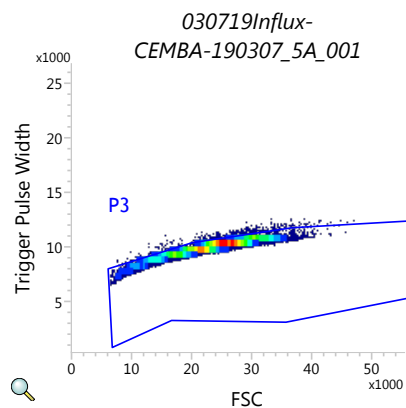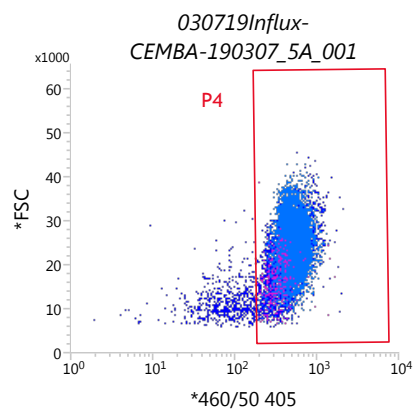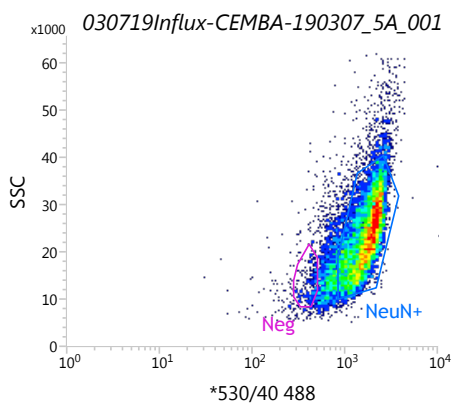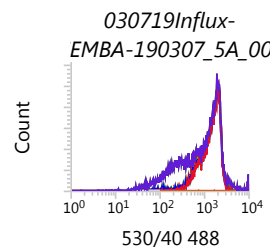

Populations: 030719Influx-CEMBA-190307\_5A\_001

| Populations | Events | % Total | % Parent |
|-------------|--------|---------|----------|
| All Events  | 20,000 | 100.00% | ####     |
| P1          | 13,648 | 68.24%  | 68.24%   |
| P2          | 12,197 | 60.98%  | 89.37%   |
| P3          | 11,940 | 59.70%  | 97.89%   |
| P4          | 11,308 | 56.54%  | 94.71%   |
| NeuN+       | 8,566  | 42.83%  | 75.75%   |
| Neg         | 266    | 1.33%   | 2.35%    |
| P7          | 2      | 0.01%   | 0.01%    |
| NOT(P7)     | 19,998 | 99.99%  | 99.99%   |

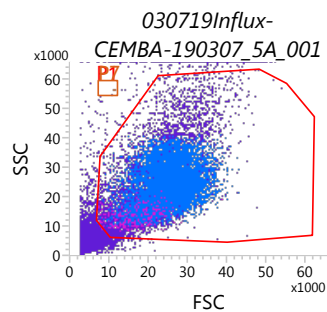

Supplement: Supplementary file 4 — This Tar/Gzip compressed file contains FANS images and gating strategies of the nuclei preparation experiments in this study. [file 41586_2020_3182_MOESM4_ESM.tgz › FANS_images/5A/CEMBA190307-5A.pdf]

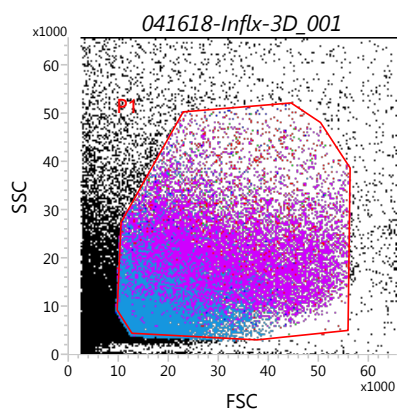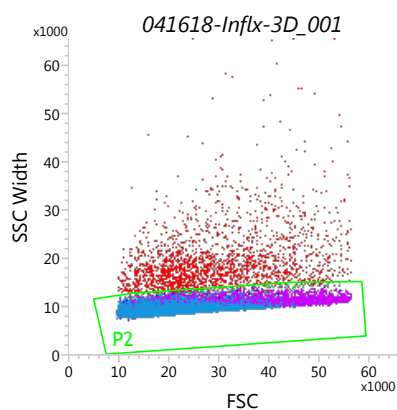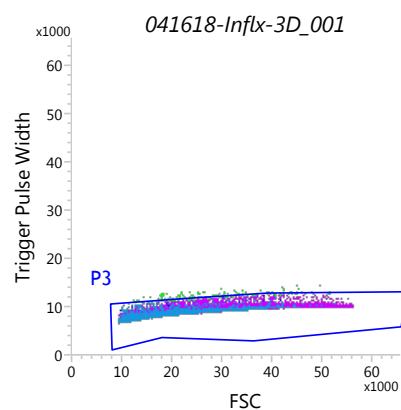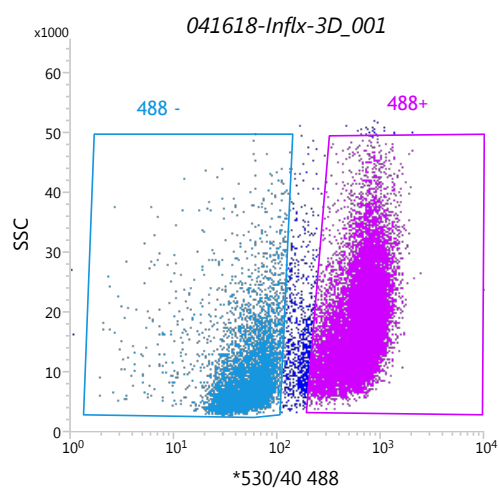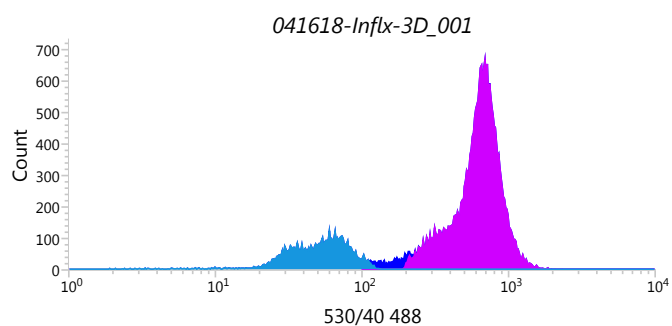

Populations: 041618-Inflx-3D\_001

| Populations | Events | % Total | % Parent |
|-------------|--------|---------|----------|
| All Events  | 51,125 | 100.00% | ####     |
| P1          | 32,688 | 63.94%  | 63.94%   |
| P2          | 30,466 | 59.59%  | 93.20%   |
| P3          | 30,285 | 59.24%  | 99.41%   |
| 488+        | 23,373 | 45.72%  | 77.18%   |
| 488 -       | 6,031  | 11.80%  | 19.91%   |

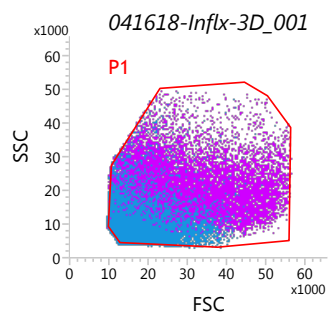

Supplement: Supplementary file 4 — This Tar/Gzip compressed file contains FANS images and gating strategies of the nuclei preparation experiments in this study. [file 41586_2020_3182_MOESM4_ESM.tgz › FANS_images/3D/CEMBA180416-3D.pdf]

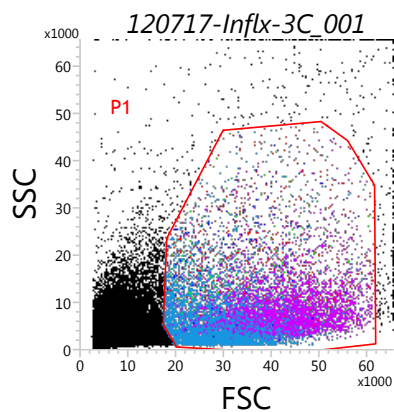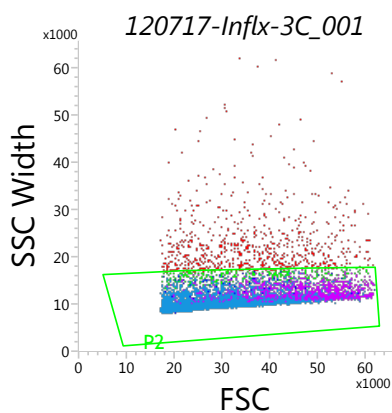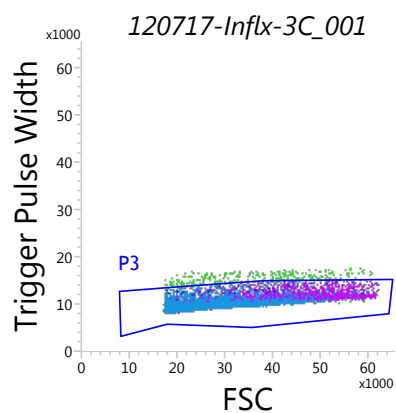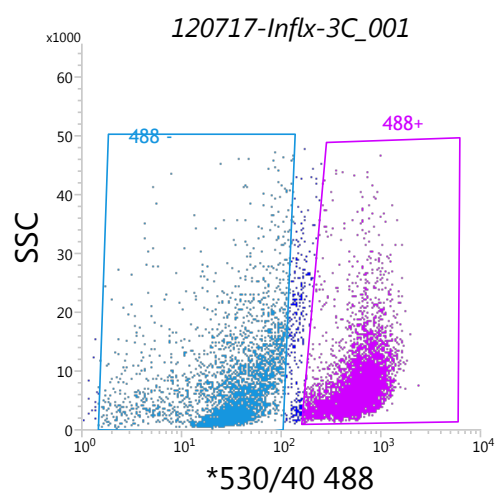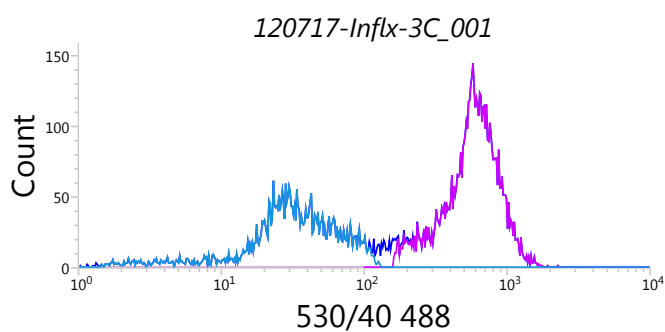

Populations: 120717-Inflx-3C\_001

| Populations | Events | % Total | % Parent |
|-------------|--------|---------|----------|
| All Events  | 45,579 | 100.00% | ####     |
| P1          | 11,297 | 24.79%  | 24.79%   |
| P2          | 10,541 | 23.13%  | 93.31%   |
| P3          | 10,231 | 22.45%  | 97.06%   |
| 488+        | 6,018  | 13.20%  | 58.82%   |
| 488-        | 3,878  | 8.51%   | 37.90%   |

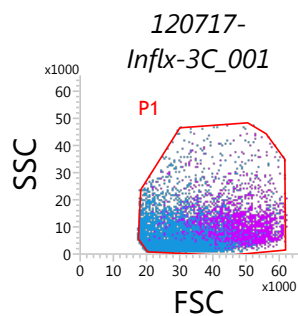

Supplement: Supplementary file 4 — This Tar/Gzip compressed file contains FANS images and gating strategies of the nuclei preparation experiments in this study. [file 41586_2020_3182_MOESM4_ESM.tgz › FANS_images/3C/CEMBA171207-3C.pdf]

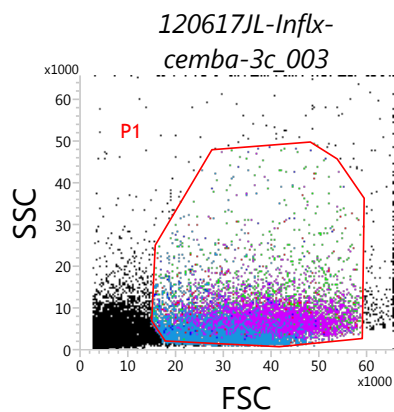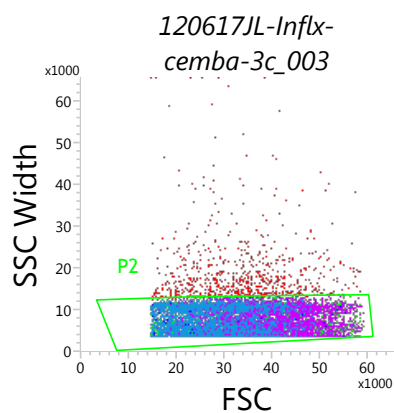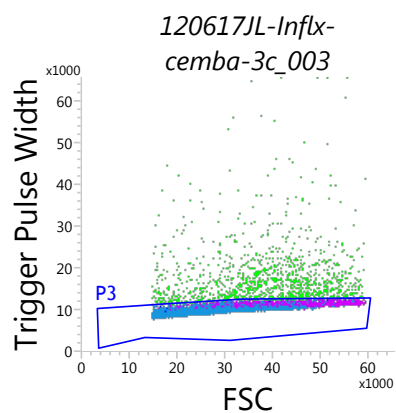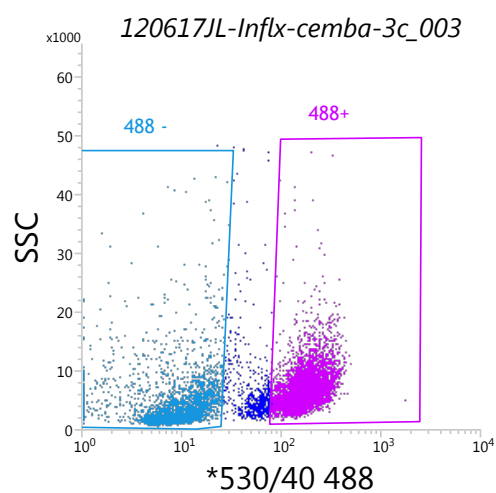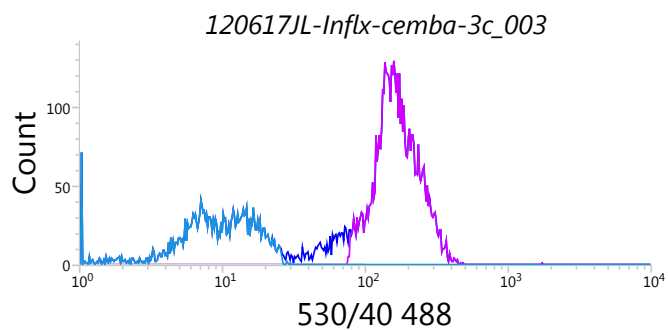

Populations: 120617JL-Inflx-cemba-3c\_003

| Populations | Events | % Total | % Parent |
|-------------|--------|---------|----------|
| All Events  | 27,154 | 100.00% | ####     |
| P1          | 10,457 | 38.51%  | 38.51%   |
| P2          | 9,732  | 35.84%  | 93.07%   |
| P3          | 8,485  | 31.25%  | 87.19%   |
| 488+        | 5,230  | 19.26%  | 61.64%   |
| 488 -       | 2,644  | 9.74%   | 31.16%   |

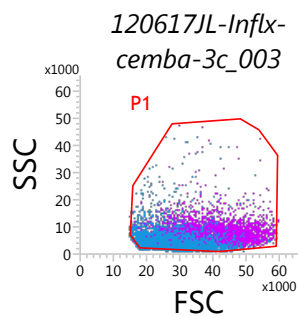

Supplement: Supplementary file 4 — This Tar/Gzip compressed file contains FANS images and gating strategies of the nuclei preparation experiments in this study. [file 41586_2020_3182_MOESM4_ESM.tgz › FANS_images/3C/CEMBA171206-3C.pdf]

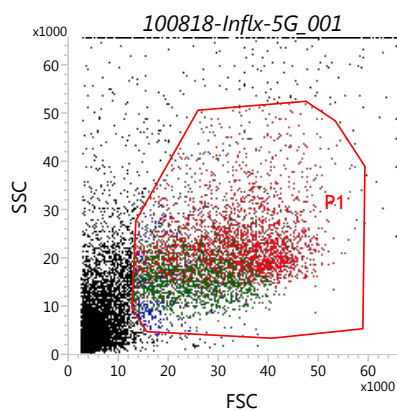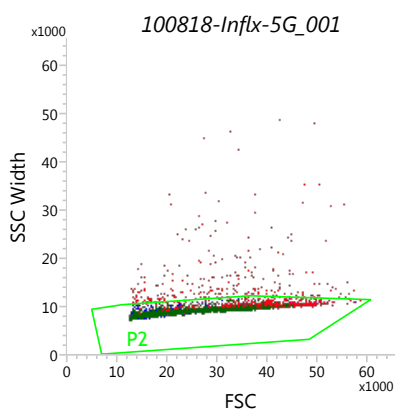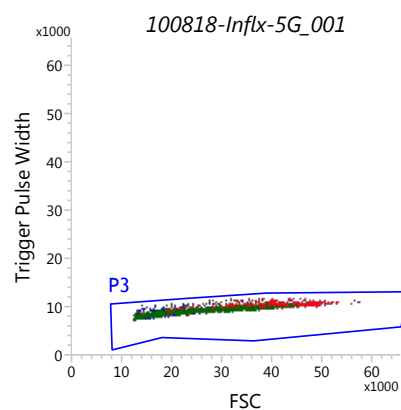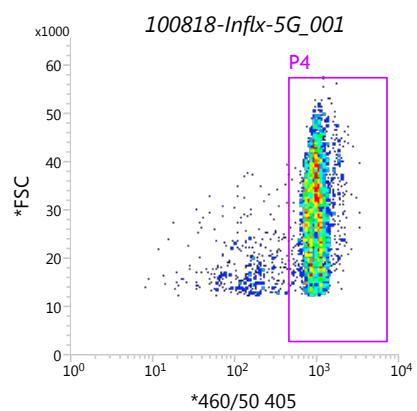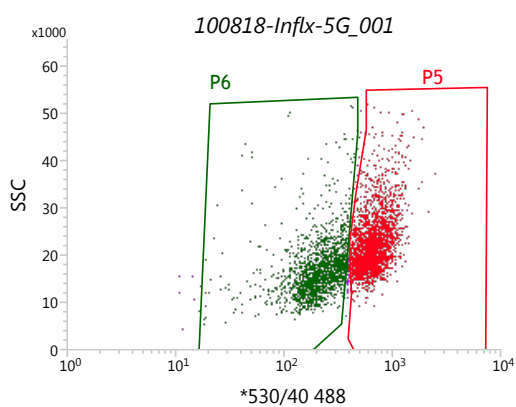

Populations: 100818-Inflx-5G\_001

| Populations | Events | % Total | % Parent |
|-------------|--------|---------|----------|
| All Events  | 12,754 | 100.00% | ####     |
| P1          | 4,845  | 37.99%  | 37.99%   |
| P2          | 4,557  | 35.73%  | 94.06%   |
| P3          | 4,557  | 35.73%  | 100.00%  |
| P4          | 4,203  | 32.95%  | 92.23%   |
| P5          | 2,479  | 19.44%  | 58.98%   |
| P6          | 1,663  | 13.04%  | 39.57%   |

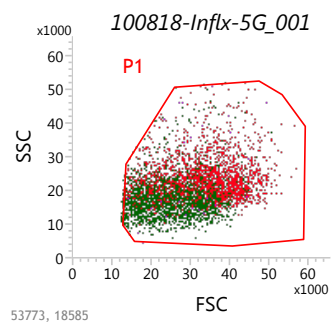

Supplement: Supplementary file 4 — This Tar/Gzip compressed file contains FANS images and gating strategies of the nuclei preparation experiments in this study. [file 41586_2020_3182_MOESM4_ESM.tgz › FANS_images/5G/CEMBA181008-5G.pdf]

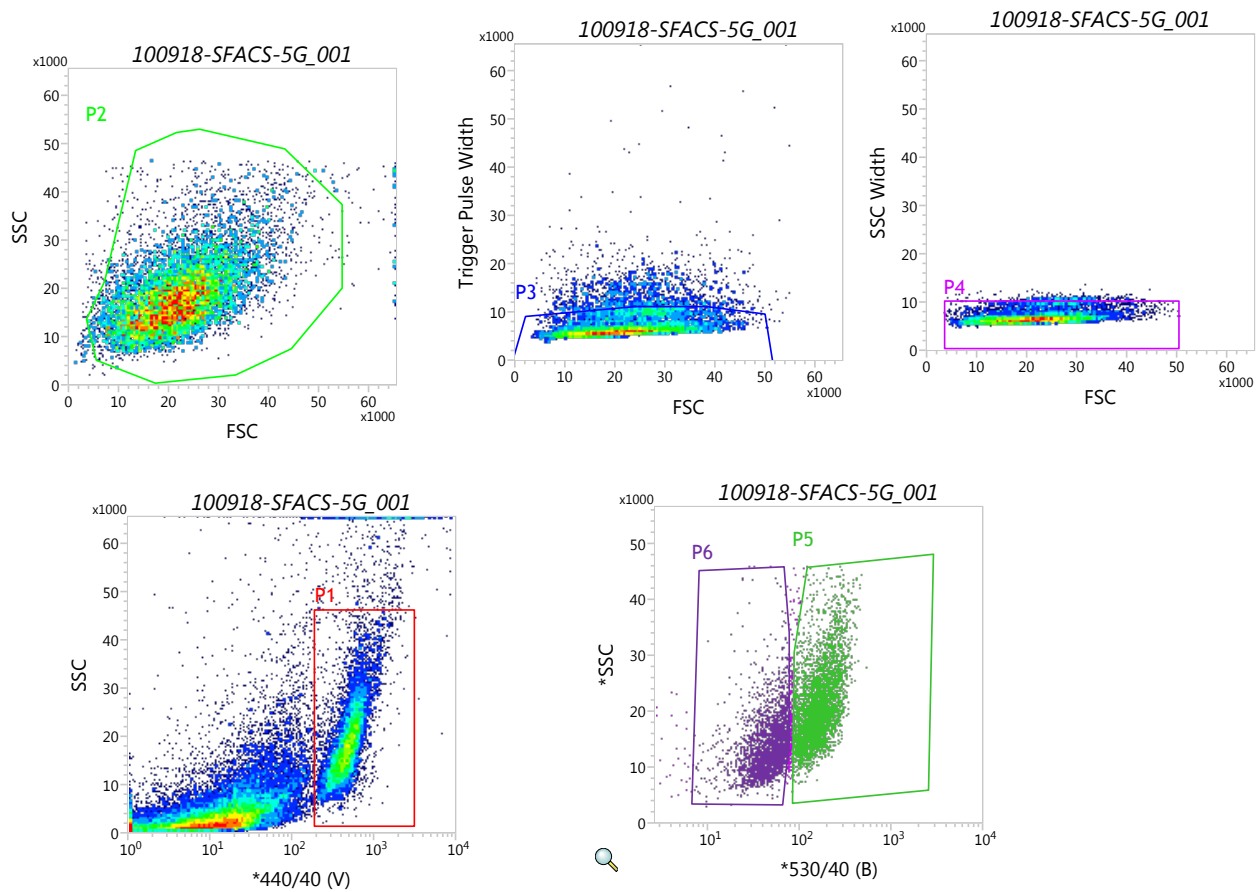

Populations: 100918-SFACS-5G\_001

| Populations | Even... | % Total | % Parent |
|-------------|---------|---------|----------|
| All Events  | 33,856  | 100.00% | ####     |
| P1          | 9,170   | 27.09%  | 27.09%   |
| P2          | 8,970   | 26.49%  | 97.82%   |
| P3          | 7,640   | 22.57%  | 85.17%   |
| P4          | 7,175   | 21.19%  | 93.91%   |
| P5          | 4,129   | 12.20%  | 57.55%   |
| P6          | 2,727   | 8.05%   | 38.01%   |

Supplement: Supplementary file 4 — This Tar/Gzip compressed file contains FANS images and gating strategies of the nuclei preparation experiments in this study. [file 41586_2020_3182_MOESM4_ESM.tgz › FANS_images/5G/CEMBA181009-5G.pdf]

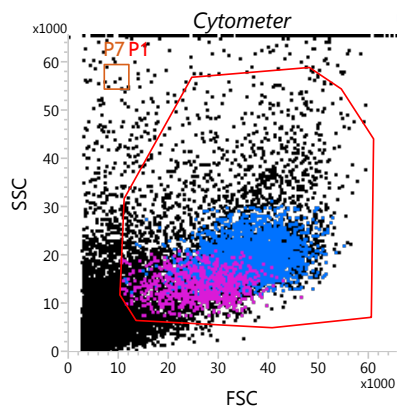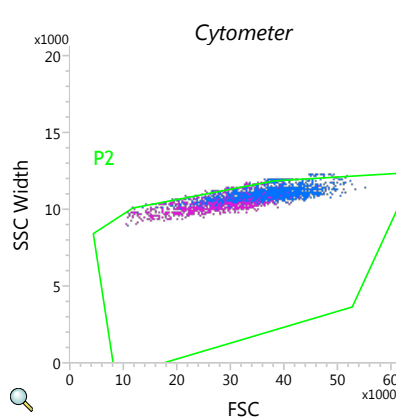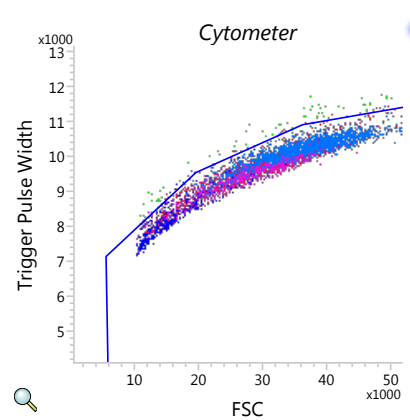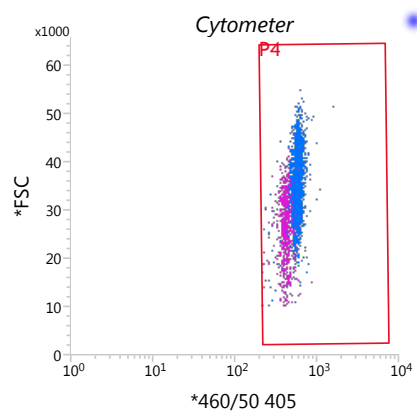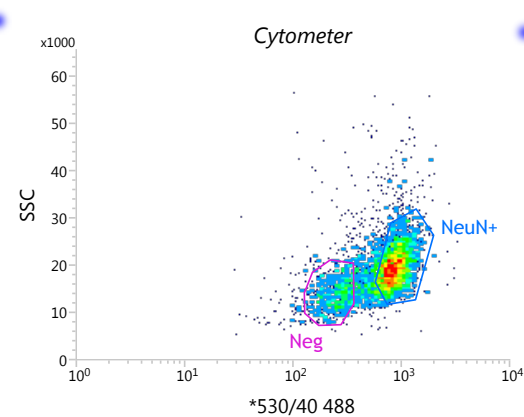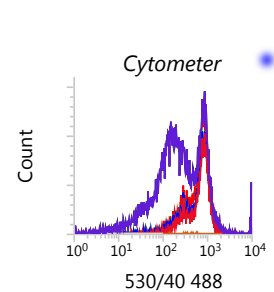

Populations: Cytometer

| Populations | Events | % Total | % Parent |
|-------------|--------|---------|----------|
| All Events  | 16,061 | 100.00% | ####     |
| P1          | 6,849  | 42.64%  | 42.64%   |
| P2          | 4,953  | 30.84%  | 72.32%   |
| P3          | 4,890  | 30.45%  | 98.73%   |
| P4          | 4,216  | 26.25%  | 86.22%   |
| NeuN+       | 2,604  | 16.21%  | 61.76%   |
| Neg         | 684    | 4.26%   | 16.22%   |
| P7          | 8      | 0.05%   | 0.05%    |
| NOT(P7)     | 16,053 | 99.95%  | 99.95%   |

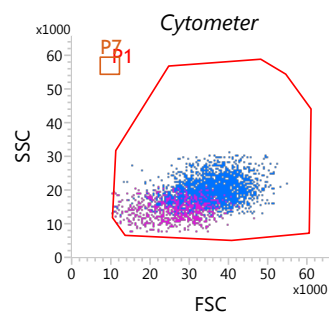

Supplement: Supplementary file 4 — This Tar/Gzip compressed file contains FANS images and gating strategies of the nuclei preparation experiments in this study. [file 41586_2020_3182_MOESM4_ESM.tgz › FANS_images/6A/CEMBA190108-6A.pdf]

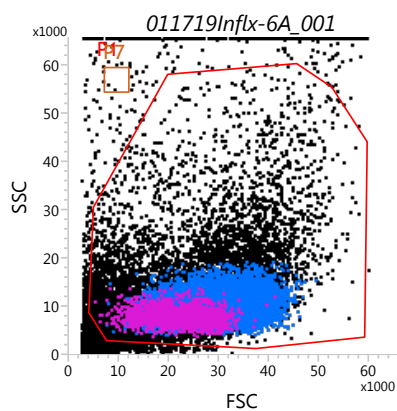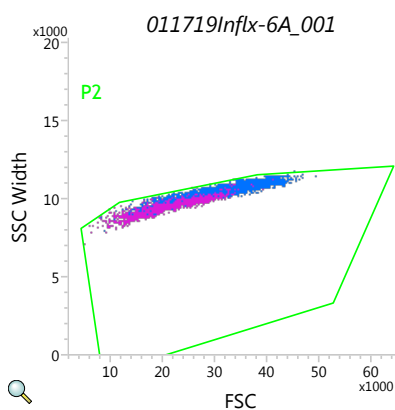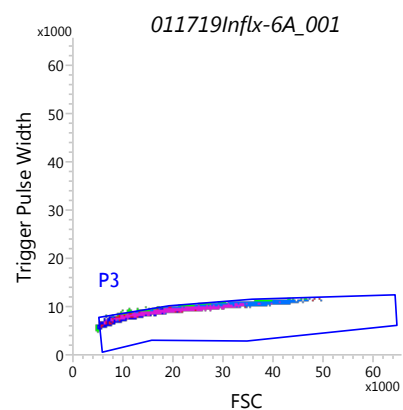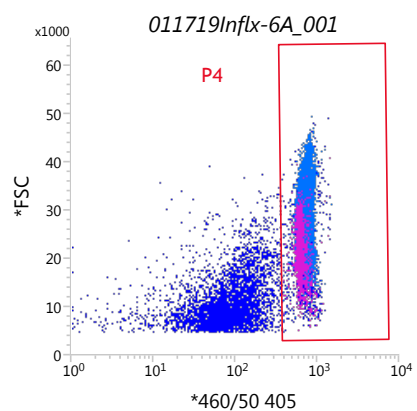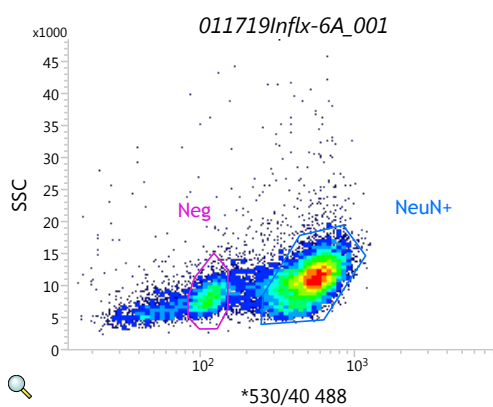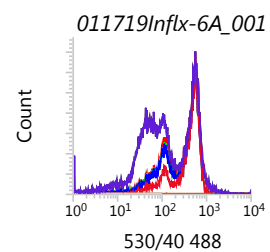

Populations: 011719Inflx-6A\_001

| Populations | Events | % Total | % Parent |
|-------------|--------|---------|----------|
| All Events  | 34,011 | 100.00% | ####     |
| P1          | 21,087 | 62.00%  | 62.00%   |
| P2          | 17,626 | 51.82%  | 83.59%   |
| P3          | 16,935 | 49.79%  | 96.08%   |
| P4          | 12,711 | 37.37%  | 75.06%   |
| NeuN+       | 9,193  | 27.03%  | 72.32%   |
| Neg         | 1,480  | 4.35%   | 11.64%   |
| P7          | 2      | 0.01%   | 0.01%    |
| NOT(P7)     | 34,009 | 99.99%  | 99.99%   |

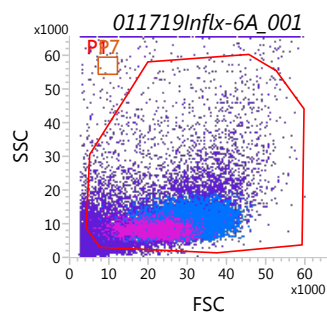

Supplement: Supplementary file 4 — This Tar/Gzip compressed file contains FANS images and gating strategies of the nuclei preparation experiments in this study. [file 41586_2020_3182_MOESM4_ESM.tgz › FANS_images/6A/CEMBA190117-6A.pdf]

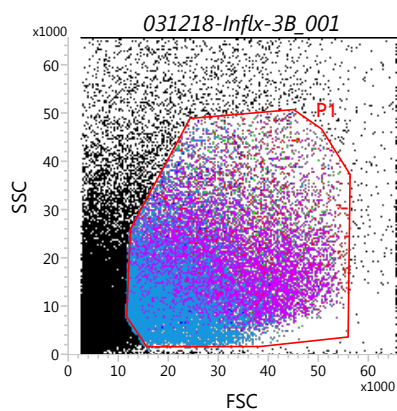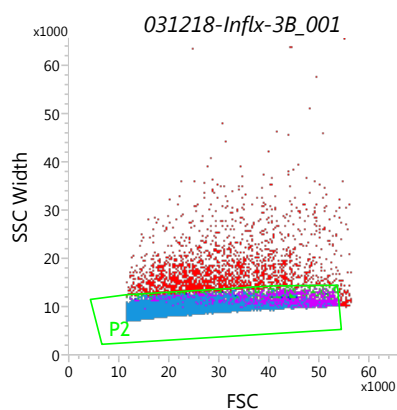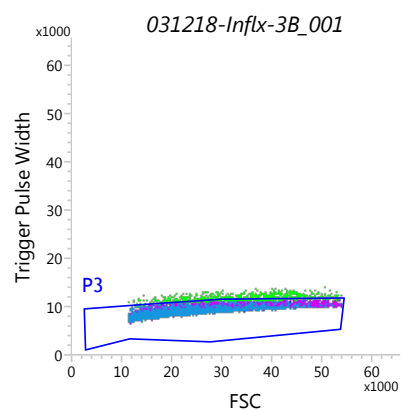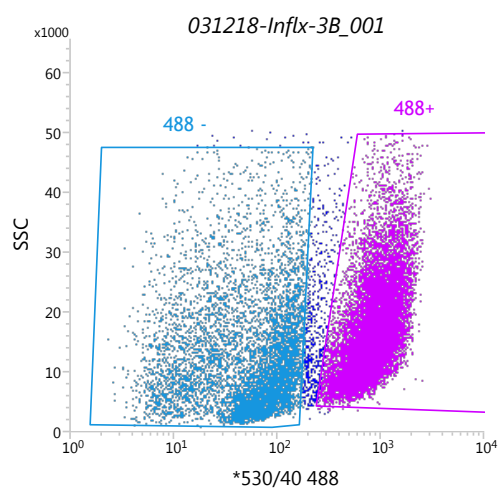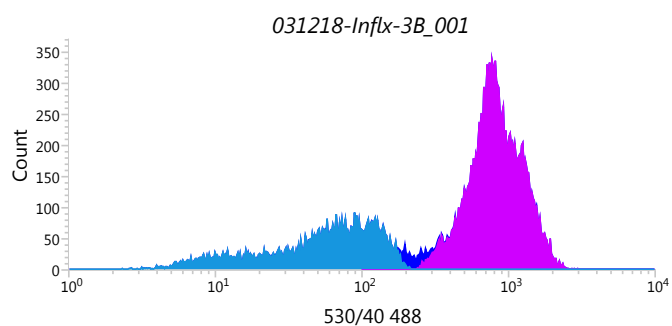

Populations: 031218-Inflx-3B\_001

| Populations | Events | % Total | % Parent |
|-------------|--------|---------|----------|
| All Events  | 59,382 | 100.00% | ####     |
| P1          | 27,024 | 45.51%  | 45.51%   |
| P2          | 24,742 | 41.67%  | 91.56%   |
| P3          | 23,884 | 40.22%  | 96.53%   |
| 488+        | 15,788 | 26.59%  | 66.10%   |
| 488 -       | 7,497  | 12.63%  | 31.39%   |

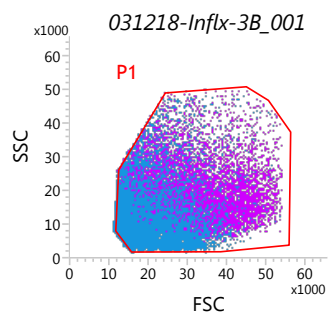

Supplement: Supplementary file 4 — This Tar/Gzip compressed file contains FANS images and gating strategies of the nuclei preparation experiments in this study. [file 41586_2020_3182_MOESM4_ESM.tgz › FANS_images/3B/CEMBA180312-3B.pdf]

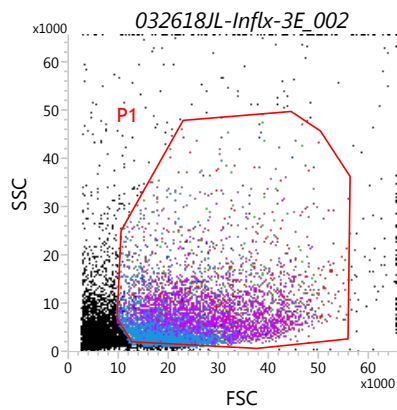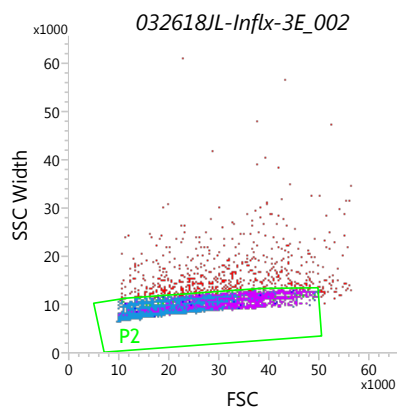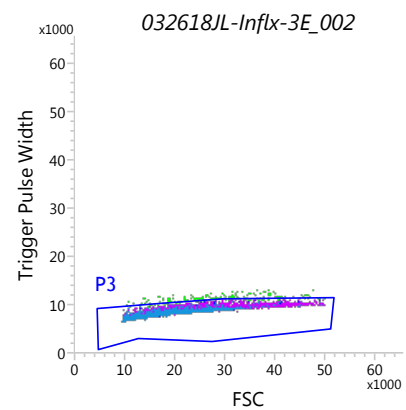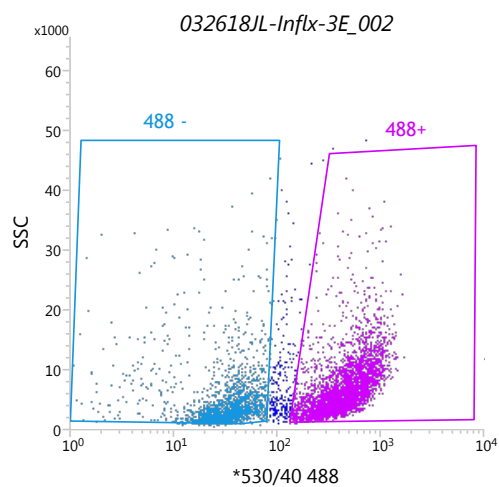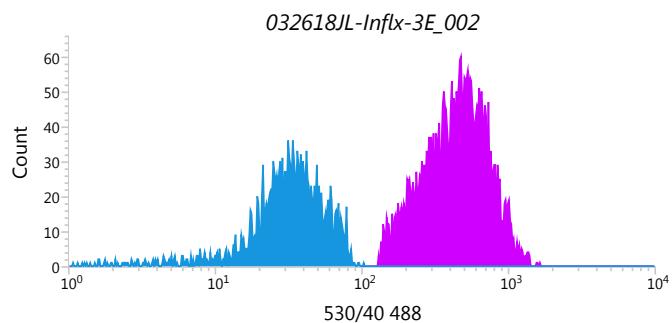

Populations: 032618JL-Inflx-3E\_002

| Populations | Events | % Total | % Parent |
|-------------|--------|---------|----------|
| All Events  | 11,372 | 100.00% | ####     |
| P1          | 6,647  | 58.45%  | 58.45%   |
| P2          | 5,964  | 52.44%  | 89.72%   |
| P3          | 5,771  | 50.75%  | 96.76%   |
| 488+        | 3,541  | 31.14%  | 61.36%   |
| 488 -       | 1,997  | 17.56%  | 34.60%   |

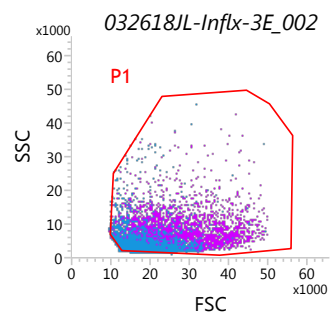

Supplement: Supplementary file 4 — This Tar/Gzip compressed file contains FANS images and gating strategies of the nuclei preparation experiments in this study. [file 41586_2020_3182_MOESM4_ESM.tgz › FANS_images/3E/CEMBA180326-3E.pdf]

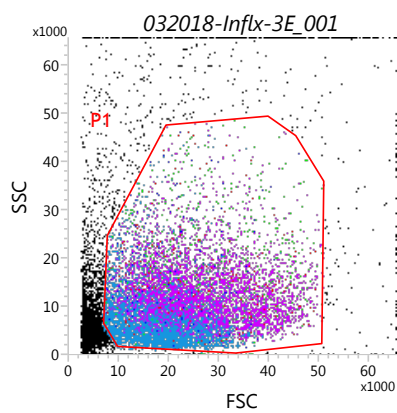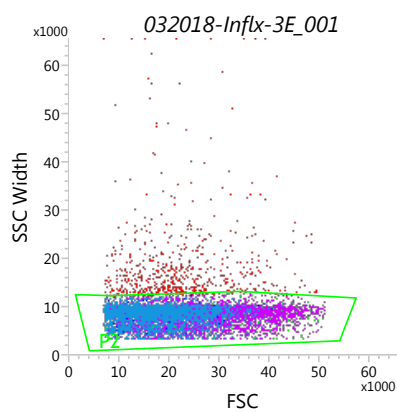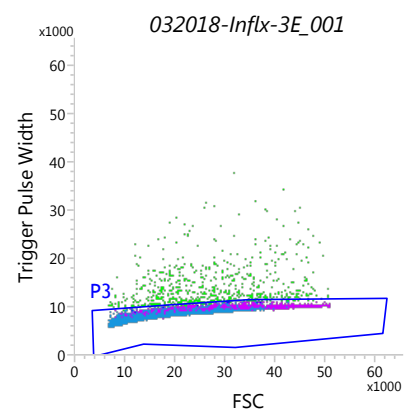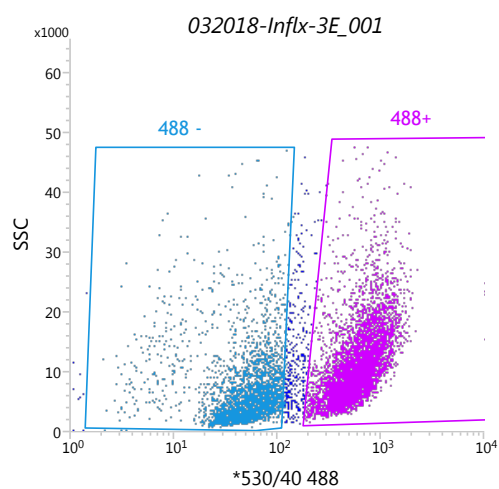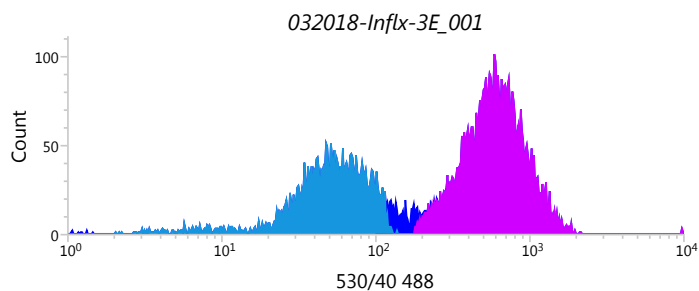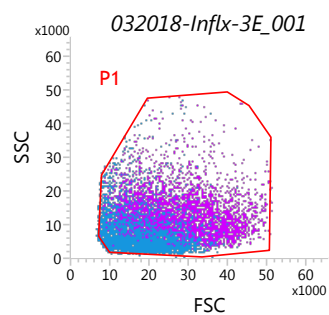

Populations: 032018-Inflx-3E\_001

| Populations | Events | % Total | % Parent |
|-------------|--------|---------|----------|
| All Events  | 13,515 | 100.00% | ####     |
| P1          | 9,862  | 72.97%  | 72.97%   |
| P2          | 9,223  | 68.24%  | 93.52%   |
| P3          | 8,544  | 63.22%  | 92.64%   |
| 488 +       | 5,195  | 38.44%  | 60.80%   |
| 488 -       | 3,017  | 22.32%  | 35.31%   |

Supplement: Supplementary file 4 — This Tar/Gzip compressed file contains FANS images and gating strategies of the nuclei preparation experiments in this study. [file 41586_2020_3182_MOESM4_ESM.tgz › FANS_images/3E/CEMBA180320-3E.pdf]
